# Analysis of functional connectivity changes from childhood to old age: A study using HCP-D, HCP-YA, and HCP-A datasets

**DOI:** 10.1162/imag_a_00503

**Published:** 2025-03-06

**Authors:** Yaotian Wang, Shuoran Li, Jie He, Lingyi Peng, Qiaochu Wang, Xu Zou, Dana L. Tudorascu, David J. Schaeffer, Lauren Schaeffer, Diego Szczupak, Jung Eun Park, Stacey J. Sukoff Rizzo, Gregory W. Carter, Afonso C. Silva, Tingting Zhang

**Affiliations:** Department of Biostatistics and Bioinformatics, Emory University, Atlanta, GA, United States; Department of Statistics, University of Pittsburgh, Pittsburgh, PA, United States; Department of Biostatistics, University of Pittsburgh, Pittsburgh, PA, United States; Department of Neurobiology, University of Pittsburgh, Pittsburgh, PA, United States; The Jackson Laboratory, Bar Harbor, ME, United States

**Keywords:** resting-state brain network, clustering, functional connectivity, lifespan changes

## Abstract

We present a new clustering-enabled regression approach to investigate how functional connectivity (FC) of the entire brain changes from childhood to old age. By applying this method to resting-state functional magnetic resonance imaging data aggregated from three Human Connectome Project studies, we cluster brain regions that undergo identical age-related changes in FC and reveal diverse patterns of these changes for different region clusters. While most brain connections between pairs of regions show minimal yet statistically significant FC changes with age, only a tiny proportion of connections exhibit practically significant age-related changes in FC. Among these connections, FC between region clusters from the same functional network tends to decrease over time, whereas FC between region clusters from different networks demonstrates various patterns of age-related changes. Moreover, our research uncovers sex-specific trends in FC changes. Females show much higher FC mainly within the default mode network, whereas males display higher FC across several more brain networks. These findings underscore the complexity and heterogeneity of FC changes in the brain throughout the lifespan.

## 1 Introduction

The human brain operates as an intricate, high-dimensional network, where network nodes denote brain regions and network edges represent connections between pairs of brain regions. Research (Jockwitz & Caspers, 2021; Jolles et al., 2011; Pruitt et al., 2024; Tomasi & Volkow, 2012) has shown that the human brain network undergoes substantial changes in both functional connectivity (FC) (Friston, 2011) and cognitive functions throughout a person’s lifespan. This has sparked a keen interest in mapping and understanding the trajectories of age-related FC changes in the entire brain.

The patterns of age-related FC changes vary across different brain regions, connections, and life stages. For example, the connectivity of a motor network increases from early to middle adulthood and then decreases subsequently (Siman-Tov et al., 2017). Meanwhile, the visual cortex shows a consistent decline in FC throughout one’s lifespan (Cao et al., 2014). Existing studies also indicated a general trend where FC between regions from the same brain functional network (within-network FC) decreases with age, whereas FC between regions from different networks (between-network FC) tends to increase (Betzel et al., 2014). These diverse patterns highlight the complexity and heterogeneity of age-related changes in the brain.

The complexity of age-related changes in FC has resulted in inconsistent findings across different studies. For example, while some research reported an increase in connectivity within the default mode network (DMN) from adolescence to adulthood (Fair et al., 2008; Fan et al., 2021; Sato et al., 2014), other studies observed a decrease in DMN connectivity with age (Betzel et al., 2014; Cao et al., 2014; Geerligs et al., 2015). Similar inconsistencies are noted for other functional networks as detailed in the review by Jockwitz and Caspers (2021). These discrepancies are partly due to the insufficient number of subjects analyzed in many of these studies, emphasizing the need for large datasets to accurately capture the heterogeneous patterns of age-related FC changes across different brain regions.

To obtain more accurate and comprehensive estimates of age-related FC changes in the entire brain, this study leveraged large datasets by analyzing resting-state functional magnetic resonance imaging (fMRI) data aggregated from three sources. Specifically, we analyzed data aggregated from the Human Connectome Project Young Adults (HCP-YA) (Van Essen et al., 2013), the Lifespan Human Connectome Project in Development (HCP-D) (Somerville et al., 2018), and the Lifespan Human Connectome Project in Aging (HCP-A) (Bookheimer et al., 2019).

Several fMRI analysis methods have been developed to evaluate age-related FC changes, including test-based comparison, independent component analysis (ICA), graph-theory-based methods, and independent regression analysis for every pair of regions. Among these, the widely used test-based comparison method typically employs statistical tests to compare functional brain networks between different age groups (Geerligs et al., 2015; Setton et al., 2023; Siman-Tov et al., 2017). However, it falls short in capturing the continuous trajectory of FC changes throughout the lifespan.

ICA (Beckmann & Smith, 2004; Cole et al., 2010; Luo et al., 2020; Smith et al., 2009) decomposes whole-brain fMRI data into independent components (ICs), each representing spatially co-activated regions. This method allows for estimating age-related FC changes for each IC (Douaud et al., 2014), but does not differentiate different connectivity trajectories between regions within the same IC, as regions grouped under one IC might not exhibit identical FC change trajectories.

Graph-theory-based approaches are widely applicable for studying diverse network characteristics such as modularity, node strength, and betweenness (Fan et al., 2021; Sporns, 2014). Yet, they are not tailored to capture distinct trajectories of age-related FC changes across different connections. Alternatively, independent regression analysis, which regresses FC against age independently for each pair of regions, provides flexibility in estimating FC trajectories that vary across different brain connections. However, independent regression analysis has low statistical power to differentiate between enormous and diverse FC trajectories due to the large number of comparisons required. To reduce the number of comparisons, common practices (Betzel et al., 2014) average FC across regions within the same functional network and consider these regions collectively, but this approach fails to account for the distinct FC trajectories that might exist among regions within the same network.

To address the shortcomings of existing methods, we developed a new approach called clustering-enabled regression. This method integrates clustering with regression models to analyze FC data, where FC measurements between pairs of brain regions are response variables, and age and sex are predictors. The clustering-enabled regression advances traditional independent regression by clustering brain regions that exhibit identical and substantial changes in FC with age, while also accounting for sex differences. As a result, this method not only identifies regions that share identical age-related FC changes but also uncovers distinct patterns of these changes across different clusters of regions. Overall, this new approach offers improved precision and comprehensiveness in mapping sex-specific trajectories of age-related FC changes across different regions.

We applied the new clustering-enabled regression to FC data derived from resting-state fMRI data of 1,673 White subjects across the three HCP studies. We identified clusters of brain regions and revealed diverse sex-specific FC trajectories between these clusters.

## 2 Methods

### 2.1 Data acquisition

Our study used resting-state fMRI data aggregated from three publicly accessible datasets: the HCP-D, HCP-YA, and HCP-A, covering a wide age range of participants.

For the cohort of healthy young adults aged between 22 and 37, we utilized the HCP-YA dataset, which includes 753 White individuals (390 females and 363 males) and 243 individuals from other or unknown racial groups (139 females and 104 males). The data were collected using a specialized 3 Tesla scanner with a 100 mT/m gradient coil at Washington University. The young adult participants completed four 15-minute resting-state fMRI scanning runs, totaling 1 hour. Resting-state images were collected with the parameters: gradient-recalled echo planar imaging sequence, 2 mm isotropic voxels, and TR/TE of 720/33.1 ms at a 52° flip angle (Harms et al., 2018; Van Essen et al., 2013).

We obtained resting-state fMRI data for individuals aged 8 to 21 years from the HCP-D Release 2.0, which initially comprised 652 participants. To ensure protocol uniformity, we excluded children aged 5 to 7 years and those with fewer than four resting-state fMRI runs. This resulted in data from 403 White subjects (222 females and 181 males) and 218 subjects from other or unknown racial groups (114 females and 104 males). These participants underwent a series of four resting-state fMRI runs, in total spanning roughly 26 minutes, conducted on 3 Tesla Siemens Prisma scanners with an 80 mT/m gradient coil. The scan parameters used in the HCP-D were similar to those used in the HCP-YA, including a 2D multiband gradient-recalled echo planar imaging sequence (MB8), 2 mm isotropic voxels, a TR/TE of 800/37 ms, and a flip angle of 52° (Harms et al., 2018; Somerville et al., 2018).

For subjects aged 36 to over 100 years, we obtained data from the HCP-A Release 2.0, excluding those with fewer than four fMRI runs. This adjusted dataset included 517 White participants (286 females and 231 males) and 197 participants from other or unknown racial groups (113 females and 84 males). Imaging was conducted on 3 Tesla Siemens Prisma scanners at four different sites, utilizing nearly identical protocols as those used in the HCP-D (Bookheimer et al., 2019; Harms et al., 2018).

### 2.2 Data preprocessing

We used the publicly available ABCD-HCP BIDS pipeline (Feczko et al., 2021; Marek et al., 2022; Sturgeon et al., 2023) to process all the raw imaging data. The ABCD-HCP BIDS pipeline consists of six stages, where the first five stages are slight modifications of the corresponding preprocessing stages in the HCP pipeline. The final stage involves the following steps: (a) Implementation of a respiratory motion filter to improve the framewise displacement (FD) assessment (Fair et al., 2020; Power et al., 2012). (b) Motion censoring based on the filtered FD evaluated in (a). Frames with FD exceeding 0.3 mm are deemed motion-contaminated and excluded in the subsequent preprocessing steps. (c) Regressing out the nuisance covariates, including the mean signal of all grayordinates and its derivative, the mean white matter signal and its derivative, the mean ventricular signal and its derivative, and the Friston 24 motion regressors, through a general linear model (Friston et al., 1996; Power et al., 2014). (d) The residuals are linearly interpolated across the censored frames identified in (b) (Power et al., 2014). (e) A temporal band-pass filter (0.009–0.08 Hz) is applied to the interpolated residuals from (d) (Power et al., 2012). The details of all preprocessing steps for raw imaging data are provided in the Supplementary Materials and Methods.

With the preprocessed imaging data, we first partitioned the brain into 360 regions using the Glasser 360 atlas (Glasser et al., 2016) and evaluated correlations between the activities of all pairs of regions. For each brain region and each fMRI run, we extracted the data of all grayordinates within the region from the preprocessed images using only frames with an FD below 0.2 mm, and averaged the extracted data across these grayordinates to obtain a single time series for the region. This process resulted in 360 time series per fMRI run, corresponding to the 360 brain regions. We then computed Pearson correlations between the time series of each pair of regions for every run. Finally, we averaged these correlations across all fMRI runs to obtain the overall correlation for each region pair.

### 2.3 Data harmonization

Although HCP-A, HCP-D, and HCP-YA datasets have similar temporal signal-to-noise ratios (Harms et al., 2018), our research uncovered notable FC differences among subjects of similar ages across these datasets, particularly between the HCP-A and HCP-YA and between the HCP-D and HCP-YA. To reconcile these site-related discrepancies, we employed ComBat (A. A. Chen et al., 2022; Yu et al., 2018) to correct the site effects in the data. Since HCP-A and HCP-D datasets employed nearly identical scanning protocols and were acquired at five different sites (Harms et al., 2018), we applied six-site ComBat to HCP-A, HCP-D, and HCP-YA datasets, treating HCP-D and HCP-A data as originating from five different sites and HCP-YA data from the sixth site.

Let $\rho_{ij}^{d_{s},s}$ denote the overall correlation (computed as described in Section 2.2) between regions i and j of subject s from site $d_{s}$, where i,j=1,…,R , s=1,…,S and $d_{s}\in \left\{1,\ldots ,6\right\}$. Here, R denotes the number of regions, S denotes the number of subjects under analysis, and $d_{s}$ represents the site from which the data of subject s was obtained.

Let $\phi_{ij}^{d_{s},s}=0.5[\text{ln}(1+\rho_{ij}^{d_{s},s})-\text{ln}(1-\rho_{ij}^{d_{s},s})]$ , that is, Fisher’s z transformed Pearson correlation coefficient. For each pair of regions i and j, we applied six-site ComBat to $\phi_{ij}^{d_{s},s}$ and standardized the post-harmonization connectivity values of all subjects to mean zero and variance one. The ensuing post-harmonization standardized connectivity values are denoted by $F_{ij}^{d_{s},s}$ . We regress $F_{ij}^{d_{s},s}$ versus subjects’ age and sex to map FC change trajectories, as detailed below.

### 2.4 Clustering-enabled regression model for FC

In our regression models, the response variable is the FC measurement, $F_{ij}^{d_{s},s}$ , and the predictors include standardized covariates: sex, age, age squared, and their respective interactions. Let $A^{s}$ and $G^{s}$ represent the age and sex of subject s, with $G^{s}\;=0$ for females and $G^{s}\;=1$ for males. We used $z^{s}\;=((A^{s},{\left(A^{s}\right)}^{2},G^{s},A^{s}\cdot G^{s},{\left(A^{s}\right)}^{2}\cdot G^{s})$ as predictors to explain variation in FC between every pair of regions. Each predictor was standardized to have a mean of zero and unit variance prior to regression analysis. Denote the ensuing post-standardization predictor values of subject s by $x^{s}\;=({\tilde{A}}^{s}{\tilde{A}}_{2}^{s},{\tilde{G}}^{s},{\tilde{AG}}^{s},{\tilde{A_{2}G}}^{s})$ .

The proposed clustering-enabled regression model identifies clusters of brain regions that display identical age-related changes in FC. Let K represent the number of brain region clusters, and let $m_{i}\;=(m_{i1},\;\;m_{i2},...,m_{iK}{)}^{\prime }$ be a K-element vector that specifies the cluster assignment of region i. Only one element of $m_{i}$ equals one, indicating the cluster of region i, and the rest elements equal zero. Furthermore, let $B_{l}$, l=1,...,5 , denote a K×K symmetric matrix with each element $B_{l,k_{1}k_{2}}$ denoting a regression coefficient for FC between clusters $k_{1}$ and $k_{2}$. We propose the following regression model for the relationship between FC with age and sex:

Fijds,s=m′i B1mj  ⋅  A˜s+m′i B2mj  ⋅  A˜2s+m′i​ B3mj  ⋅  G˜s + m′i B4mj  ⋅  AG˜s  + m′i B5mj  ⋅  A2G˜s+ϵijds,s. (1)

where $\epsilon_{ij}^{d_{s},s}$ represents the site-specific error term. Note that intercept is not included in the above model, because $F_{ij}^{d_{s},s}$ and all predictors are already standardized to have mean zero. The error term, $\epsilon_{ij}^{d_{s},s}$ , follows a normal distribution with a site-specific variance:

$$\begin{array}{cccc} \epsilon_{ij}^{d_{s},s}∼N(0,\sigma_{d_{s},ij}^{2})\;\text{and}\;\sigma_{d_{s},ij}^{2}=\sigma_{ij}^{2}\cdot \delta_{d_{s},ij}^{2}, & & & \end{array}$$ (2)

where $\delta_{d_{s},ij}^{2}$ represents the site effect on variances specific to the site $d_{s}$. We set the site of HCP-YA data as site 1 and let $\delta_{1,ij}^{2}\;=1$ to ensure identifiability.

**Prior specification.** We assign the following prior distributions to cluster labels $m_{i}$, regression coefficients $B_{l,k_{1}k_{2}}$ for l=1,…,5 , variances $\sigma_{ij}^{2}$ and $\delta_{d,ij}^{2}$. Let $p=(p_{1},\ldots ,p_{K})$ , where $0<p_{k}<1$ and $\sum_{k=1}^{K}\;p_{k}=1$ .

$$\begin{array}{cccc} \;\;\;\;{\text{m}}_{i}|\text{p} & ∼ & \text{Multinomial}\left(1,\text{p}\right),\;\;i=1\ldots ,R; & \;\;\text{(3)}\\ \;\;\;\;\;\;\;\;\text{p} & ∼ & \text{Dirichlet}\left(\frac{1}{K}1_{K}\right); & \;\;\text{(4)}\\ B_{l,k_{1}k_{2}}|\xi_{l,k_{1}k_{2}}^{2} & ∼ & \text{Normal}\left(0,\xi_{l,k_{1}k_{2}}^{2}\right),\;1\leq k_{2}\leq k_{1}\leq K; & \;\;\text{(5)}\\ \;\;\;\;\;\;\xi_{l,k_{1}k_{2}}^{2} & ∼ & \text{Inv-Gamma}\left(\rho_{0},\rho_{0}\right); & \;\;\text{(6)}\\ \;\;\;\;\;\;\sigma_{ij}^{2} & ∼ & \text{Inv-Gamma}\left(\varrho_{0},\varrho_{0}\right),\;1\leq j<i\leq R; & \;\;\text{(7)}\\ \;\;\;\;\;\delta_{d,ij}^{2} & ∼ & \text{Inv-Gamma}\left(\varrho_{0},\varrho_{0}\right),\;d=2,\ldots ,D, & \;\;\text{(8)} \end{array}$$

where $\rho_{0}=0.01$ and $\varrho_{0}=10^{-6}$ are pre-specified small positive values to yield noninformative priors, and $1_{K}$ is a K-dimensional vector with all elements equalling ones.

**The Gibbs sampler.** Denote the five predictors in the model (1) by an S×5 matrix, X. Specifically, the sth row of X is $x^{s}$, equaling $\left({\tilde{A}}^{s},\;{\tilde{A}}_{2}^{s},{\tilde{G}}^{s},{\tilde{AG}}^{s},{\tilde{A_{2}G}}^{s}\right)$ , a row vector consisting of standardized predictors of age, age squared, sex, and their respective interactions for subject s.

Let Θ denote all the parameters in the above Bayesian model (1)-(8):

Θ={mi,Bl,σij2,δd,ij2, p,ξl,k1k22, for i,j=1,…,R; l=1,…,5;  d=2,…,6; 1≤k2≤k1≤K}

Let $F_{ij}=\left(F_{ij}^{d_{1},1},\ldots ,F_{ij}^{d_{S},S}\right)$, and $\;F=\{F_{ij},\;\;1\leq j<i\leq R\}$ .

We employ the Gibbs sampler to simulate from the joint posterior distribution p(Θ|F,X) . Specifically, we sequentially simulate $\left\{\xi_{l,k_{1}k_{2}}^{2},l=1,\ldots ,\;5,\;\;1\leq k_{2}\leq k_{1}\leq K\right\}$, $\left\{\sigma_{ij}^{2},\;1\leq j<i\leq R\right\}$ , $\left\{\delta_{d,ij}^{2},\;d=2,\ldots ,\;6,\;1\leq j<i\leq R\right\}$ , $\{\beta_{k_{1}k_{2}},k_{1},$ $k_{2}\;=1,\ldots ,K\}$ , p, and $\left\{m_{i},i=1,\ldots ,R\right\}$ from their respective posterior conditional distributions, where $\beta_{k_{1}k_{2}}=(B_{1,k_{1}k_{2}},\ldots ,$ $B_{5,k_{1}k_{2}})$ . The exact formulations of the joint posterior distribution p(Θ|F,X) and all conditional posterior distributions are provided in the Supplementary Materials and Methods.

**The number of clusters and initial values.** The number of clusters K and the initial values of Θ in the Gibbs sampler are determined using the maximum likelihood estimates (MLE) obtained from an independent regression analysis. This regression was performed independently for each region pair, using $F_{ij}^{d_{s},s}$ as the response variable and $x^{s}=({\tilde{A}}^{s},{\tilde{A}}_{2}^{s},{\tilde{G}}^{s},{\tilde{AG}}^{s},{\tilde{A_{2}G}}^{s})$ as the predictors. Further details are provided below.

Let ${\hat{\beta }}_{ij}^{\text{M}}=\left({\hat{\beta }}_{1,ij}^{\text{M}},{\hat{\beta }}_{2,ij}^{\text{M}},{\hat{\beta }}_{3,ij}^{\text{M}},{\hat{\beta }}_{4,ij}^{\text{M}},{\hat{\beta }}_{5,ij}^{\text{M}}\right)\prime$ denote the MLE of the predictor coefficients in the independent regression analysis for the region pair i and j. These estimates are used to estimate the population-mean FC at a given age t. For this purpose, let $z_{t}^{\left(1\right)}=(t,t^{2},0,\;0,\;0)$ represent the predictor values for females at age t, and $z_{t}^{\left(2\right)}=(t,t^{2},1,t,t^{2})$ for males. Applying the same transformation that standardizes $z^{s}$ to $x^{s}$ on $z_{t}^{\left(1\right)}$ and $z_{t}^{\left(2\right)}$ results in transformed predictors $x_{t}^{\left(1\right)}$ and $x_{t}^{\left(2\right)}$ for females and males, respectively. The population-mean FC estimates between regions i and j for females and males at age t based on the independent regression are given by

$$\begin{array}{cccc} {\hat{FC}}_{ij}^{(1),\text{M}}\left(t\right)=x_{t}^{\left(1\right)}{\hat{\beta }}_{ij}^{\text{M}},\;\;\;\;\text{and }{\hat{FC}}_{ij}^{(2),\text{M}}\left(t\right)=x_{t}^{\left(2\right)}{\hat{\beta }}_{ij}^{\text{M}}. & & & \end{array}$$ (9)

Let ${\hat{FC}}_{ij}^{\text{M}}$ denote the vector of FC estimates obtained from independent regression analysis for the connection between regions i and j:

FC^ijM=(FC^ij(1),M(t1),FC^ij(1),M(t2),..., FC^ij(1),M(tq), FC^ij(2),M(t1),  FC^ij(2),M(t2),..., FC^ij(2),M(tq)),

where $\left(t_{1},t_{2},...,t_{q}\right)$ are evenly spaced values between ages 8 and 100.

For every pair of regions i and j, we computed the average correlation between ${\hat{FC}}_{ik}^{\text{M}}$ and ${\hat{FC}}_{jk}^{\text{M}}$ across all k≠i,j . This average correlation, denoted by $\varphi_{ij}$ , acts as a measure of similarity between the FC trajectories of regions i and j. We then compiled these similarity measures into an R×R symmetric matrix, $\Psi =(\varphi_{ij})$ , with entries $\varphi_{ij}$ for i≠j and i,j=1,...,R . Therefore, Ψ quantifies the similarity between FC trajectories of all pairs of regions.

We applied spectral clustering to the matrix Ψ and set K to be the number of clusters determined by the spectral clustering (Krzakala et al., 2013). The initial values of $m_{i}$ were also assigned based on the region clusters output by the spectral clustering. Specifically, K was set to 36 in our analysis.

### 2.5 Inferences based on posterior samples

We conducted 2,000 iterations of the Gibbs sampler and used the samples from the last 1,000 iterations to identify region clusters and estimate population-mean FC trajectories (Gelman et al., 2013). Let $\theta^{\left(n\right)}$ denote the sampled parameter θ from the nth iteration after the burn-in.

**Region cluster identification.** For each region i, we determined its cluster based on its mean clustering label:

$${\bar{m}}_{ik}=\frac{1}{1000}\sum_{n=1}^{1000}m_{ik}^{\left(n\right)}\text{ for }k=1,...,K.$$

Region i is deemed in cluster $k^{*}$ if ${\bar{m}}_{ik^{*}}>0.5$ .

**Population-mean FC trajectory estimation.** Let $\beta_{k_{1}k_{2}}^{\left(n\right)}=\left(B_{1,k_{1}k_{2}}^{\left(n\right)},\ldots ,B_{5,k_{1}k_{2}}^{\left(n\right)}\right)\prime$ , representing the regression coefficients sampled from the nth iteration for FC between cluster $k_{1}$ and cluster $k_{2}$. We estimated the population-mean FC trajectory between clusters $k_{1}$ and $k_{2}$ for females and males at age t by

FC^k1k2(1)(t)=xt(1)β¯k1k2 and FC^k1k2(2)(t)=xt(2)β¯k1k2,

respectively, where ${\bar{\beta }}_{k_{1}k_{2}}=\frac{1}{1000}\sum_{n=1}^{1000}\beta_{k_{1}k_{2}}^{\left(n\right)}.$

**Confidence intervals.** We constructed the 95% confidence intervals for the population-mean FC trajectories by using the 2.5th and 97.5th percentiles of the FC estimates obtained from the 1,000 iterations.

Let ${\hat{FC}}_{k_{1}k_{2}}^{\left(1,n\right)}\left(t\right)$ and ${\hat{FC}}_{k_{1}k_{2}}^{\left(2,n\right)}\left(t\right)$ denote the population-mean FC estimates between clusters $k_{1}$ and $k_{2}$ for females and males at age t, respectively, based on the sampled parameter $\beta_{k_{1}k_{2}}^{\left(n\right)}$ from the nth iteration:

$${\hat{FC}}_{k_{1}k_{2}}^{\left(1,n\right)}\left(t\right)=x_{t}^{\left(1\right)}\beta_{k_{1}k_{2}}^{\left(n\right)},\;\;\;\;\text{and }{\hat{FC}}_{k_{1}k_{2}}^{\left(2,n\right)}\left(t\right)=x_{t}^{\left(2\right)}\beta_{k_{1}k_{2}}^{\left(n\right)}.$$

The sets of 1,000 population-mean FC estimates for females and males from the 1,000 iterations are given by

$$\begin{array}{l} {\hat{FC}}_{k_{1}k_{2}}^{\left(1,1:1000\right)}(t)=\left\{{\hat{FC}}_{k_{1}k_{2}}^{\left(1,1\right)}(t),{\hat{FC}}_{k_{1}k_{2}}^{\left(1,2\right)}(t),...,{\hat{FC}}_{k_{1}k_{2}}^{\left(1,1000\right)}(t)\right\},\\ {\hat{FC}}_{k_{1}k_{2}}^{\left(2,1:1000\right)}(t)=\left\{{\hat{FC}}_{k_{1}k_{2}}^{\left(2,1\right)}(t),{\hat{FC}}_{k_{1}k_{2}}^{\left(2,2\right)}(t),...,{\hat{FC}}_{k_{1}k_{2}}^{\left(2,1000\right)}(t)\right\}. \end{array}$$

The 2.5th and 97.5th percentiles of these sample sets, ${\hat{\text{FC}}}_{k_{1}k_{2}}^{\left(1,1:1000\right)}\left(t\right)$ and ${\hat{\text{FC}}}_{k_{1}k_{2}}^{\left(2,1:1000\right)}\left(t\right)$, give the 95% confidence interval for the population-mean FC at age t for females and males, respectively.

### 2.6 Reproducibility evaluation

To assess the reproducibility of our findings on age-related FC changes, we first compared population-mean FC estimates obtained from the independent regression analyses of 50 pairs of non-overlapping, randomly divided subsets. Each subset consisted of data from approximately half of the total 1,673 White subjects. Specifically, for each random division r (from 1 to 50), we applied independent regression model to each of two non-overlapping subsets. The resulting population-mean FC estimates for females and males at age t, from the first and second subsets, are denoted by $\overset{⌣}{F}C_{ij}^{(1),r,1}\left(t\right)$, $\overset{⌣}{F}C_{ij}^{(2),r,1}\left(t\right)$, $\overset{⌣}{F}C_{ij}^{(1),r,2}\left(t\right)$, and $\overset{⌣}{F}C_{ij}^{(2),r,2}(t)$ , respectively.

Define $\overset{⌣}{F}C_{ij}^{r,h}$ as the vector comprising concatenated values of the mean FC estimates from the hth (h=1, 2 ) subset of the rth division for both females and males at evenly spaced ages, $\left(t_{1},t_{2},\ldots ,t_{q}\right)$ :

F⌣Cijr,h=(F⌣Cij(1),r,h(t1), F⌣Cij(1),r,h(t2),..., F⌣Cij(1),r,h(tq), F⌣Cij(2),r,h(t1), F⌣Cij(2),r,h(t2),..., F⌣Cij(2),r,h(tq))

We assessed the similarity between mean FC estimates from the two subsets by computing the correlation between $\overset{⌣}{F}C_{ij}^{r,1}$ and $\overset{⌣}{F}C_{ij}^{r,2}$ for each random division r and each pair of regions i and j. These correlations were used as a metric to assess the reproducibility of our analysis.

Additionally, we evaluated reproducibility by comparing the population-mean FC estimates obtained from the clustering-enabled regression model (1)–(8) for connections selected from two randomly divided subsets.

### 2.7 Regression model for testing racial differences in FC

To test reacial differences in FC, we introduced binary variables $R_{1}^{s}$, $R_{2}^{s}$, $R_{3}^{s}$, and $R_{4}^{s}$ to indicate the racial group of subject s. Specifically, the values $R_{1}^{s}=1$ , $R_{2}^{s}=1$ , $R_{3}^{s}=1$ , and $R_{4}^{s}=1$ indicate that subject s belongs to the Black, Asian/Hawaiian/Pacific Islander, American Indian/Alaska Native, and Multiracial groups, respectively. We used the following model to account for potential racial differences in FC:

Fijds,s=β1,ij⋅A˜s+β2,ij⋅A˜2s+β3,ij⋅G˜s+β4,ij⋅AG˜s +β5,ij⋅A2G˜s+β6,ij⋅R˜1s+β7,ij⋅R˜2s+β8,ij⋅R˜3s +β9,ij⋅R˜4s+ϵijds,s,

where $\tilde{X}$ denotes the standardized predictor corresponding to X.

We applied the above regression model independently to all pairs of regions and tested the statistical and practical significance of the binary predictors, $R_{1}^{s}$, $R_{2}^{s}$, $R_{3}^{s}$, and $R_{4}^{s}$ in explaining the variation of FC.

## 3 Results

### 3.1 Connections associated with age and sex

The 360 brain regions given by the Glasser 360 atlas lead to 64,620 (360*359/2) connections between all pairs of regions. We first performed independent regression analysis for all connections to select those whose FC variation can be significantly explained by age and sex differences, with $F_{ij}^{d_{s},s}$ as the response variable and ${\text{x}}^{s}=\left({\tilde{A}}^{s},{\tilde{A}}_{2}^{s},{\tilde{G}}^{s},{\tilde{AG}}^{s},{\tilde{A_{2}G}}^{s}\right)$ as the predictors (defined in Section 2.4). We assessed the significance of age-related predictors (i.e., age, age squared, and their interaction terms with sex), sex-related predictors (i.e., sex and its interaction terms with age and age squared), and the entire regression model for each connection using F-tests.

**Fig. 1.**
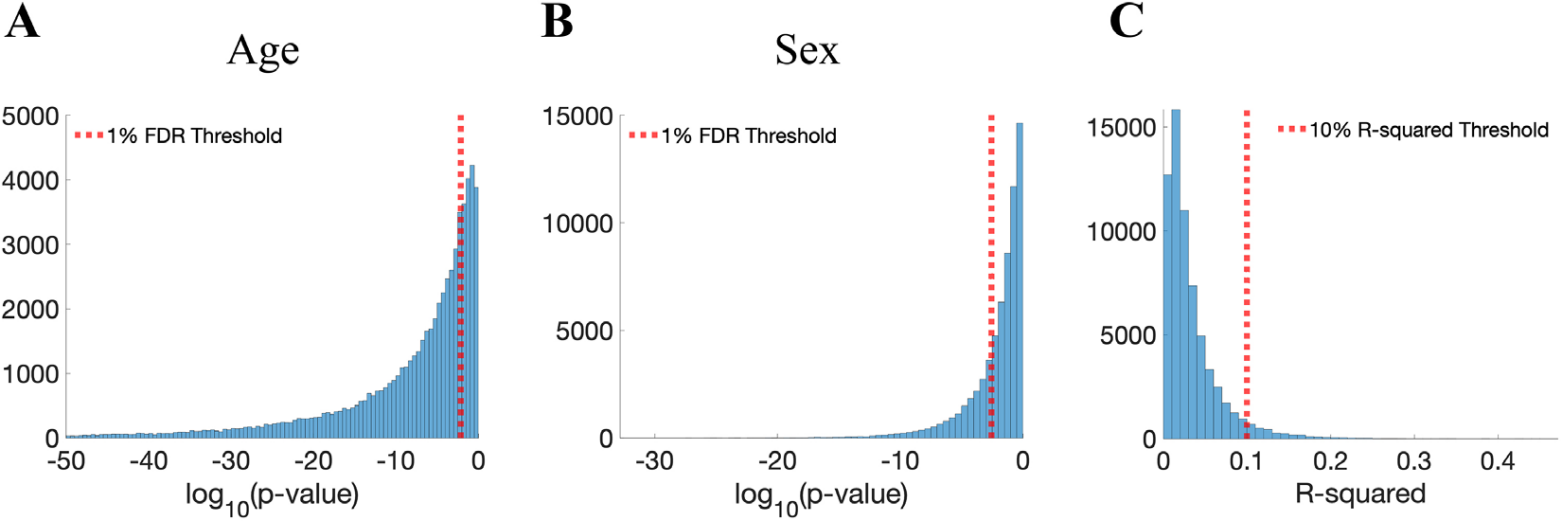
Histograms of log p-values and R-squared values from independent regression analysis for all connections, where the FC between every region pair is the response and subjects’ sex, age, age squared, and their respective interactions are predictors. (A) Histogram of log p-values of age-related predictors in the regression model for all connections. (B) Histogram of log p-values of sex-related predictors in the regression model for all connections. (C) Histogram of R-squared values of the regression model for all connections.

Figure 1 displays histograms of p-values and R-squared values from the independent linear regression analysis of FC for all connections. The analysis reveals that age-related predictors are statistically significant in the regression model for 74.2% of connections at a 1% false discovery rate (FDR) (Benjamini & Hochberg, 1995) (Fig. 1A), while sex-related predictors are significant for 28.2% of connections (Fig. 1B). In total, FC variation in 82.3% of connections can be significantly explained by age and sex differences. However, the R-squared values of these connections are predominantly low, as shown in Figure 1C. Specifically, age and sex predictors together explain less than 5% of FC variability (i.e., regression R-squared values below 5%) in 80.2% of connections and less than 10% of FC variability in 95.3% of connections. These findings indicate that in healthy subjects, only a small proportion of connections exhibit practically significant FC variations related to age and sex differences.

In this study, we focused primarily on connections with R-squared values exceeding 10% and applied the clustering-enabled regression method to these connections. This strategic choice is supported by two key reasons. First, the population-mean FC trajectories of connections with low R-squared values are nearly flat, as shown in Supplementary Figure S1. The low R-squared values and nearly flat FC trajectories suggest that these connections experience practically negligible changes in FC over time. Therefore, by concentrating on connections where FC variability can be substantially explained by age and sex, we ensure that our results capture practically meaningful variations in brain connectivity related to age and sex differences. Second, our analysis shows that estimated population-mean FC trajectories with R-squared values above 10% are highly consistent across different random subsets of the data, but this consistency decreases with lower R-squared values (detailed in Section 3.5).

### 3.2 Identified region clusters

Upon applying the clustering-enabled regression method to connections with R-squared values above 10%, we identified 28 region clusters. Of these, 24 clusters predominantly comprise regions from the same functional network. The functional networks, determined by Ji et al. (2019), include the auditory (AUD), cingulo-opercular (CON), dorsal attention (DAN), DMN, frontoparietal (FPN), language (LAN), somatomotor (SMN), visual (VIS), and ventral multimodal (VMM) networks.

The other four clusters consist of regions from multiple functional networks. However, substantial age-related changes in FC between such a cluster and certain different functional networks are mainly observed in regions that belong to the same functional network within the cluster. This observation prompted us to further subdivide these clusters into smaller ones, each limited to regions from a single functional network. This subdivision facilitated a clearer understanding of changes in FC within and between functional networks.

**Fig. 2.**
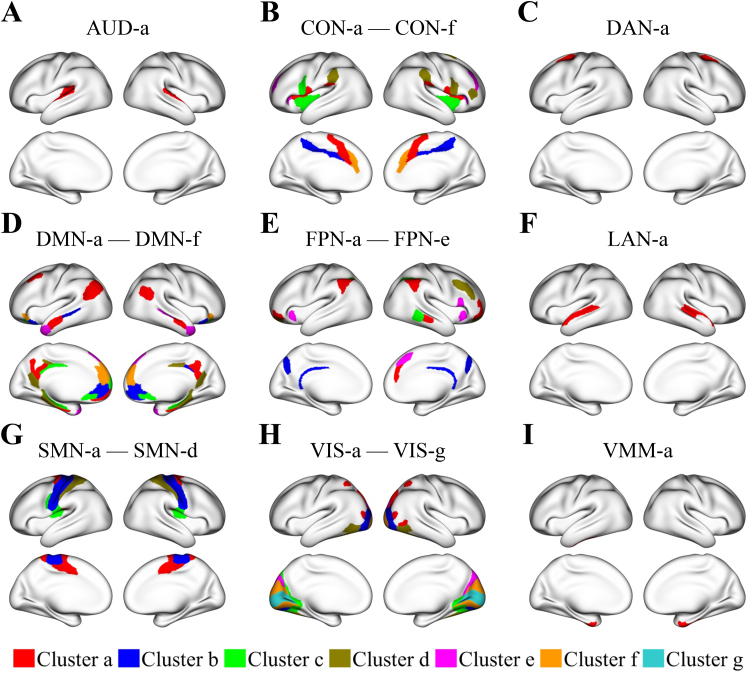
Identified region clusters, each consisting of at least four regions or 500 grayordinates from the same functional network. Within a single functional network, region clusters are plotted in different colors and labeled (e.g., a, b, etc.) in a descending order based on their region counts, from the largest to the smallest. The region clusters are displayed in an organized manner according to their respective functional networks: (A) the auditory network (AUD), (B) the cingulo-opercular network (CON), (C) the dorsal attention network (DAN), (D) the default mode network (DMN), (E) the frontoparietal network (FPN), (F) the language network (LAN), (G) the somatomotor network (SMN), (H) the visual network (VIS), and (I) the ventral multimodal network (VMM).

Figure 2 shows all sufficiently large region clusters (32 in total), each comprising a minimum of four regions or 500 grayordinates from the same functional network. Region clusters within the same functional network, such as the DMN, are labeled sequentially as DMN-a, DMN-b, and so on. The labels are assigned based on the number of regions within each cluster, from the largest to the smallest.

### 3.3 FC trajectories

Regions within the same cluster exhibit identical patterns of age-related FC changes, provided these changes meet the practical significance threshold with R-squared values above 10%. This means that connections between two region clusters with R-squared values exceeding 10% demonstrate identical FC trajectories. Therefore, for comprehensively evaluating substantial age-related FC changes across all regions, it suffices to map the practically significant FC trajectories between all region clusters. These trajectories are explained in detail below.

**Fig. 3.**
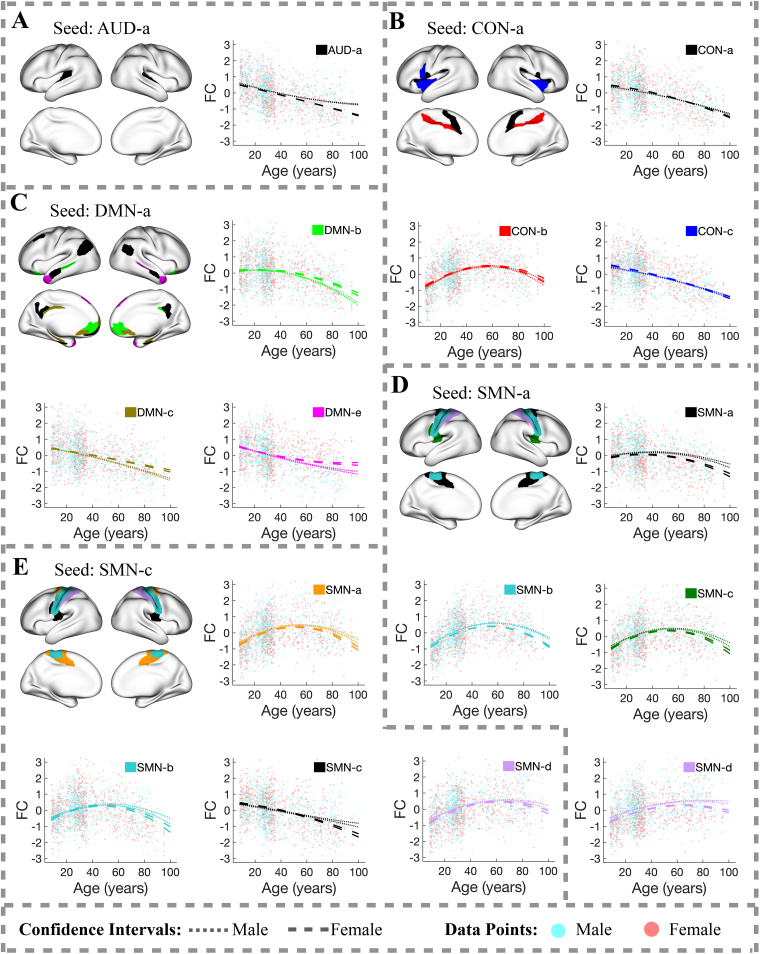
Within-network FC trajectories of the region clusters: (A) AUD-a, (B) CON-a, (C) DMN-a, (D) SMN-a, and (E) SMN-c, with R-squared values exceeding 10%. In each brain plot, one region cluster is designated as the seed cluster, plotted in black. The FC trajectory between the seed cluster and a cluster from the same functional network, referred to as cluster B, is displayed in the color assigned to cluster B. Light cyan and light coral dots in the FC plots represent FC values between a region in the seed cluster and another region in cluster B for individual male and female subjects, respectively. Dotted lines represent 95% confidence intervals for population-mean FC trajectories of males, whereas dashed lines represent those of females.

**Fig. 4.**
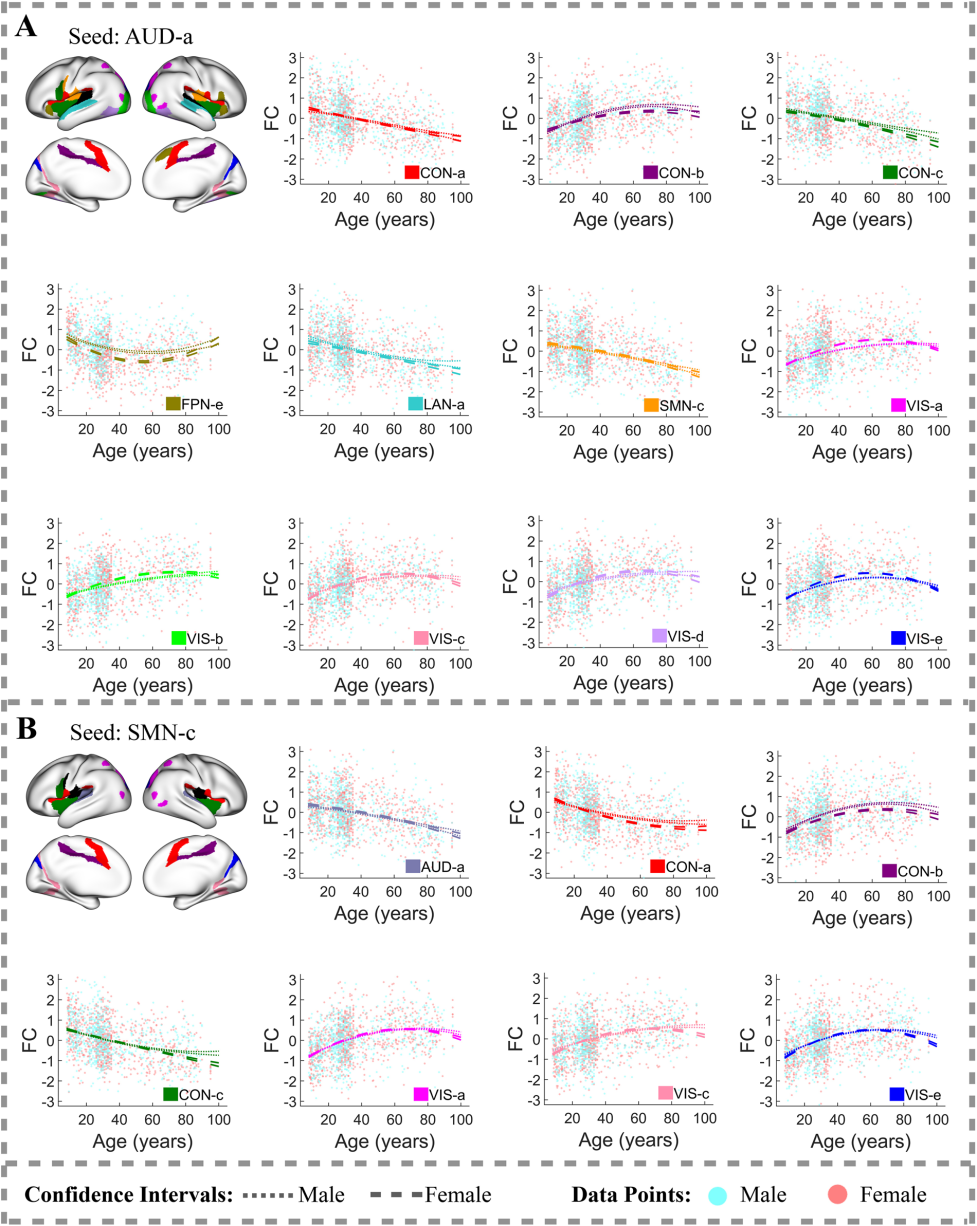
Between-network FC trajectories of the region clusters (A) AUD-a and (B) SMN-c with R-squared values exceeding 10%. In each brain plot, one region cluster is designated as the seed cluster, plotted in black. The FC trajectory between the seed cluster and another cluster from a different functional network, referred to as cluster B, is displayed in the color assigned to cluster B. Light cyan and light coral dots in the FC plots represent FC values between a region in the seed cluster and another region in cluster B for individual male and female subjects, respectively. Dotted lines represent 95% confidence intervals for population-mean FC trajectories of males, whereas dashed lines represent those of females.

**Fig. 5.**
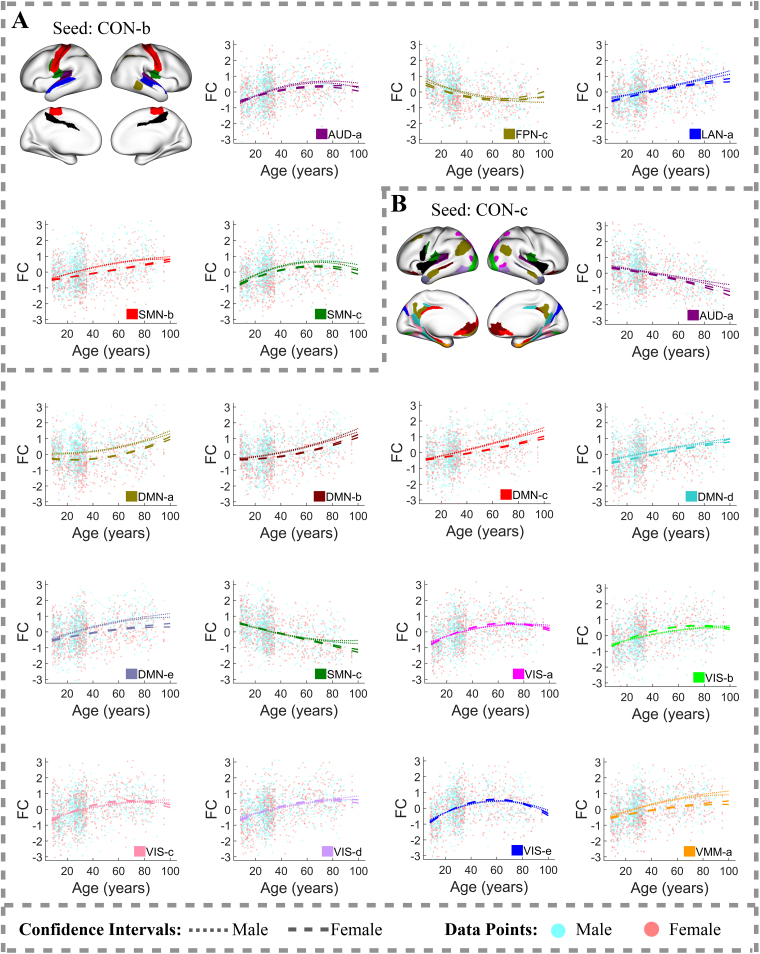
Between-network FC trajectories of the region clusters (A) CON-b and (B) CON-c with R-squared values exceeding 10%. In each brain plot, one region cluster is designated as the seed cluster, plotted in black. The FC trajectory between the seed cluster and another cluster from a different functional network, referred to as cluster B, is displayed in the color assigned to cluster B. Light cyan and light coral dots in the FC plots represent FC values between a region in the seed cluster and another region in cluster B for individual male and female subjects, respectively. Dotted lines represent 95% confidence intervals for population-mean FC trajectories of males, whereas dashed lines represent those of females.

**Fig. 6.**
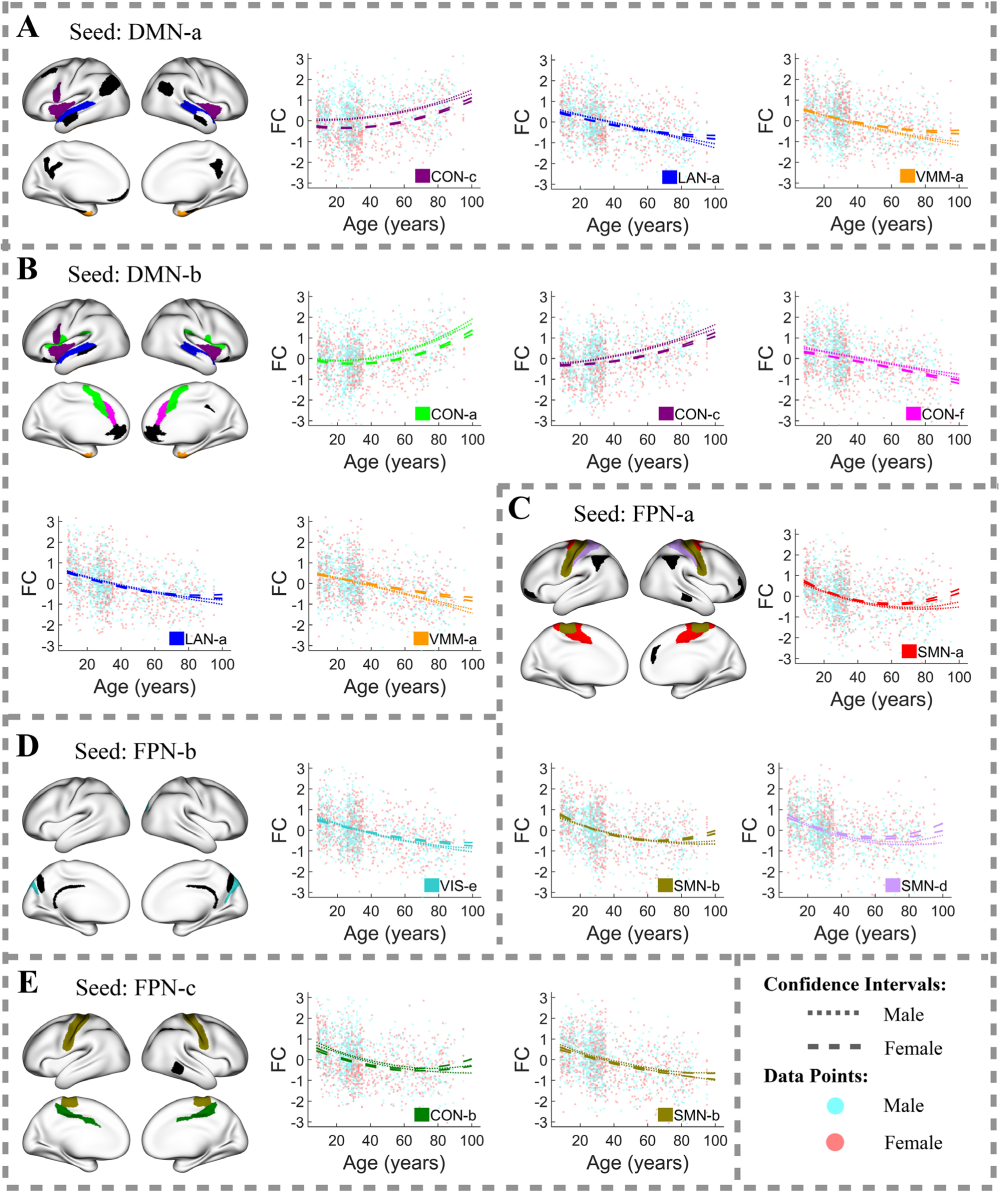
Between-network FC trajectories of the region clusters: (A) DMN-a, (B) DMN-b, (C) FPN-a, (D) FPN-b, and (E) FPN-c, with R-squared values exceeding 10%. In each brain plot, one region cluster is designated as the seed cluster, plotted in black. The FC trajectory between the seed cluster and another cluster from a different functional network, referred to as cluster B, is displayed in the color assigned to cluster B. Light cyan and light coral dots in the FC plots represent FC values between a region in the seed cluster and another region in cluster B for individual male and female subjects, respectively. Dotted lines represent 95% confidence intervals for population-mean FC trajectories of males, whereas dashed lines represent those of females.

In each of the subsequent figures illustrating practically significant FC trajectories between region clusters (Figs. 3–6), one region cluster is designated as the reference (or “seed”) cluster, displayed in black in the brain visualization plot. If more than three connections between this seed cluster and another cluster (referred to as cluster B) demonstrate practical significance in their age-related FC changes (with an R-squared value exceeding 10%), their FC trajectory is shown in the figure, color-coded to match cluster B.

Additionally, each figure also presents individual subjects’ FC values for one of the connections with R-squared values above 10% between a region in the seed cluster and another region in cluster B. These plots show the variability in FC among different subjects, with male and female subjects’ FC values represented by light cyan and light coral dots, respectively.

Although FC has large variation across subjects at all ages (Figs. 3–6), the large sample size combined with the new clustering-enabled regression used in our analysis leads to narrow 95% confidence intervals for estimated population-mean FC trajectories. Dotted lines represent 95% confidence intervals for the mean FC trajectories of males and dashed lines for those of females. To keep the visual presentation clear and emphasize the confidence intervals, the population-mean FC trajectories have been omitted from the figures.

#### 3.3.1 Within-network FC trajectories

We classify FC into two categories: within-network FC and between-network FC. Most within-network FC trajectories exhibit a steady decline with age, although some show slight increases post age 70. This declining trend is evident in connections within the AUD and DMN, as well as in some connections within the CON and SMN, as shown in Figure 3. Similar patterns of decline are also seen within the FPN (Supplementary Fig. S4D), LAN (Supplementary Fig. S4E), and VIS (Supplementary Figs. S6–S7).

Another pattern of within-network FC trajectories is an inverted U-shape, primarily seen in FC between CON clusters (Fig. 3B; Supplementary Fig. S2) and between SMN clusters (Fig. 3D–E; Supplementary Fig. S5), with peaks primarily occurring between ages 50 and 70. All within-network FC trajectories are shown in Supplementary Figures S2–S7.

In summary, our findings highlight a consistent trend of declining within-network FC with age, especially post age 50. This trend corroborates findings from prior studies on within-network FC changes in aging brains (Betzel et al., 2014).

#### 3.3.2 Between-network FC trajectories

Between-network FC trajectories display diverse patterns, unlike within-network FC trajectories. These patterns include consistent decreases, inverted U-shapes, consistent increases, and U-shapes. Specific between-network FC trajectories of different functional networks are detailed below.

Between-network FC between sensory-motor systems—AUD (Fig. 4A), SMN (Fig. 4B; Supplementary Figs. S16–S18), VIS (Supplementary Figs. S19–S21)—and several other networks, including the CON (Fig. 5; Supplementary Figs. S9–S11C), DAN (Supplementary Fig. S11D), LAN (Supplementary Fig. S15), and VMM (Supplementary Fig. S22), generally either increases steadily with age or follows an inverted U-shaped trajectory, peaking at various ages after 55. However, there are notable exceptions to these trends, particularly in FC between spatially close regions. For instance, FC between the AUD-a and CON-a/c (Fig. 4A), between the AUD-a and LAN-a (Fig. 4A), between the SMN-c and AUD-a (Fig. 4B), and between the SMN-c and CON-a/c (Fig. 4B) decreases with age across a broad age range.

Unlike the networks mentioned above, the DMN primarily exhibits decreasing between-network FC trajectories, especially in its connections with the LAN, VIS, and VMM, where FC declines over a wide age range, as illustrated in Figures 6A–B and Supplementary Figures S12–S13. Some of these trajectories, such as those between the DMN-d and several VIS clusters, have slight increases after age 65, displaying U-shaped patterns. In contrast, FC between the DMN and CON clusters consistently increases with age, as depicted in Figures 6A–B and Supplementary Figures S12–S13. The only exception is the FC between two spatially close clusters, DMN-b and CON-f (Fig. 6B), which follows a decreasing trajectory.

The FPN shows practically significant age-related FC changes in just a few of its connections, primarily with the SMN, CON, and DMN, as depicted in Figure 6C–E and Supplementary Figure S14. These between-network FC trajectories of the FPN primarily either demonstrate decreases with age or exhibit U-shaped patterns with minor increases after around age 60. Notably, the FPN-e exhibits increasing FC trajectories in its connections with DMN and VMM (Supplementary Fig. S14).

In summary, between-network FC changes from childhood to old age are characterized by diverse patterns. These patterns are not only network-specific but also dependent on the spatial proximity between brain regions.

### 3.4 Sex-related FC differences

Sex-related predictors explain less variation in FC compared to age-related predictors, achieving statistical significance at a 1% FDR in fewer (28.2%) connections. The most pronounced sex-related FC differences, where sex-related predictors explain more than 5% of FC variation after adjusting for age effects, occur in connections between the CON and DMN (Fig. 5B). Other connections also exhibit notable sex-related differences in FC, with sex-related predictors explaining more than 3% of FC variation after controlling for age effects. These include connections between the AUD-a and FPN-e (Fig. 4A), between the CON-b and SMN-b (Fig. 5A), between the CON-c and VMM-a (Fig. 5B), between the SMN-a and SMN-d (Fig. 3D), and within the DMN (Fig. 3C; Supplementary Figs. S3–S4B).

Further analysis of these findings revealed that males generally have higher population-mean FC than females across a wide age range in connections between the CON and DMN, between the AUD-a and FPN-e, between the CON-b and SMN-b, between the CON-c and VMM-a, and between the SMN-a and SMN-d. In contrast, females exhibit higher population-mean FC within the DMN throughout the lifespan.

Additionally, we observed that sex-related differences in population-mean FC increase with age in many connections, becoming most pronounced after age 80. For example, in connections within the AUD (Fig. 3A), within the SMN-c (Fig. 3E), between the CON and DMN (Figs. 5B–6B), and between the SMN and VIS (Supplementary Figs. S16–S17), elderly males show substantially higher population-mean FC compared to females. Conversely, elderly females show substantially higher population-mean FC in connections between the DMN and VMM (Fig. 6A–B) and within the DMN (Fig. 3C; Supplementary Fig. S3).

However, substantial sex-related differences in population-mean FC are not limited to oldest ages; they can also occur earlier in life. For example, in connections between the CON-c and DMN-a (Fig. 5B), between CON-b and SMN-b (Fig. 5A), and between the AUD-a and FPN-e (Fig. 4A), males show markedly higher population-mean FC starting from early life stages, with the most pronounced differences occurring between ages 45 and 60. These findings emphasize the complexity of sex-related differences in FC across different brain networks and life stages.

### 3.5 Reproducibilities of findings

To verify the robustness and reproducibility of our findings on age-related FC changes, we examined the similarity between FC trajectories estimated from analyses of non-overlapping subsets of our dataset. Specifically, we randomly split the entire dataset into two non-overlapping subsets, each comprising approximately half of the total 1,673 subjects. The same analytical procedures were then applied independently to each subset. To evaluate reproducibility, we calculated the correlations between FC trajectories obtained from each pair of subsets for every connection being studied.

We first calculated the correlations between FC trajectories derived from independent regression analyses of pairs of subsets for all connections (see Section 2.6). Specifically, the correlations for each connection were evaluated across 50 random data splits. Supplementary Figure S23A shows these correlations, plotted against the connections’ R-squared values from the independent regression analysis of the entire dataset. This figure illustrates that higher R-squared values are associated with greater consistency in FC trajectory estimates across different random subsets. For example, for connections with 10% R-squared values, the median, 25th, and 10th percentiles of the correlations were 0.94, 0.89, and 0.83, respectively. These values decreased to 0.89, 0.80, and 0.69 for connections with 5% R-squared values, and further dropped to 0.41, 0.17, and -0.05 for connections with 1% R-squared values.

We further assessed the consistency of connections selected using different R-squared thresholds across randomly divided half datasets. The median overlap coefficients (Simpson, 1960) for connections selected in pairs of random half datasets based on 10% and 5% R-squared thresholds were 0.76 and 0.77, respectively. When comparing selections from half datasets to the full dataset, these coefficients rose to 0.91 and 0.92, respectively. This indicates a high degree of consistency, with over 90% of connections chosen from the entire dataset also selected using half datasets.

Lastly, we examined the similarity of FC trajectories generated by our clustering-enabled regression method. Specifically, we calculated the correlations between the estimated FC trajectories for connections that met the 10% R-squared threshold in both subsets. The median, 25th, and 10th percentiles of these correlations were 0.97, 0.94, and 0.89, respectively. These values decreased to 0.95, 0.88, and 0.77 for the 5% R-squared threshold, as shown in Supplementary Figure S23B.

In summary, our analysis substantiates that the estimated FC trajectories for connections with R-squared values above 10% exhibit high consistency across different subsets of the data. However, this consistency decreases as the R-squared threshold lowers.

## 4 Discussion

### 4.1 Connections with minor or no age-related FC changes

Our study, leveraging a substantially larger fMRI dataset pooled from three HCP studies, provided new insights that contrast with previous research (Betzel et al., 2014), which suggested that most connections do not undergo significant (at a 1% FDR) age-related FC changes. In contrast, our analysis revealed that a majority (74.2%) of connections show statistically significant FC changes with age. However, it is important to note that while these changes are statistically significant, their practical magnitude tends to be minimal. This distinction arises because statistical significance does not always translate to practical significance, especially in large datasets where even minor changes in population-mean FC can be detected as non-zero. Our findings indicate that in healthy subjects, most connections experience only minor FC changes from childhood to old age.

Our results show that 25.8% of brain connections maintain stable FC throughout the lifespan, as age-related predictors are statistically insignificant in the regression model for these connections. Every brain region has some connections where FC does not change with age. Notably, certain regions exhibit exceptional resilience to aging. Specifically, among 360 regions analyzed, six have over 50% of their connections with stable FC, and three of these regions are in the FPN.

The FPN and posterior multimodal network (PMM) are the top two networks with the highest percentages (more than 30%) of connections with stable FC. Some research has reported no age-related changes in the FC of the FPN (Onoda et al., 2012), while other studies have observed substantial age-related FC decreases in the FPN (Huang et al., 2015; Oschmann & Gawryluk, 2020; Pruitt et al., 2024). Our analysis corroborates both findings, indicating that the magnitudes of age-related FC changes vary dramatically across different FPN regions.

The PMM, consisting of only seven regions, is a relatively new network discovered recently by Ji et al. (2019). The reason behind its age resilience warrants further exploration in future research.

### 4.2 Regions with significant age-related FC changes

Although the clustering-enabled regression method does not incorporate the spatial information of brain regions, the identified region clusters mostly consist of spatially adjacent regions. Our findings suggest that regions undergoing identical substantial age-related changes in FC are generally spatially contiguous and belong to the same functional network. This pattern is consistent with the fact that spatially proximal regions are more likely to have stronger structural and functional connections (Messé, 2020; Sepulcre et al., 2010), which may lead to identical FC changes over time.

Every brain region has some connections that undergo statistically significant age-related changes in FC. However, some regions have an exceptionally high proportion of connections that show changes in FC with age. Notably, the top three regions with the highest percentages (over 91%) of connections demonstrating significant age-related FC changes are all in the SMN. Additionally, the SMN has the highest average R-squared value for FC associated with age-related predictors. This finding is consistent with recent research indicating that age is most strongly correlated with volume changes in the SMN (Markov et al., 2022).

### 4.3 Trajectories of substantial age-related FC changes

For connections with practically significant age-related changes in FC (identified by R-squared values exceeding 10%), we observed consistent patterns of within-network FC trajectories. These trajectories typically manifest as either a steady decline or an initial increase followed by a subsequent decrease. These results resonate with several publications (Betzel et al., 2014; Luo et al., 2020), which reported similar trends. The observed decrease in FC within networks aligns with the concept of reduced functional segregation, often referred to as dedifferentiation, as a consequence of brain aging (Chan et al., 2014; Geerligs et al., 2015).

Our analysis of between-network FC uncovered more complex age-related changes than the commonly reported general increase in between-network FC with aging. We confirmed the presence of both increasing and inverted U-shaped FC trajectories, as previously reported by Betzel et al. (2014), which may act as compensatory mechanisms in response to declining within-network connectivity (Sala-Llonch et al., 2015). Moreover, we identified several decreasing between-network FC trajectories, particularly involving the DMN and FPN with other networks (Zonneveld et al., 2019).

Our study revealed that patterns of age-related FC changes are heterogeneous, not only across regions from different functional networks but also within the same network. For example, the CON-b and CON-c have distinct FC trajectories with the SMN-c (Fig. 5). These results justify the necessity for advanced methodologies that can capture such heterogeneity in brain change patterns. A notable aspect of these patterns is their relationship to the spatial proximity of the region clusters: FC between spatially adjacent regions tends to decrease with age.

The literature presented mixed findings regarding the differential age-related changes in short-distance and long-distance connectivity. Some studies reported increasing short-range FC and decreasing long-range FC with age (Sala-Llonch et al., 2014, 2015), while others observed increasing connectivity among distant regions and decreasing connectivity among neighboring regions during brain maturation (Di Martino et al., 2014; Dong et al., 2021; Fair et al., 2008; Vogel et al., 2010). Our analysis found that FC between spatially adjacent regions typically decreases with age across the lifespan. This is consistent with the findings that connectivity strength of connections shorter than 20 mm linearly decreases with age (Cao et al., 2014). This observation also aligns more closely with the commonly observed decline in within-network FC with age, suggesting that while age-related FC changes display various patterns, local connectivity tends to weaken over time.

### 4.4 Sex-related FC differences

Sex differences in functional brain networks are well-documented in the literature (Jockwitz & Caspers, 2021), with studies highlighting that women typically exhibit higher FC within the DMN (Ju et al., 2023; Ritchie et al., 2018), while men show stronger FC within the SMN (Stumme et al., 2020; Zonneveld et al., 2019). Recent studies have also demonstrated that resting-state functional networks can effectively differentiate between sexes (Ryali et al., 2024; Weis et al., 2020).

Our analysis aligns with these findings, revealing distinct FC differences between sexes. Leveraging large datasets, we identified numerous connections (28.2%) that show significant sex-related differences in population-mean FC. Moreover, our analysis traced the trajectories of FC divergence between sexes, uncovering many connections where sex-related FC differences increase with age.

These findings are crucial for advancing our understanding of sex-related behaviors and the progression of neurodegenerative diseases such as Alzheimer’s disease (AD). For example, the observation that elderly males demonstrate significantly higher FC than elderly females across multiple networks—including the CON, DMN, SMN, and VIS—may be linked to research showing that females tend to experience more rapid cognitive decline in the presence of AD (Ferretti et al., 2018). This could be because elderly females rely more heavily on connectivity within the DMN to maintain cognitive function (Ingalhalikar et al., 2014), and the DMN is known to be particularly affected by AD (Mevel et al., 2011).

The observed sex-related differences in FC, particularly between elderly females and males, may be intricately influenced by the effects of sex hormones. A substantial body of evidence highlights the critical role of sex hormones in shaping cognitive functioning across the lifespan (Gurvich et al., 2018). Among these, estrogen has garnered significant attention for its profound impact on brain function, particularly in enhancing cognitive capacities in domains such as learning and memory (Galea et al., 2017; Luine, 2014).

Animal studies further substantiate these findings, demonstrating that estrogen promotes neuroplasticity and synaptic function, which are essential for cognitive performance (Galea et al., 2017; Sheppard et al., 2019). Moreover, estrogen has been observed to exert neuroprotective effects, potentially mitigating age-related neural decline (Green & Simpkins, 2000). These effects may partially explain why the loss of estrogen during menopause is often associated with an accelerated aging phenotype, characterized by changes such as brain hypometabolism. This phenomenon is a hallmark of menopause and is often observed in prodromal AD, suggesting a link between hormonal decline and heightened susceptibility to neurodegenerative conditions (Zárate et al., 2017).

This complex interplay between sex hormones and aging underscores the importance of considering hormonal influences when studying sex differences in FC, particularly in the context of age-related cognitive decline and neurodegenerative risk. Future research exploring these mechanisms could provide valuable insights for developing targeted interventions aimed at preserving brain health and mitigating cognitive decline in aging populations.

### 4.5 FC trajectories with lower R-squared values

We applied our clustering-enabled regression analysis to connections with R-squared values above 5%, examining a wider array of connections exhibiting less pronounced age-related changes. This approach resulted in a much larger number of connections (19.8%) being included and more (34) region clusters being identified. Despite the increase, the majority of these clusters (32 out of 34) still consisted of regions within the same functional network. We subdivided the two clusters that contained regions from multiple functional networks into smaller clusters, each confined to regions from a single functional network. Consequently, we identified a total of 36 region clusters, all of which are shown in Supplementary Figure S24.

The dominant patterns in FC trajectories, both within and between networks, aligned with our earlier findings based on a 10% R-squared threshold. Within-network FC trajectories still generally exhibited a steady decline with age, as shown in Supplementary Figures S25 and S26. Several within-CON (Supplementary Fig. S25B) and within-SMN FC trajectories (Supplementary Fig. S26A) displayed inverted U-shaped patterns, echoing results from earlier analyses at the 10% R-squared threshold.

FC trajectories between networks, including the AUD, CON, DAN, LAN, SMN, VIS, and VMM (Supplementary Figs. S27–S35), typically showed either increasing patterns or inverted U-shapes. We also observed that FC trajectories between spatially close region clusters, like those between the AUD-a and CON-a/b (Supplementary Fig. S27B), AUD-a and SMN-c (Supplementary Fig. S28), CON-a and SMN-c (Supplementary Fig. S29B), DAN-a and VIS (Supplementary Fig. S30B), LAN-a and AUD-a (Supplementary Fig. S31B), and VMM-a and VIS-f (Supplementary Fig. S35A) tended to have decreasing patterns.

Our expanded analysis revealed diverse patterns in the between-network FC trajectories of the DMN (Supplementary Figs. S36–S42) and FPN (Supplementary Figs. S43–S47). The DMN continued to show predominantly increasing trajectories in its connections with the CON (Supplementary Fig. S29A), while generally displaying declining or U-shaped patterns with other networks, such as the LAN (Supplementary Fig. S31A), VIS (Supplementary Fig. S34A), and VMM (Supplementary Fig. S35A). However, we also identified several connections, like those between the DMN-e and AUD and SMN (Supplementary Fig. S40B) clusters, and between the DMN-f and VIS (Supplementary Fig. S41A) clusters, that exhibited increasing and inverted U-shaped trajectories, adding complexity to the overall picture.

Similarly, the between-network FC trajectories of the FPN with lower R-squared values also demonstrated a range of patterns. Although many of these trajectories were comparable to those with higher R-squared values—mostly showing decreasing or U-shaped trends with slight increases after age 60—there were also other distinct patterns, including increasing and inverted U-shaped trends.

In summary, while the patterns of within-network FC trajectories with less pronounced age-related changes tend to be consistent with those displaying more substantial changes, between-network FC trajectories with lower R-squared values demonstrate much greater variability, especially in connections involving the DMN and FPN. This variability highlights the importance of using larger and more comprehensive datasets in future studies to reliably capture and understand these subtle and complex age-related changes in FC.

### 4.6 Demographical factors affecting age-related FC changes

We have tried to incorporate additional demographic factors, including race, education, and family income, to explain the variation of FC. However, the inclusion of additional demographic variables is not straightforward in our analysis of the aggregated three HCP datasets. Consequently, we conducted further analyses incorporating these demographic factors as outlined below.

**Racial differences.** We applied our Bayesian method to FC measurements of White participants exclusively, as the sample sizes for other racial groups—Black, Asian/Hawaiian/Pacific Islander, American Indian/Alaska Native, and Multiracial—are significantly smaller, with very few participants from these groups aged above 80. Estimating age-related changes in FC across the lifespan for these groups using complex regression models resulted in large estimation errors, making the results unreliable. Focusing the analysis on White participants allowed us to control for potential racial differences while mitigating the impact of imbalanced sample sizes. This approach is consistent with prior studies in the field (J. Chen et al., 2024).

To explore potential racial differences in FC, we added race as one categorical predictor in the model to characterize the overall mean differences in FC between different racial groups. The detailed regression model is provided in Section 2.7. After controlling for age and sex, racial predictors were found to be statistically significant (after FDR control at 1%) in 34.82% of connections. However, for over 95% of the connections, racial predictors accounted for less than 2% of the additional variation in FC.

For the American Indian/Alaska Native and Multiracial groups, racial predictors explained more than 1% of FC variability in less than 0.01% of connections, likely due to the small sample sizes, particularly for the American Indian/Alaska Native group, which included only six participants. In the Asian/Hawaiian/Pacific Islander group, the racial predictor accounted for more than 1% of the additional FC variation in 1.69% of connections. In the Black group, the proportion increased to 12%.

Overall, the limited sample sizes for racial groups other than White precluded accurate mapping of race-specific FC trajectories. Instead, we evaluated population-mean differences by incorporating race as one categorical predictor. Future studies with larger and more balanced samples are needed to comprehensively investigate racial differences in FC trajectories across the lifespan.

**Education as a factor.** We could not comprehensively analyze the effects of education across all subjects from the three HCP datasets due to differences in the nature of this variable. For participants under 21 in the HCP-D dataset, education levels are almost linearly dependent on age, making it difficult to disentangle their effects. As a result, we restricted our evaluation of education’s association with FC to subjects in the HCP-YA and HCP-A datasets. After controlling for age and sex, we found that education had no statistically significant (at 1% FDR) association with FC for all connections.

**Socioeconomic status (family income).** We could not comprehensively analyze the effects of family income across all subjects from the three HCP datasets due to its differing interpretations across datasets: it represents the income of parents for children in the HCP-D dataset, whereas it refers to individual income for participants in the HCP-YA and HCP-A datasets. To address this, we performed separate regression analyses of FC against age, sex, and income for children (under 21 years) and adults (21 years and older). After controlling for age and sex, income was generally found to be statistically insignificant (at 1% FDR) in explaining variation in FC for both groups.

### 4.7 Other factors affecting age-related FC changes

Our analysis results demonstrate an association between FC measured by fMRI and age. However, this study does not directly reveal age-related changes in neural activity. This is primarily because FC measurements rely on the blood oxygenation level-dependent (BOLD) signal, which can be influenced by various vascular health factors that change with age. These factors include respiratory and cardiac signals (Geerligs et al., 2017), resting cerebral blood flow, cerebrovascular reactivity, BOLD-CBF coupling (Champagne et al., 2022), body mass index (BMI), blood pressure (BP), total cholesterol, APOE genotype, and others (Zonneveld et al., 2019).

To address these potential confounders, we reevaluated age-related FC changes after adjusting for vascular health factors. Since BMI, diastolic BP, and systolic BP were collected across all three HCP studies, these variables were used to account for variations in FC. Specifically, we first regressed FC against BMI, diastolic BP, and systolic BP independently for each connection. The residuals from these regressions (i.e., FC after adjusting for BMI and two BPs) were then used as new response variables, with age and sex as predictors. Additionally, separate regressions were performed for each of the three vascular factors to assess their relationships with age and sex.

Our analysis showed that BMI, diastolic BP, and systolic BP are all significantly associated with age and sex, with p-values all below $10^{-10}$ and R-squared values of 23.9%, 23.6%, and 27.0%, respectively. The associations between the three vascular factors with age and sex are illustrated in Supplementary Figure S48. Adjusting for these vascular factors reduced the significance of age-related predictors in explaining FC variation, as illustrated in Supplementary Figure S49A and D. However, the significance of sex-related predictors in explaining FC variation was overall unaffected by adjustments for BMI, diastolic, and systolic BPs, as illustrated in Supplementary Figure S49B and E.

Despite adjusting for BMI, diastolic, and systolic BPs, 62.8% of connections still exhibited a significant association between FC and age (1% FDR). Among the networks, the SMN continued to show the most pronounced age-related changes in FC from childhood to old age, with the highest average R-squared values for age-related predictors. In addition, the FPN and PMM networks still exhibited the least age-related changes in FC after accounting for these vascular factors.

The limited impact of accounting for these three vascular health factors on our findings may stem from the fact that all subjects in the analysis were mentally and physically healthy, and only three vascular factors were included in the model. Consequently, adjusting for these vascular factors had limited influence on estimated age-related FC changes. Expanding the study to include a more diverse group of subjects and a broader range of vascular health measurements could reveal stronger associations and deepen our understanding of how cardiovascular health affects age-related FC changes.

Overall, these findings highlight the complexity of interpreting age-related FC changes, as they can be influenced by a range of non-neuronal factors related to vascular health. It is crucial to differentiate the effects of aging on neural activity from those on vascular components in fMRI studies to accurately understand age-related changes in brain functions (Tsvetanov et al., 2015).

### 4.8 FC trajectories using a different brain parcellation

To assess the potential effect of the choice of brain parcellation on the analysis results, we repeated the analysis using an alternative brain parcellation, the Gordon (333) parcellation (Gordon et al., 2016). The functional networks under the Gordon parcellation (Gordon et al., 2016) include AUD, CON, DMN, FPN, DAN, VIS, and SMN, consistent with the Glasser atlas. The SMN is further divided into somatomotor hand (SMH) and somatomotor mouth (SMM) networks. Additionally, the Gordon parcellation features a cingulo-parietal network (CPN), which overlaps largely with the FPN in the Glasser atlas. The Gordon parcellation also includes retrosplenial/temporal (RST), salience (SAL), and ventral attention (VAN) networks. Regions without a clear functional identity are labeled as NONE. Most of these regions fall into the DMN and FPN in the Glasser atlas.

Upon applying the clustering-enabled regression method to connections with R-squared values above 10%, we identified 24 region clusters. Of these, 20 clusters predominantly comprise regions from the same functional network. We subdivided the four clusters that contained regions from multiple functional networks into smaller clusters, each confined to regions from a single functional network. Consequently, we identified a total of 28 region clusters, all of which are shown in Supplementary Figure S50.

Most within-network FC trajectories show a steady decline with age, and some within-network FC trajectories between the CON clusters and SMH clusters exhibit an inverted U-shape, as illustrated in Supplementary Figure S51. These patterns are consistent with findings obtained using the Glasser atlas.

Between-network FC trajectories exhibit diverse patterns, including consistent decreases, inverted U-shapes, consistent increases, and U-shapes. FC involving AUD, SMN (SMH and SMM), VIS, CON, and DAN generally either increases steadily with age or follows an inverted U-shaped trajectory. In contrast, FC between spatially close regions tends to decline with age. For example, FC between AUD-b and CON-a/b and between AUD-b and VAN-a decreases with age, as shown in Supplementary Figure S52A. The DMN primarily demonstrates decreasing between-network FC trajectories but shows increasing FC between DMN and CON clusters (Supplementary Figure S53A). These findings are consistent with results derived from the Glasser atlas.

Additionally, we consistently observed that males exhibit higher population-mean FC than females in connections between the CON and DMN (Supplementary Fig. S53A), between the CON-d and SMM-a (Supplementary Fig. S52B), between SMN clusters (Supplementary Fig. S51D), and between AUD clusters (Supplementary Fig. S51A). In contrast, females exhibit higher population-mean FC within the DMN (Supplementary Fig. S51C) throughout the lifespan.

### 4.9 Methodological advantages and contributions

Clustering connections with similar age-related changes in FC using frequentist methods is feasible. For example, one could perform edge-wide regression, apply clustering algorithms such as K-means to the estimated regression coefficients, and identify clusters of connections exhibiting comparable patterns of sex-specific age-related changes. However, the Bayesian methodology we propose offers key advantages.

First, the Bayesian approach yields more manageable and interpretable clustering results. Given at least several thousands of connections in the brain, direct clustering of connections often produces hundreds of clusters, making interpretation challenging and increasing the likelihood of misclassification. In contrast, the Bayesian method clusters regions rather than individual connections. This reduces the number of clusters to a scale consistent with the number of brain functional networks—typically a few dozen. This smaller number of region-based clusters simplifies detailed examination.

Second, the Bayesian approach facilitates deeper insights into inter-regional interactions by examining connections between region clusters. These clusters, grounded in functional networks, allow for an analysis of age-related changes in interactions within and between brain networks, offering a broader understanding of age-related changes in the brain’s functional organization. In contrast, frequentist methods, which focus on clustering individual connections, lack the framework to effectively investigate these higher-level inter-network interactions.

Finally, the Bayesian methodology explicitly accounts for uncertainty in both FC trajectory estimation and cluster identification. Quantifying this uncertainty enables the construction of confidence intervals for age-related FC changes and produces more robust and reliable results. Frequentist approaches, on the other hand, make it challenging to quantify estimation variability or evaluate the stability of clustering outcomes, limiting their robustness and inferential power.

By offering manageable and interpretable results, deeper functional insights, and robust uncertainty quantification, the Bayesian method provides a superior framework for understanding sex-specific and age-related changes in FC of the entire brain.

### 4.10 Limitations and future research

This study conducted a cross-sectional analysis using fMRI data from healthy White subjects of a wide range of ages to map the population-mean trajectories of FC changes over time. However, three primary issues are associated with this data analysis.

First, there is potential selection bias. The FC measurements of healthy individuals over 80 may not accurately represent the FC distribution for younger subjects under analysis when they reach the same age. This discrepancy can occur because “super agers,” individuals over 80 who maintain cognitive performance akin to healthy middle-aged adults, may not be representative of the general population’s aging process (Gefen et al., 2014; Rogalski et al., 2013). Consequently, the analysis provides a snapshot of what population-mean FC looks like in healthy subjects at different ages rather than depicting individual age-related FC changes.

Second, despite considerable differences in the population-mean FC between females and males across many connections, these differences do not reach statistical significance at the individual level due to extensive FC variability among individuals. This variability indicates that FC trajectories can differ significantly from person to person, suggesting the value of longitudinal studies. Such studies would track FC changes within the same individuals over time, offering deep insights into how individual differences contribute to the variability in brain functional changes with age.

Our findings indicate that age and sex explain only a small proportion of the variability in FC across the population. To deepen our understanding of the brain’s functional organization changes, integrating neuroimaging data from various modalities offers a promising path forward. A multimodal neuroimaging approach in future studies could offer a more comprehensive view of the complex processes involved in brain development and aging.

Third, a key limitation of our study is the lack of diversity in the dataset, which mainly consisted of healthy White participants. Such limitations restrict the generalizability of findings and risk overlooking critical variations in brain function and behavior across different demographic groups. Addressing this gap requires a concerted effort to improve recruitment strategies and ensure greater inclusion of underrepresented populations. Expanding diversity in research datasets is essential for generating findings that are not only more accurate but also more representative of the broader population.

## Supplementary Material

Supplementary Material

## Data Availability

The open access HCP-YA data can be downloaded from the data management platform ConnectomeDB: https://db.humanconnectome.org upon signing up for an account. Access to the restricted data of the HCP-YA study can be requested by following the guide at https://www.humanconnectome.org/study/hcp-young-adult/data-use-terms. The HCP-D and HCP-A data are available from the NIMH Data Archive (NDA): https://nda.nih.gov/general-query.html?q=query=featured-datasets:HCP%20Aging%20and%20Development. The code for the analyses presented in this paper is included as a compressed file in the supplementary materials and is also available on Github (https://github.com/StatDeptZhang/Lifespan-Changes-in-Functional-Brain-Networks).

## Supplementary Materials

Supplementary Material (created during production as a web link to online material). Supplementary material for this article is available here: https://doi.org/10.1162/imag_a_00503.
