# Supplementary material for: Analysis of functional connectivity changes from childhood to old age: A study using HCP-D, HCP-YA, and HCP-A datasets
Source: Imaging Neurosci (Camb). 2025 Mar 6;3:imag_a_00503. doi: 10.1162/imag_a_00503 (PMC11894817; doi:10.1162/imag_a_00503)
Supplement: Supplementary Material [file imag_a_00503-supp.pdf]

Supplementary File for Analysis of Functional Connectivity Changes from Childhood to Old Age: A Study Using HCP-D, HCP-YA, and HCP-A Datasets

Yaotian Wang,[1†] Shuoran Li,[2†] Jie He,[2] Lingyi Peng,[3] Qiaochu Wang,[2] Xu Zou,[2] Dana L. Tudorascu,[3] David J. Schaeffer,[4] Lauren Schaeffer,[4] Diego Szczupak,[4] Jung Eun Park,[4] Stacey J. Sukoff Rizzo,[4] Gregory W. Carter,[5] Afonso C. Silva,[4] and Tingting Zhang[2*]

[1]Department of Biostatistics and Bioinformatics, Emory University,

1518 Clifton Rd. NE, Atlanta, GA 30322, USA

[2]Department of Statistics, University of Pittsburgh,

230 S Bouquet Street, Pittsburgh, PA 15260, USA

[3]Department of Biostatistics, University of Pittsburgh,

130 De Soto Street, Pittsburgh, PA 15261, USA

[4]Department of Neurobiology, University of Pittsburgh,

200 Lothrop Street, Pittsburgh, PA 15213, USA

[5]The Jackson Laboratory, 600 Main Street, Bar Harbor, ME 04609, USA

[†]Yaotian Wang and Shuoran Li are equally contributing authors

[*]Corresponding author: Tingting Zhang, 1826 Wesley W. Posvar Hall, Room 108,

University of Pittsburgh, Pittsburgh, PA 15260, USA. Email: tiz67@pitt.edu

February 9, 2025

1 Supplementary Results

1.1 FC Trajectories with Different R-squared Values

Fig. S1 shows three statistically significant within-DMN FC trajectories (at 1% false discovery rate (FDR)) estimated by independent linear regression analysis. The R-squared values of the three FC trajectories are below 1%, around 5%, and above 10%. Despite statistical significance, the trajectories with low R-squared values are close to flat.

1.2 Practically Significant Within-Network FC Trajectories

Figs. S2-S7 illustrate all within-network FC trajectories of the connections with R-squared values above 10%. That is, age and sex explain at least 10% of FC variation in these connections. The functional networks with substantial changes in FC include the auditory (AUD), cingulo-opercular (CON), dorsal attention (DAN), default mode (DMN), frontoparietal (FPN), language (LAN), somatomotor (SMN), visual (VIS), and ventral multimodal (VMM) networks. The labels, a, b, ..., for region clusters in the same functional network are ordered based on the number of regions in each cluster from largest to smallest.

1.3 Practically Significant Between-Network FC Trajectories

Figs. S8-S22 illustrate all between-network FC trajectories of the connections with R-squared values exceeding 10%.

1.4 Reproducibilities of Findings

Fig. S23.*A* illustrates the correlations between FC trajectories obtained from independent regression analysis of 50 pairs of non-overlapping, randomly divided half datasets for all connections. These correlations are plotted against the connections' R-squared values from the independent regression analysis of the entire dataset. Fig. S23.*B* presents boxplots of correlations between FC trajectories estimated by clustering-enabled regression for connections selected with 10% and 5% R-squared thresholds in the randomly divided non-overlapping half datasets. These results confirm that estimated FC trajectories for connections with R-squared values above 10% are highly consistent across different random subsets of

the data, while this consistency decreases as the R-squared threshold lowers.

1.5 FC Trajectories with Lower R-squared Values

Upon applying the clustering-enabled regression method to connections with R-squared values above 5%, we identified 34 region clusters. Of these, 32 clusters consisted of regions from the same functional network. The remaining two clusters, which contained regions from multiple functional networks, were further subdivided into smaller clusters, each limited to regions within a single functional network. This process resulted in 36 region clusters, each comprising at least four regions or 500 grayordinates from the same functional network.

Fig. S24 shows the 36 sufficiently large region clusters. Region clusters within the same functional network, such as the DMN, are labeled sequentially as DMN-a, DMN-b, and so on. The labels are assigned based on the number of regions within each cluster, from the largest to the smallest.

Despite a wider array of connections with lower R-squared values examined, the core trends in FC trajectories, both within networks and between networks, align with earlier findings that employed the 10% threshold. As examples, we present the FC trajectories for the largest region cluster within each functional network, provided the network has FC trajectories that meet the 5% R-squared threshold.

Within-network FC trajectories predominantly show a gradual decrease with age, as depicted in Figs. S25-S26. We also observed several inverted-U shaped within-network FC trajectories, mostly within the CON (Fig. S25.*B*) and SMN (Fig. S26.*A*), which are similar to the patterns seen in within-CON and within-SMN FC trajectories with R-squared values above 10%.

Most between-network FC trajectories between the AUD (Figs. S27-S28), CON (Fig. S29), DAN (Fig. S30), LAN (Fig. S31), SMN (Figs. S32-S33), VIS (Fig. S34), and VMM (Fig. S35) have either increasing or inverted U-shaped patterns. We also observed that FC between spatially close region clusters in these networks tends to decrease with age. These include FC between the AUD-a and CON-a/b (Fig. S27.*B*), AUD-a and SMN-c (Fig. S28), CON-a and SMN-c (Fig. S29.*B*), DAN-a and VIS (Fig. S30.*B*), LAN-a and AUD-a (Fig. S31.*B*), and VMM-a and VIS-f (Fig. S35.*A*).

The analysis of between-network FC trajectories for the DMN (Figs. S36-S42) and the FPN (Figs. S43-S47), using a 5% R-squared threshold, reveals more diverse patterns compared to those identified with a 10% threshold. Although a decline in FC is still the most prevalent trend across these trajectories, a more detailed examination reveals a variety of other patterns, including increasing trends, U-shaped trajectories, and inverted U-shaped trajectories. These findings highlight the complexity and heterogeneity

of subtle age-related changes in the DMN and FPN's connectivity with other networks.

1.6 Other Factors Affecting FC Changes

Fig. S48 shows the regression of BMI, diastolic blood pressure (BP), and systolic BP versus age and sex, where each of vascular factors is the response variable and age, age squared, sex, and their interaction items are predictors. The regression models are all statistically significantly for the three vascular variables, with p-values smaller than 10^{-10}.

Fig. S49 illustrates the log p-values and R-squared values of age-related, sex-related, and all predictors in the independent regression analysis for FC of all connections, before and after adjusting for BMI, diastolic BP, and systolic BP. Panels A-C compare the log p-values of age-related, sex-related, and all predictors, respectively, without and with the inclusion of the three cardiac health factors. Panels D-F compare the R-squared values for age-related, sex-related, and all predictors, respectively, without and with the inclusion of the three cardiac health factors. After accounting for the three cardiac health factors, the significance of age-related predictors in explaining FC variation decreases for all connections, while the significance of sex-related predictors generally remains unchanged.

1.7 FC Trajectories under a Different Brain Parcellation

To assess the potential effect of the choice of brain parcellation on the analysis results, we repeated the analysis using an alternative brain parcellation, the Gordon (333) parcellation (Gordon et al., 2016). The functional networks under the Gordon parcellation (Gordon et al., 2016) include AUD, CON, DMN, FPN, DAN, VIS, and SMN, consistent with the Glasser atlas. The SMN is further divided into somatomotor hand (SMH) and somatomotor mouth (SMM) networks. Additionally, the Gordon parcellation features a cingulo-parietal network (CPN), which overlaps largely with the FPN in the Glasser atlas. The Gordon parcellation also includes retrosplenial/temporal (RST), salience (SAL), and ventral attention (VAN) networks. Regions without a clear functional identity are labeled as NONE. Most of these regions fall into the DMN and FPN in the Glasser atlas.

Upon applying the clustering-enabled regression method to connections with R-squared values above 10%, we identified 24 region clusters. Of these, 20 clusters predominantly comprise regions from the same functional network. We subdivided the four clusters that contained regions from multiple functional networks into smaller clusters, each confined to regions from a single functional network. Consequently, we identified a total of 28 region clusters, all of which are shown in Fig. S50.

Most within-network FC trajectories show a steady decline with age, and some within-network FC trajectories between the CON clusters and SMH clusters exhibit an inverted U-shape, as illustrated in Fig. S51. These patterns are consistent with findings obtained using the Glasser atlas.

Between-network FC trajectories exhibit diverse patterns, including consistent decreases, inverted U-shapes, consistent increases, and U-shapes. FC involving AUD, SMN (SMH and SMM), VIS, CON, and DAN generally either increases steadily with age or follows an inverted U-shaped trajectory. In contrast, FC between spatially close regions tends to decline with age. For example, FC between AUD-b and CON-a/b and between AUD-b and VAN-a decreases with age, as shown in Fig. S52.A. The DMN primarily demonstrates decreasing between-network FC trajectories but shows increasing FC between DMN and CON clusters (Fig. S53.A). These findings are consistent with results derived from the Glasser atlas.

Additionally, we consistently observed that males exhibit higher population-mean FC than females in connections between the CON and DMN (Fig. S53.A), between the CON-d and SMM-a (Fig. S52.B), between SMN clusters (Fig. S51.D), and between AUD clusters (Fig. S51.A). In contrast, females exhibit higher population-mean FC within the DMN (Fig. S51.C) throughout the lifespan.

2 Supplementary Materials and Methods

2.1 fMRI Preprocessing Pipeline

We used the publicly available ABCD-HCP BIDS pipeline (Feczko et al., 2021; Marek et al., 2022; Sturgeon et al., 2023) to preprocess all the raw imaging data under study. The ABCD-HCP BIDS pipeline consists of six stages, which are detailed below.

1. PreFreeSurfer: This initial stage focuses on anatomical data correction, including distortion correction, alignment, and brain extraction from the subject's native volume space. The process enhances image quality by using Advanced Normalization Tools (ANTs, (Avants et al., 2009)) to denoise images (DenoiseImage) and correct bias fields (N4BiasFieldCorrection).

2. FreeSurfer: Largely mirroring the original HCP minimal preprocessing pipeline (Glasser et al., 2013), this stage performs anatomical segmentation, reconstructs the white and pial cortical surfaces, and registers surfaces to a standard surface template (FreeSurfer's fsaverage (Fischl, 2012)).

3. PostFreeSurfer: This phase mainly creates CIFTI surface files (compatible with the HCP) and registers them to the Conte-69 surface template.

4. fMRIVolume: This stage corrects distortions and motion in spin-echo images, registers the single-band reference to the T1-weighted (T1w) image, and aligns all fMRI volumes to the anatomical data. Each fMRI volume is non-linearly registered to the MNI space.

5. fMRISurface: This stage maps volume time series onto the surface, generating CIFTI files. This process remains consistent with the original HCP minimal preprocessing pipeline.

6. The last DCANBOLDproc (DBP) stage includes multiple steps:

   6.a A DBP respiratory motion filter is applied to improve the assessment of framewise displacement (FD) (Fair et al., 2020; Power et al., 2012).

   6.b Motion censoring is conducted based on the filtered FD from Step 6.a. Frames with FD greater than 0.3 mm are considered contaminated by motion and excluded from subsequent preprocessing.

   6.c Nuisance covariates are regressed out using a general linear model. These covariates include the mean signal of all grayordinates and its derivative, the mean white matter signal and its derivative, the mean ventricular signal and its derivative, and the Friston 24 motion regressors (Friston et al., 1996; Power et al., 2014).

   6.d The residuals are linearly interpolated across the censored frames identified in Step 6.b (Power et al., 2014).

   6.e A temporal band-pass filter (0.008 - 0.09 Hz) is applied to the interpolated residuals from Step 6. d (Power et al., 2012).

After data preprocessing, we used frames with an FD below 0.2 mm to calculate the FC analyzed in this work.

2.2 The Bayesian Model for Whole-Brain FC Changes with Age

Let $F_{ij}^{d_s,s}$ denote FC (i.e., standardized Fisher's z transformed Pearson correlation) between regions i and j of subject s from database d_s, where $i, j = 1, \ldots, R$, $s = 1, \ldots, S$ and $d_s \in \{1, \ldots, D\}$. Here, R denotes the number of regions, S denotes the number of subjects, and D is the number of data sites under analysis. Let K be the number of region clusters determined based on the established procedure. Specifically, in the analysis presented, $K = 36$. Let $\boldsymbol{m}_i = (m_{i1}, m_{i2}, \ldots, m_{iK})'$ be a vector with K elements. Only one element of $\boldsymbol{m}_i$ equals one, and the rest elements equal zero, indicating the cluster of region i. For example, $m_{ik} = 1$ indicates region i falls into the kth cluster.

Let A^s and G^s represent the age and sex of subject s, with $G^s = 0$ for females and $G^s = 1$ for males. We used $\boldsymbol{z}^s = ((A^s, (A^s)^2, G^s, A^s \cdot G^s, (A^s)^2 \cdot G^s)$ as predictors to explain variation in FC between every pair of regions. Each predictor was standardized to have a mean of zero and unit variance prior to regression analysis. Denote the ensuing post-standardization predictor values of subject s by $\boldsymbol{x}^s = \left(\widetilde{A}^s, \widetilde{A_2}^s, \widetilde{G}^s, \widetilde{AG}^s, \widetilde{A_2G}^s\right)$. We propose the following regression model for the relationship between FC with age and sex:

$$\begin{aligned} F_{ij}^{d_s,s} &= \boldsymbol{m}_i'\mathbf{B}_1\boldsymbol{m}_j \cdot \widetilde{A}^s + \boldsymbol{m}_i'\mathbf{B}_2\boldsymbol{m}_j \cdot \widetilde{A_2}^s + \boldsymbol{m}_i'\mathbf{B}_3\boldsymbol{m}_j \cdot \widetilde{G}^s \\ &\quad + \boldsymbol{m}_i'\mathbf{B}_4\boldsymbol{m}_j \cdot \widetilde{AG}^s + \boldsymbol{m}_i'\mathbf{B}_5\boldsymbol{m}_j \cdot \widetilde{A_2G}^s + \epsilon_{ij}^{d_s,s}, \end{aligned} \tag{1}$$

where $\mathbf{B}_l$, $l = 1, \ldots, 5$ is a $K \times K$ symmetric matrix with each element B_{l,k_1k_2} denoting a regression coefficient for FC between a region in cluster k_1 and another region in cluster k_2, and the error term, $\epsilon_{ij}^{d,s}$, follows a normal distribution with a site-specific variance: $\epsilon_{ij}^{d_s,s} \sim N(0, \sigma^2_{d_s,ij})$.

We let $\sigma^2_{d_s,ij} = \sigma^2_{ij} \cdot \delta^2_{d_s,ij}$, where $\delta^2_{d_s,ij}$ represents the site effect on variances specific to the dataset d_s. We set the HCP-YA data as database 1 and let $\delta^2_{1,ij} = 1$ to ensure identifiability.

We first write the proposed clustering-enabled regression model [1] in a matrix form to facilitate the following derivations. Denote the five predictors in the model [1] by a $S \times 5$ matrix, $\mathbf{X}$. Specifically, the sth row of $\mathbf{X}$ equals $\boldsymbol{x}^s = \left(\widetilde{A}^s, \widetilde{A_2}^s, \widetilde{G}^s, \widetilde{AG}^s, \widetilde{A_2G}^s\right)$.

For every pair of regions i and j, let $\boldsymbol{F}_{ij} = (F_{ij}^{d_1,1}, \ldots, F_{ij}^{d_S,S})$ and $\boldsymbol{\epsilon}_{ij} = (\epsilon_{ij}^{d_1,1}, \ldots, \epsilon_{ij}^{d_S,S})$. The matrix form of Model [1] is given by

$$\boldsymbol{F}_{ij} = \mathbf{X} \cdot \left(\boldsymbol{m}_i'\mathbf{B}_1\boldsymbol{m}_j, \ldots, \boldsymbol{m}_i'\mathbf{B}_5\boldsymbol{m}_j\right)' + \boldsymbol{\epsilon}_{ij}, \ 1 \le j < i \le R, \tag{2}$$

$$\mathbf{B}_l = (\mathbf{B}_l)', \ l = 1, \ldots, 5, \tag{3}$$

$$\boldsymbol{\epsilon}_{ij} \sim \text{MVNormal}\left(\mathbf{0}, \mathbf{R}_{ij}\right), \ s = 1, \ldots, S, \tag{4}$$

where $\mathbf{R}_{ij} = \sigma^2_{ij} \cdot \mathbf{R}_{0,ij}$ and $\mathbf{R}_{0,ij}$ is an $S \times S$ diagonal matrix. The (s,s)th entry of $\mathbf{R}_{0,ij}$ equals $\delta^2_{d,ij}$ if subject s is from database d.

2.2.1 Prior Specification

We assign the following prior distributions to cluster labels $\boldsymbol{m}_i$, probability parameters B_{l,k_1k_2}, variances σ^2_{ij}, and $\delta^2_{d,ij}$. Let $\boldsymbol{p} = (p_1, \ldots, p_K)$, where $0 < p_l < 1$ and $\sum_{l=1}^K p_l = 1$.

$$
\begin{aligned}
\boldsymbol{m}_i|\boldsymbol{p} &\sim \text{Multinomial}(1, \boldsymbol{p}),\ i = 1\ldots, R; && (5)\\
\boldsymbol{p} &\sim \text{Dirichlet}(\frac{1}{K}\mathbf{1}_K); && (6)\\
B_{l,k_1k_2}|\xi^2_{l,k_1k_2} &\sim \text{Normal}(0, \xi^2_{l,k_1k_2}),\ 1 \le k_2 \le k_1 \le K,\ l = 1, \ldots, 5; && (7)\\
\xi^2_{l,k_1k_2} &\sim \text{Inverse-Gamma}(\rho_0, \rho_0); && (8)\\
\sigma^2_{ij} &\sim \text{Inverse-Gamma}(\varrho_0, \varrho_0),\ 1 \le j < i \le R; && (9)\\
\delta^2_{d,ij} &\sim \text{Inverse-Gamma}(\varrho_0, \varrho_0),\ d = 2, \ldots, D, && (10)
\end{aligned}
$$

where $\rho_0 = 0.01$ and $\varrho_0 = 10^{-6}$ are pre-specified small positive values to yield noninformative priors, and $\mathbf{1}_K$ is a K-dimensional vector with all elements equalling ones.

We use $\boldsymbol{\Theta}$ to denote all the model parameters in the above Bayesian model [2-10]:

$$\boldsymbol{\Theta} = \{\boldsymbol{m}_i, \mathbf{B}_l, \sigma^2_{ij}, \delta^2_{d,ij}, \boldsymbol{p}, \xi^2_{l,k_1k_2},\ \text{for } i, j = 1, \ldots, R;\ l = 1, \ldots, 5;\ d = 2, \ldots, 6;\ k_1, k_2 = 1, \ldots, K\}.$$

Let $\mathbf{F} = \{\boldsymbol{F}_{ij},\ 1 \le j < i \le R\}$ and $\boldsymbol{b}_{ij} = \Big(\boldsymbol{m}_i'\mathbf{B}_1\boldsymbol{m}_j, \ldots, \boldsymbol{m}_i'\mathbf{B}_5\boldsymbol{m}_j\Big)'$. Define an indicator function $1_{\mathbb{C}}(u)$, which equals one if $u \in \mathbb{C}$, and zero otherwise. The joint posterior distribution of $\boldsymbol{\Theta}$ given $\mathbf{F}$ and $\mathbf{X}$ is

$$
\begin{aligned}
& p(\boldsymbol{\Theta}|\mathbf{F}, \mathbf{X}) \propto p(\mathbf{F}|\boldsymbol{\Theta}, \mathbf{X}) \cdot p(\boldsymbol{\Theta})\\
\propto & \prod_{1\le j<i\le R} (\sigma^2_{ij})^{-S/2} \cdot \text{det}(\mathbf{R}_{0,ij})^{-1/2} \cdot \exp\Big\{-\frac{1}{2}[\boldsymbol{F}_{ij} - \mathbf{X}\boldsymbol{b}_{ij}]'(\mathbf{R}_{ij})^{-1}[\boldsymbol{F}_{ij} - \mathbf{X}\boldsymbol{b}_{ij}]\Big\}\\
& \cdot \prod_{i=1}^R \prod_{k=1}^K p_k^{m_{ik}} \cdot \prod_{k=1}^K \Big\{p_k^{1/K-1} \cdot 1_{0<p_k<1}(p_k)\Big\} \cdot 1_{\sum p_k = 1}(\boldsymbol{p})\\
& \cdot \prod_{l=1}^5 \prod_{1\le k_2\le k_1\le K} (\xi^2_{l,k_1k_2})^{-1/2} \cdot \exp\Big\{-\frac{1}{2\xi^2_{l,k_1k_2}} B^2_{l,k_1k_2}\Big\}\\
& \cdot \prod_{l=1}^5 \prod_{1\le k_2\le k_1\le K} (\xi^2_{l,k_1k_2})^{-(1+\rho_0)} \cdot \exp\Big\{-\frac{\rho_0}{\xi^2_{l,k_1k_2}}\Big\}) \cdot \prod_{1\le j<i\le R} (\sigma^2_{ij})^{-(1+\varrho_0)} \cdot \exp\Big\{-\frac{\varrho_0}{\sigma^2_{ij}}\Big\})\\
& \cdot \prod_{d=2}^D \prod_{1\le j<i\le R} (\delta^2_{d,ij})^{-(1+\varrho_0)} \cdot \exp\Big\{-\frac{\varrho_0}{\delta^2_{d,ij}}\Big\}). \qquad (11)
\end{aligned}
$$

2.2.2 The Gibbs Sampler Procedure

We use the Gibbs Sampler to simulate from [11]. The initial values of $\mathbf{\Theta}$ and the number of clusters K in the Gibbs Sampler are determined using the MLE derived from the independent regression model (1) from the main text. We explain the simulation steps in the following.

A. The conditional distribution of $\boldsymbol{m}_i$. We simulate $\boldsymbol{m}_i$ in sequence. Let $\boldsymbol{e}_k$ be a K-dimensional binary vector where only the kth entry is one.

For each i, define

$$\begin{aligned}
\boldsymbol{b}_{ij}(k) &= \Big(\boldsymbol{m}_i'\mathbf{B}_1\boldsymbol{m}_j, \ldots, \boldsymbol{m}_i'\mathbf{B}_5\boldsymbol{m}_j\Big)'\Big|_{\boldsymbol{m}_i=\boldsymbol{e}_k}, \\
\boldsymbol{b}_{ji}(k) &= \Big(\boldsymbol{m}_j'\mathbf{B}_1\boldsymbol{m}_i, \ldots, \boldsymbol{m}_j'\mathbf{B}_5\boldsymbol{m}_i\Big)'\Big|_{\boldsymbol{m}_i=\boldsymbol{e}_k}, \\
f_i(k) &= \sum_{1\le j<i}\Big\{-\frac{1}{2}[\boldsymbol{F}_{ij} - \mathbf{X}\boldsymbol{b}_{ij}(k)]'(\mathbf{R}_{ij})^{-1}[\boldsymbol{F}_{ij} - \mathbf{X}\boldsymbol{b}_{ij}(k)]\Big\} \\
&\quad + \sum_{i<j\le R}\Big\{-\frac{1}{2}[\boldsymbol{F}_{ji} - \mathbf{X}\boldsymbol{b}_{ji}(k)]'(\mathbf{R}_{ji})^{-1}[\boldsymbol{F}_{ji} - \mathbf{X}\boldsymbol{b}_{ji}(k)]\Big\} \\
&\quad + \log(\boldsymbol{p}_k).
\end{aligned}$$

We simulate $\boldsymbol{m}_i$ from

$$\text{Multinomial}\Big(1,\ \Big(\frac{\exp\{f_i(1)\}}{\sum_{k=1}^K \exp\{f_i(k)\}}, \ldots, \frac{\exp\{f_i(K)\}}{\sum_{k=1}^K \exp\{f_i(k)\}}\Big)'\Big).$$

B.The conditional distribution of ξ^2_{l,k_1k_2}. We sample ξ^2_{l,k_1k_2} from

$$\text{Inverse-Gamma}(\rho_0 + \frac{1}{2},\ \rho_0 + \frac{1}{2}B^2_{l,k_1k_2}).$$

C.The conditional distribution of $\boldsymbol{\beta}_{k_1k_2}$. Let $\boldsymbol{\beta}_{k_1k_2} = (B_{1,k_1k_2}, \ldots, B_{5,k_1k_2})'$. We sequentially sample $\boldsymbol{\beta}_{k_1k_2}$ from

$$\text{MVNormal}\Big((\mathbf{U}_{k_1,k_2})^{-1}(\mathbf{V}_{k_1,k_2})',\ (\mathbf{U}_{k_1,k_2})^{-1}\Big),$$

where

$$\begin{aligned}\mathbf{U}_{k_1,k_2} &= \sum_{\substack{\{j<i:m_{ik_1}=1,m_{jk_2}=1\}\\ \cup\{j<i:m_{jk_1}=1,m_{ik_2}=1\}}} \mathbf{X}'(\mathbf{R}_{ij})^{-1}\mathbf{X} + \text{diag}\Big(\frac{1}{\xi^2_{1,k_1k_2}}, \ldots, \frac{1}{\xi^2_{5,k_1k_2}}\Big),\\ \mathbf{V}_{k_1,k_2} &= \sum_{\substack{\{j<i:m_{ik_1}=1,m_{jk_2}=1\}\\ \cup\{j<i:m_{jk_1}=1,m_{ik_2}=1\}}} \boldsymbol{F}'_{ij}(\mathbf{R}_{ij})^{-1}\mathbf{X}.\end{aligned}$$

D. The conditional distribution of $\boldsymbol{p}$. We sample $\boldsymbol{p}$ from

$$\text{Dirichlet}(\sum_{i=1}^{R}\boldsymbol{m}_i + \frac{1}{K}\mathbf{1}_K).$$

E. The conditional distribution of σ^2_{ij}. We sequentially simulate $\sigma^2_{ij},\ j<i$ from

$$\text{Inverse-Gamma}(\varrho_0 + \frac{S}{2},\ \varrho_0 + \rho_\sigma),$$

where

$$\rho_\sigma = \frac{1}{2}(\boldsymbol{F}_{ij} - \mathbf{X}\boldsymbol{b}_{ij})'(\mathbf{R}_{0,ij})^{-1}(\boldsymbol{F}_{ij} - \mathbf{X}\boldsymbol{b}_{ij}).$$

F. The conditional distribution of $\delta^2_{d,ij}$. We simulate $\delta^2_{d,ij},\ j<i,\ d>2$ in sequence. For any S-dimensional parameter $\boldsymbol{\theta}$, we use $\boldsymbol{\theta}_{(d)}$ to denote its subset that corresponds to database d and use $S_{(d)}$ to denote the number of subjects from database d.

Define

$$\rho_\delta = \frac{1}{2\sigma^2_{ij}}(\boldsymbol{F}_{ij} - \mathbf{X}\boldsymbol{b}_{ij})'_{(d)}(\boldsymbol{F}_{ij} - \mathbf{X}\boldsymbol{b}_{ij})_{(d)}.$$

We sample $\delta^2_{d,ij}$ from

$$\text{Inverse-Gamma}(\varrho_0 + \frac{1}{2}S_{(d)},\ \varrho_0 + \rho_\delta).$$

References

Avants, B. B., Tustison, N., Song, G., et al. (2009). Advanced normalization tools (ants). Insight j, 2(365), 1–35.

Fair, D. A., Miranda-Dominguez, O., Snyder, A. Z., Perrone, A., Earl, E. A., Van, A. N., Koller, J. M., Feczko, E., Tisdall, M. D., van der Kouwe, A., et al. (2020). Correction of respiratory artifacts in mri head motion estimates. Neuroimage, 208, 116400.

Feczko, E., Conan, G., Marek, S., Tervo-Clemmens, B., Cordova, M., Doyle, O., Earl, E., Perrone, A., Sturgeon, D., Klein, R., et al. (2021). Adolescent brain cognitive development (abcd) community mri collection and utilities [Preprint at https://doi.org/10.1101/2021.07.09.451638].

Fischl, B. (2012). Freesurfer. Neuroimage, 62(2), 774–781.

Friston, K. J., Williams, S., Howard, R., Frackowiak, R. S., & Turner, R. (1996). Movement-related effects in fmri time-series. Magnetic resonance in medicine, 35(3), 346–355.

Glasser, M. F., Sotiropoulos, S. N., Wilson, J. A., Coalson, T. S., Fischl, B., Andersson, J. L., Xu, J., Jbabdi, S., Webster, M., Polimeni, J. R., et al. (2013). The minimal preprocessing pipelines for the human connectome project. Neuroimage, 80, 105–124.

Gordon, E. M., Laumann, T. O., Adeyemo, B., Huckins, J. F., Kelley, W. M., & Petersen, S. E. (2016). Generation and evaluation of a cortical area parcellation from resting-state correlations. Cerebral cortex, 26(1), 288–303.

Marek, S., Tervo-Clemmens, B., Calabro, F. J., Montez, D. F., Kay, B. P., Hatoum, A. S., Donohue, M. R., Foran, W., Miller, R. L., Hendrickson, T. J., et al. (2022). Reproducible brain-wide association studies require thousands of individuals. Nature, 603(7902), 654–660.

Power, J. D., Barnes, K. A., Snyder, A. Z., Schlaggar, B. L., & Petersen, S. E. (2012). Spurious but systematic correlations in functional connectivity mri networks arise from subject motion. Neuroimage, 59(3), 2142–2154.

Power, J. D., Mitra, A., Laumann, T. O., Snyder, A. Z., Schlaggar, B. L., & Petersen, S. E. (2014). Methods to detect, characterize, and remove motion artifact in resting state fmri. Neuroimage, 84, 320–341.

Sturgeon, D., Earl, E., Madison, T., Perrone, A., Kathy, Rueter, A., Houghton, A., & Rockets2theMoon. (2023). DCAN-Labs/abcd-hcp-pipeline [Avaiable at https://doi.org/10.5281/zenodo.7636985].

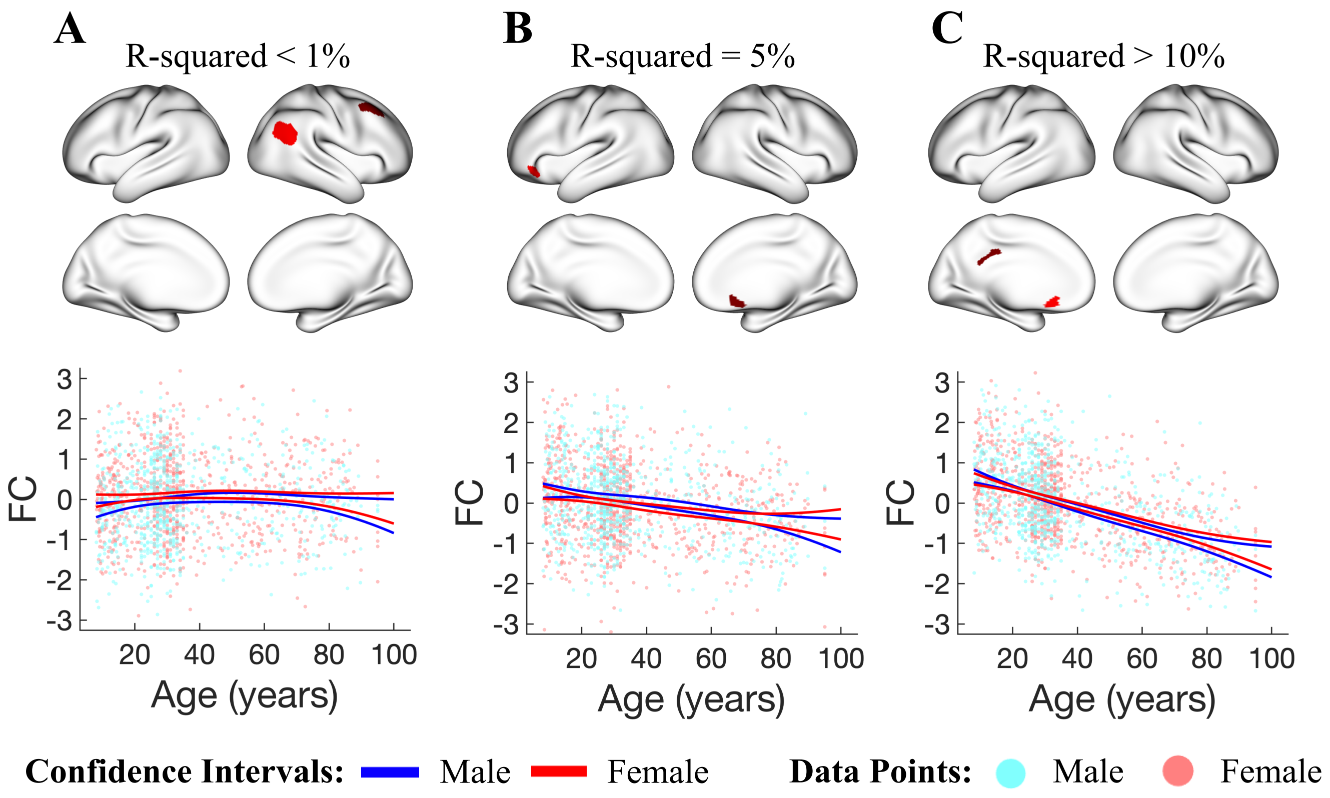

Figure S1: Three FC trajectories estimated using independent regression for three within-DMN connections. The brain plots indicate the locations of the two regions involved in each connection. While all three FC trajectories are statistically significant, they differ in their R-squared values. Light cyan and light coral dots represent the FC measurements for male and female subjects, respectively. Blue and red lines depict the 95% confidence intervals for the population-mean FC trajectories for males and females, respectively.

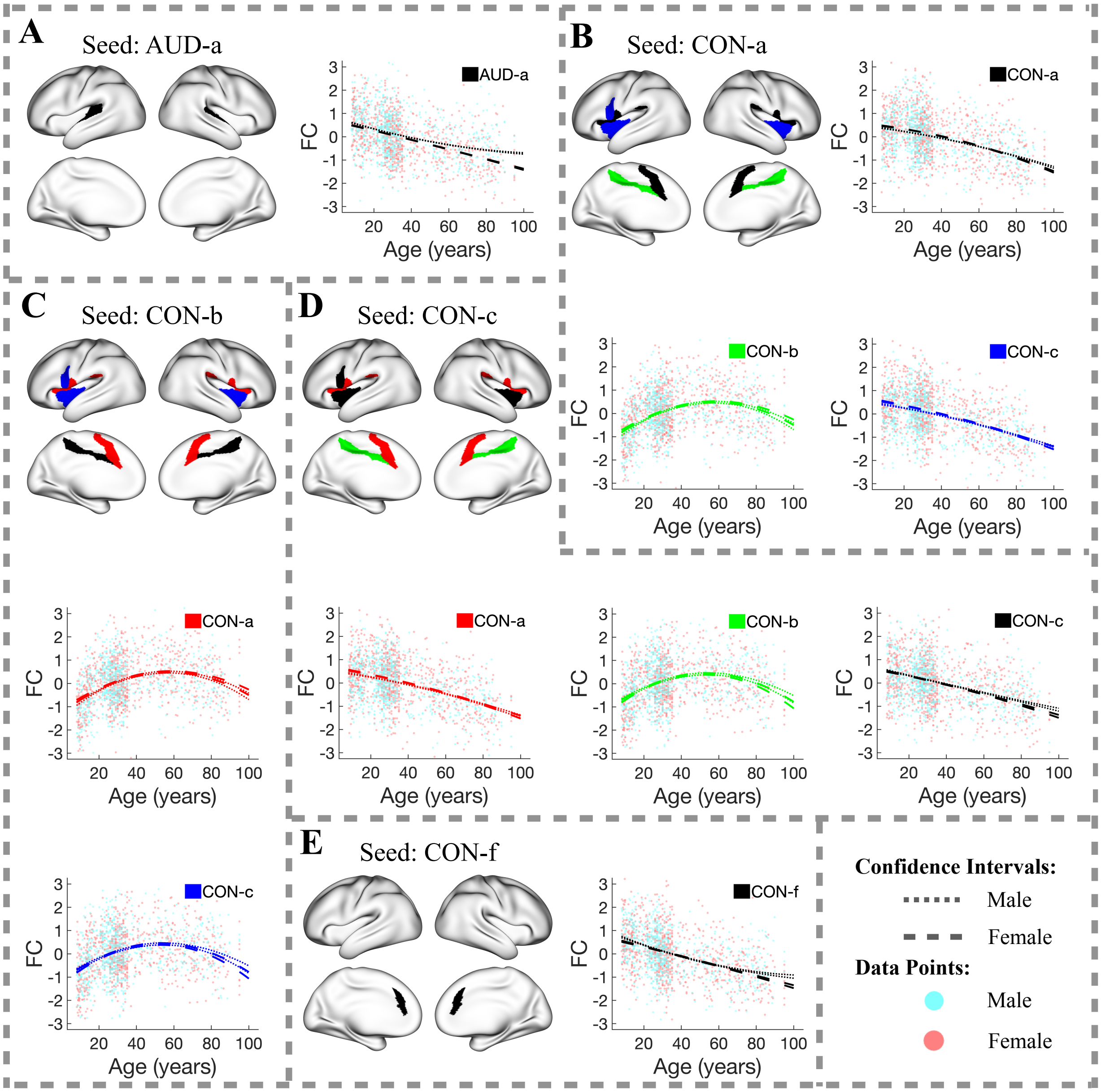

Figure S2: Within-AUD and within-CON FC trajectories with R-squared values exceeding 10%. In each brain plot, one region cluster is designated as the seed cluster, plotted in black. The FC trajectory between the seed cluster and a cluster from the same functional network, say cluster B, is plotted in the same color assigned to cluster B. Light cyan and light coral dots in the FC plots represent FC values between a region in the seed cluster and another region in cluster B for individual male and female subjects, respectively. Dotted lines represent 95% confidence intervals for population-mean FC trajectories of males, whereas dashed lines represent those of females.

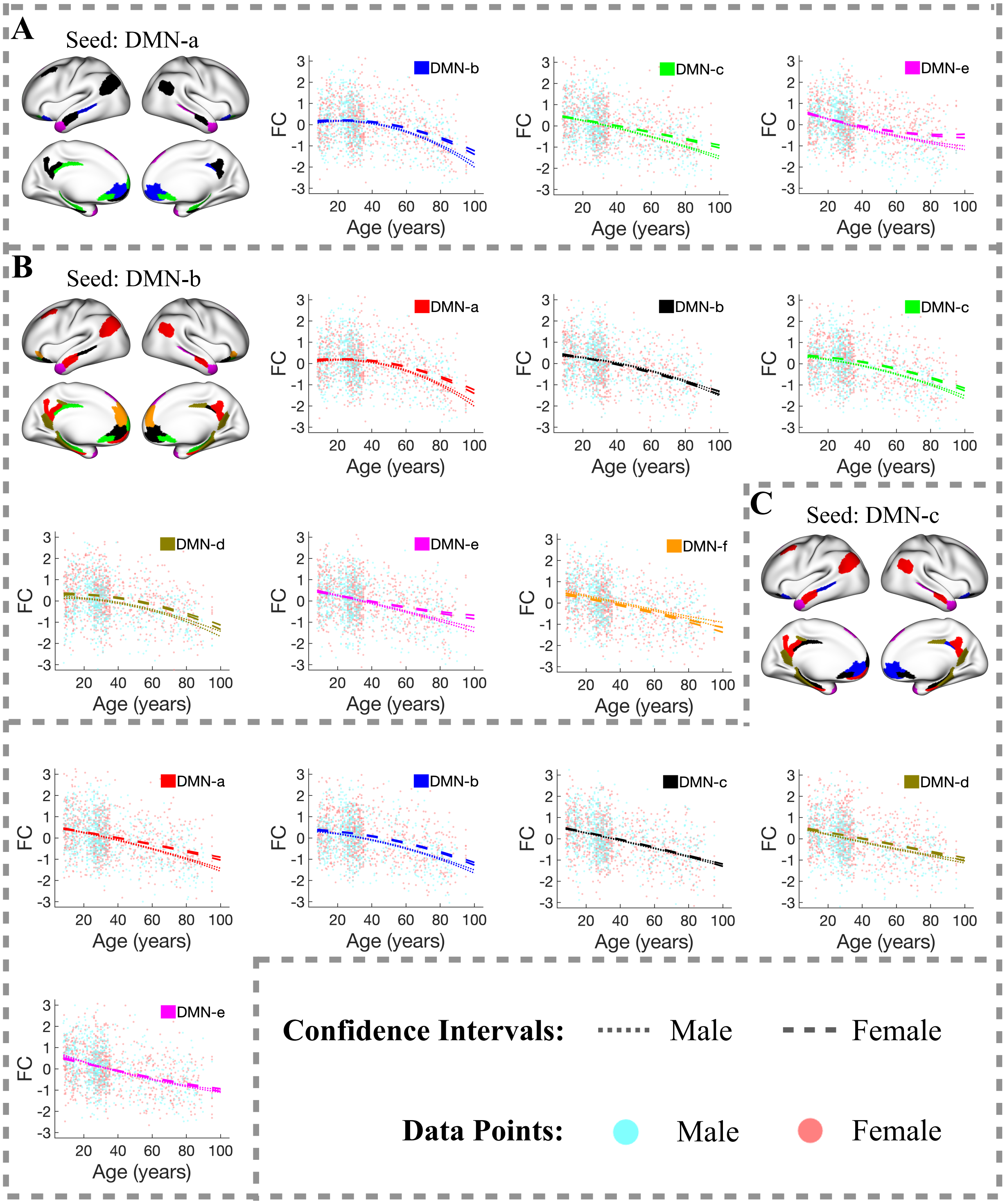

Figure S3: Within-DMN FC trajectories with R-squared values exceeding 10%. In each brain plot, one region cluster is designated as the seed cluster, plotted in black. The FC trajectory between the seed cluster and a cluster from the same functional network, referred to as cluster B, is displayed in the color assigned to cluster B. Light cyan and light coral dots in the FC plots represent FC values between a region in the seed cluster and another region in cluster B for individual male and female subjects, respectively. Dotted lines represent 95% confidence intervals for population-mean FC trajectories of males, whereas dashed lines represent those of females.

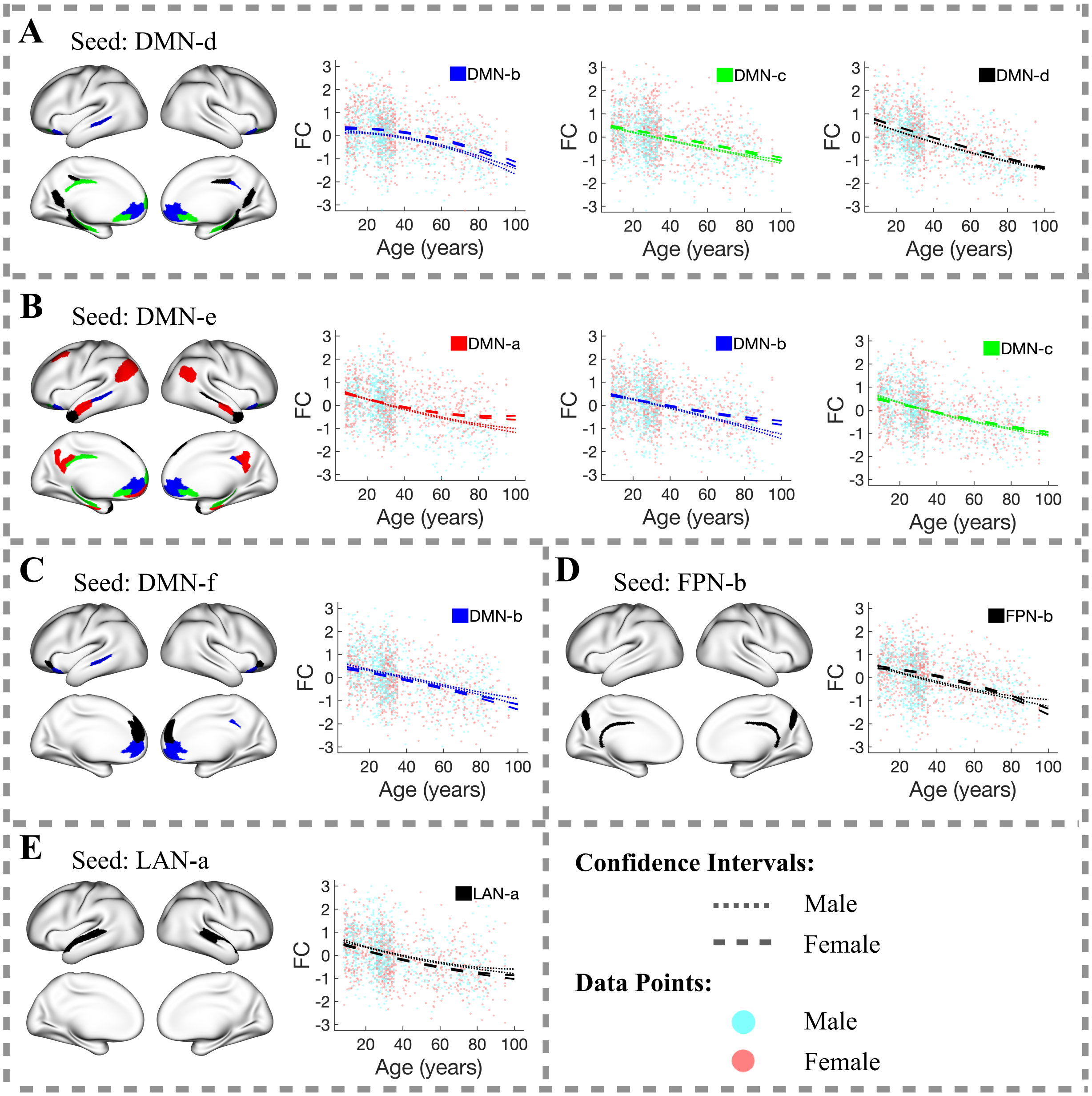

Figure S4: Within-DMN, within-FPN, and within-LAN FC trajectories with R-squared values exceeding 10%. In each brain plot, one region cluster is designated as the seed cluster, plotted in black. The FC trajectory between the seed cluster and a cluster from the same functional network, referred to as cluster B, is displayed in the color assigned to cluster B. Light cyan and light coral dots in the FC plots represent FC values between a region in the seed cluster and another region in cluster B for individual male and female subjects, respectively. Dotted lines represent 95% confidence intervals for population-mean FC trajectories of males, whereas dashed lines represent those of females.

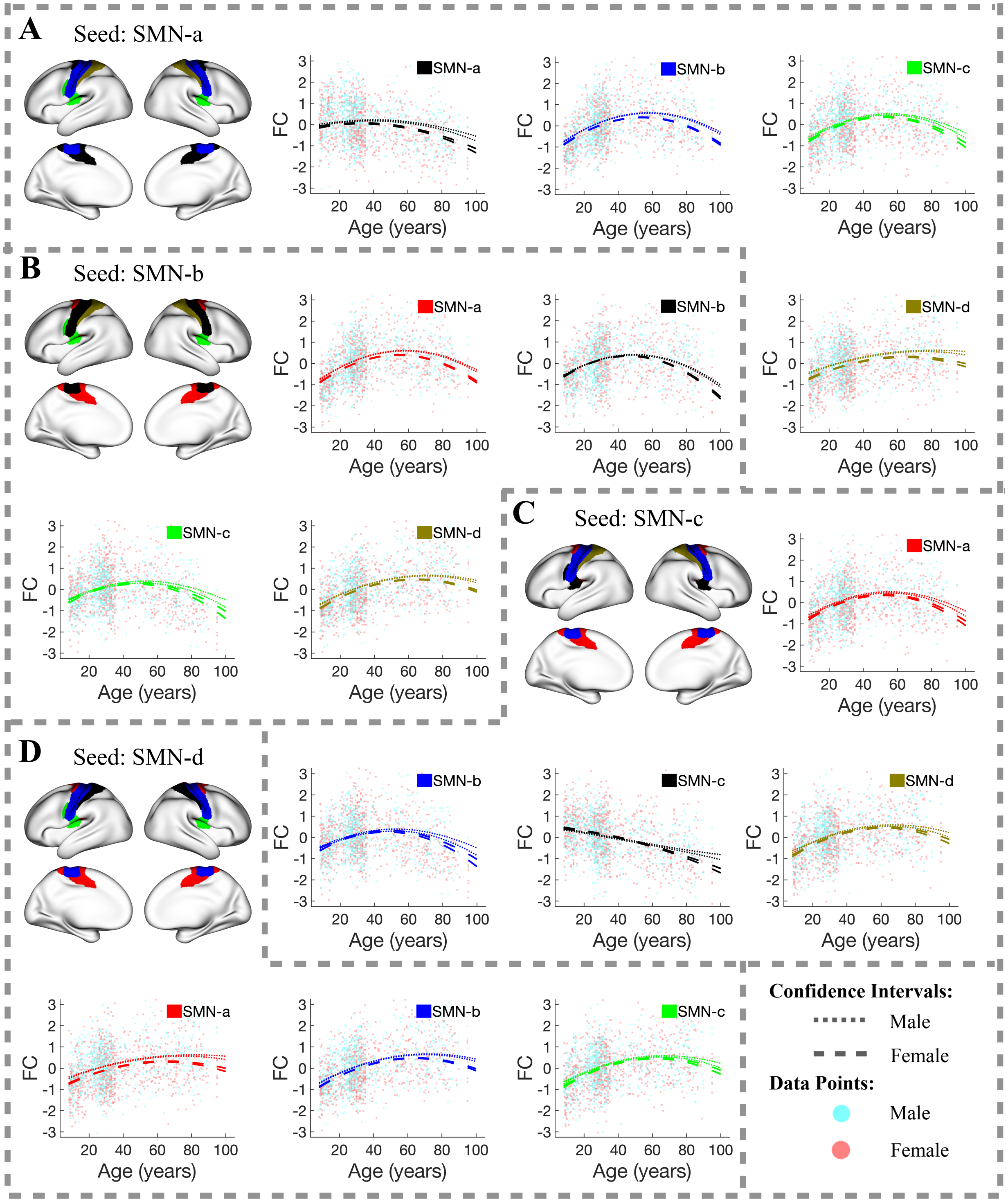

Figure S5: Within-SMN FC trajectories with R-squared values exceeding 10%. In each brain plot, one region cluster is designated as the seed cluster, plotted in black. The FC trajectory between the seed cluster and a cluster from the same functional network, referred to as cluster B, is displayed in the color assigned to cluster B. Light cyan and light coral dots in the FC plots represent FC values between a region in the seed cluster and another region in cluster B for individual male and female subjects, respectively. Dotted lines represent 95% confidence intervals for population-mean FC trajectories of males, whereas dashed lines represent those of females.

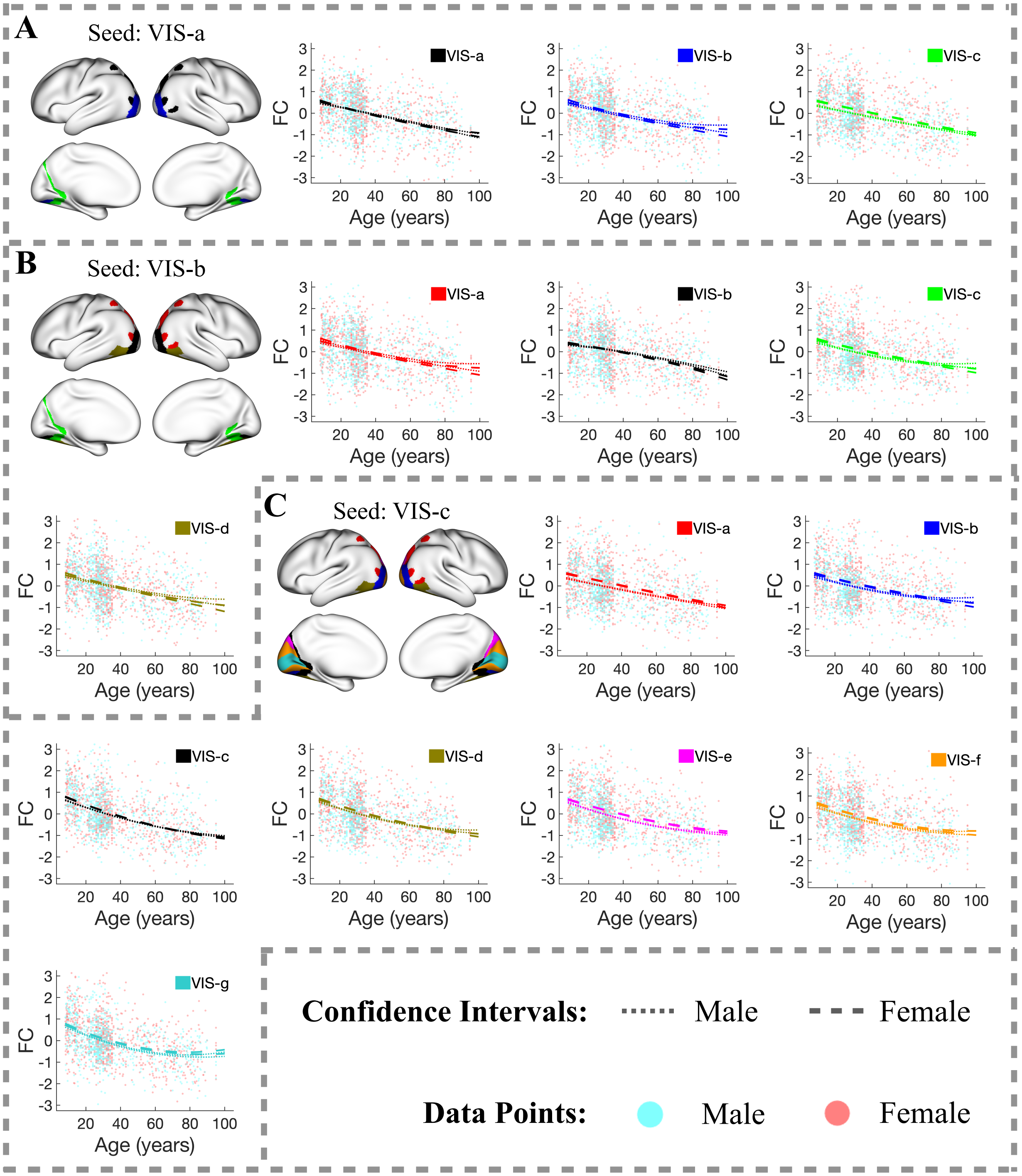

Figure S6: Within-VIS FC trajectories with R-squared values exceeding 10%. In each brain plot, one region cluster is designated as the seed cluster, plotted in black. The FC trajectory between the seed cluster and a cluster from the same functional network, referred to as cluster B, is displayed in the color assigned to cluster B. Light cyan and light coral dots in the FC plots represent FC values between a region in the seed cluster and another region in cluster B for individual male and female subjects, respectively. Dotted lines represent 95% confidence intervals for population-mean FC trajectories of males, whereas dashed lines represent those of females.

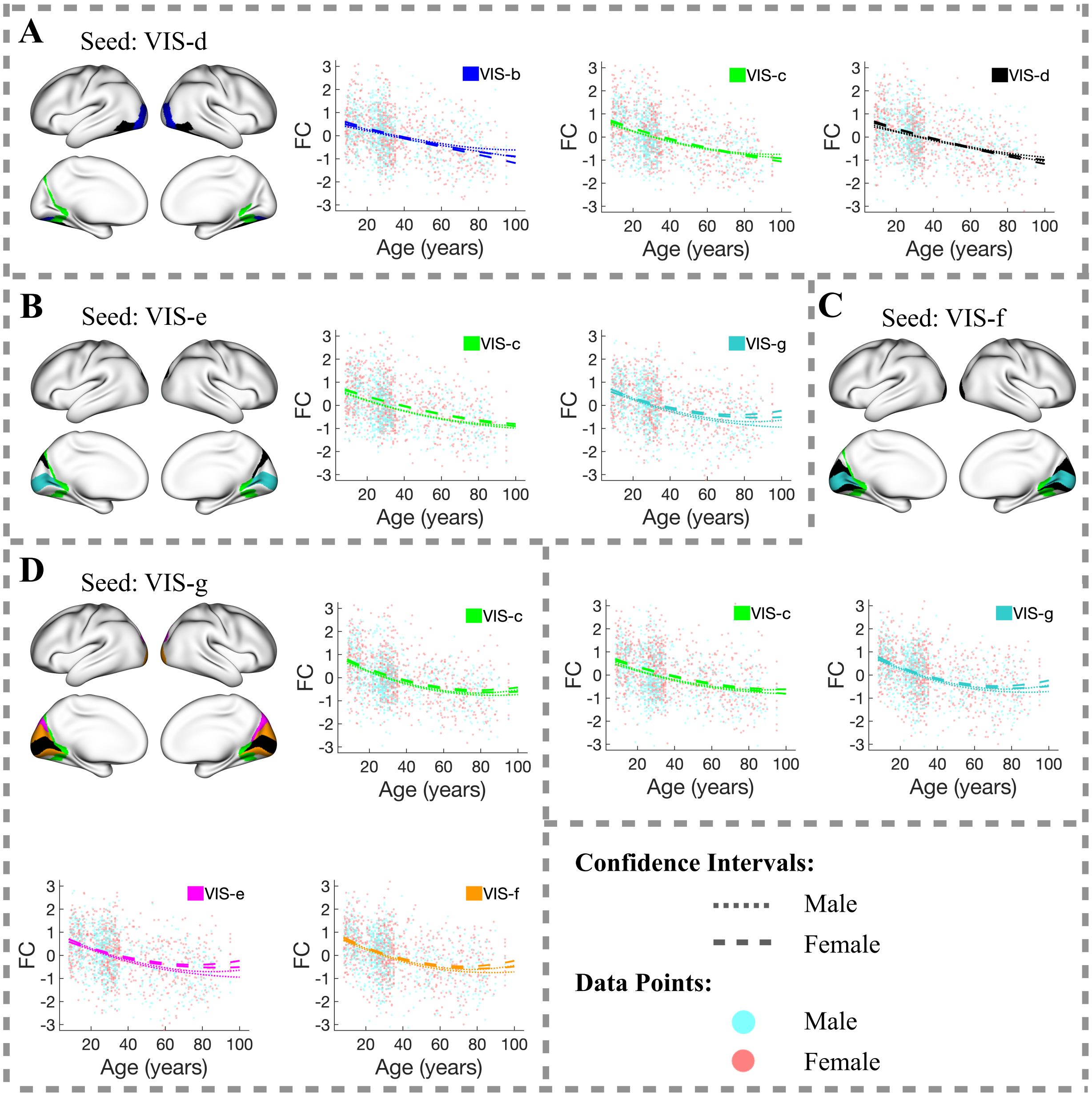

Figure S7: Within-VIS FC trajectories with R-squared values exceeding 10%. In each brain plot, one region cluster is designated as the seed cluster, plotted in black. The FC trajectory between the seed cluster and a cluster from the same functional network, referred to as cluster B, is displayed in the color assigned to cluster B. Light cyan and light coral dots in the FC plots represent FC values between a region in the seed cluster and another region in cluster B for individual male and female subjects, respectively. Dotted lines represent 95% confidence intervals for population-mean FC trajectories of males, whereas dashed lines represent those of females.

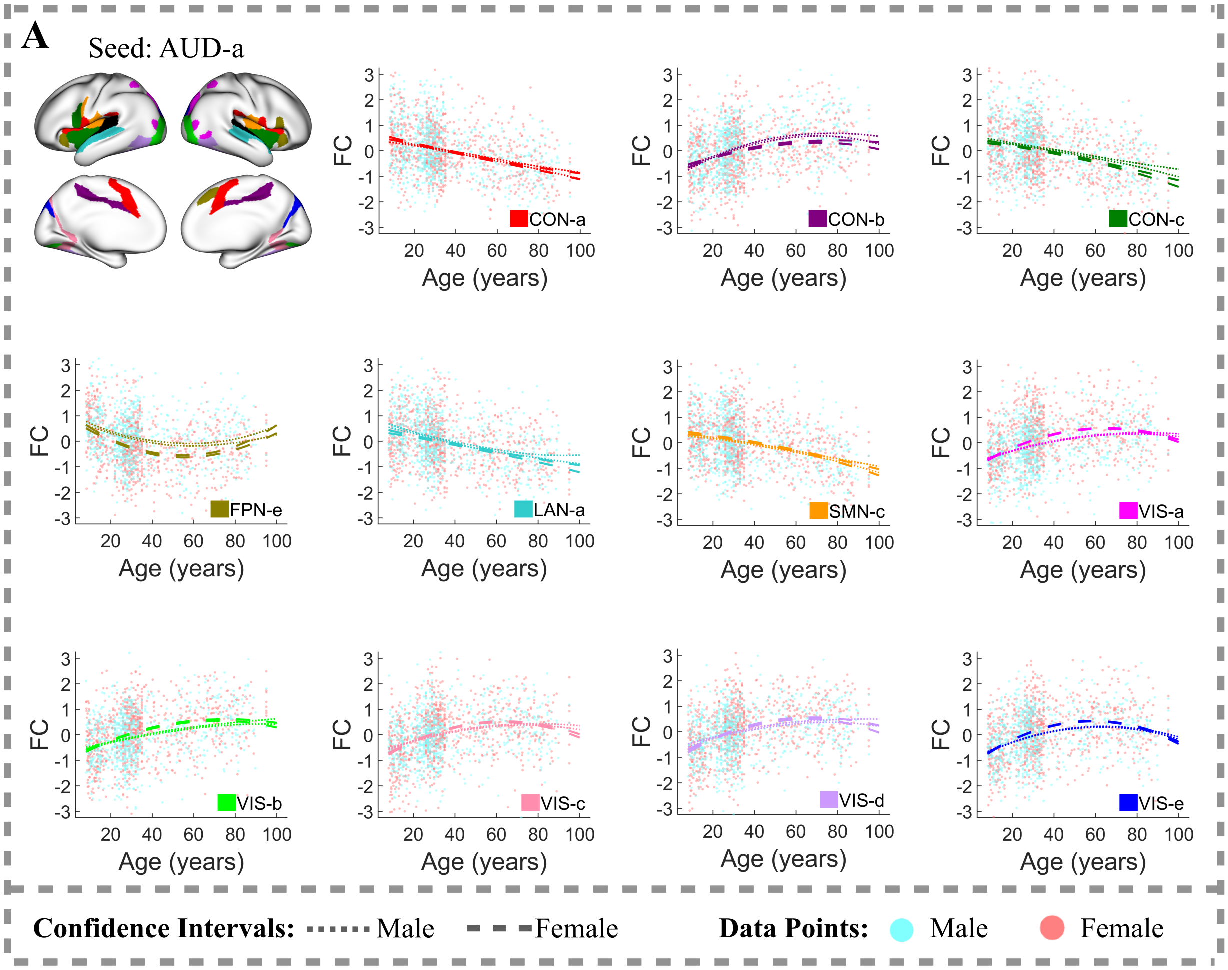

Figure S8: Between-network FC trajectories of the AUD with R-squared values exceeding 10%. In each brain plot, one region cluster is designated as the seed cluster, plotted in black. The FC trajectory between the seed cluster and a cluster from a different functional network, referred to as cluster B, is displayed in the color assigned to cluster B. Light cyan and light coral dots in the FC plots represent FC values between a region in the seed cluster and another region in cluster B for individual male and female subjects, respectively. Dotted lines represent 95% confidence intervals for population-mean FC trajectories of males, whereas dashed lines represent those of females.

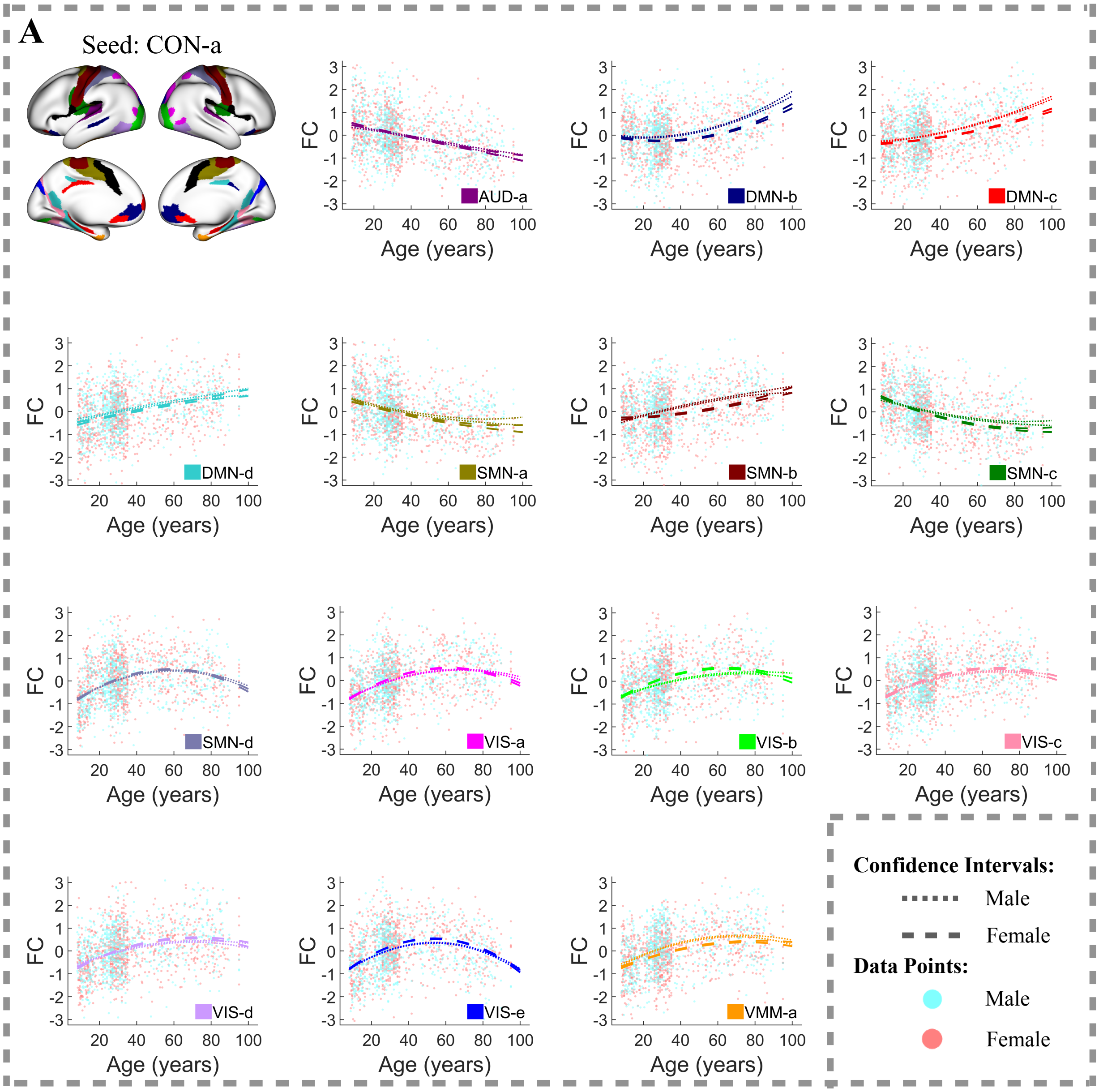

Figure S9: Between-network FC trajectories of the CON with R-squared values exceeding 10%. In each brain plot, one region cluster is designated as the seed cluster, plotted in black. The FC trajectory between the seed cluster and a cluster from a different functional network, referred to as cluster B, is displayed in the color assigned to cluster B. Light cyan and light coral dots in the FC plots represent FC values between a region in the seed cluster and another region in cluster B for individual male and female subjects, respectively. Dotted lines represent 95% confidence intervals for population-mean FC trajectories of males, whereas dashed lines represent those of females.

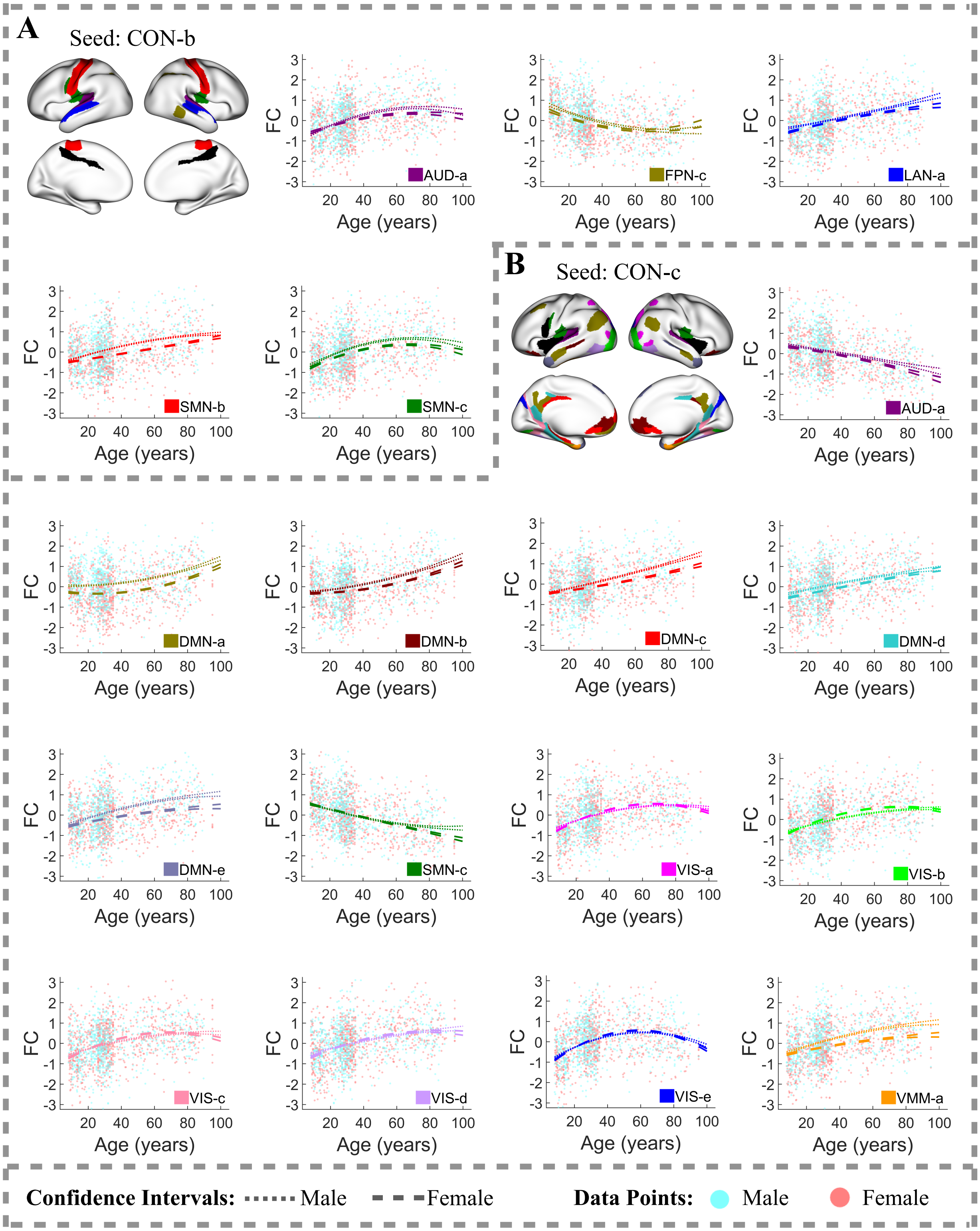

Figure S10: Between-network FC trajectories of the CON with R-squared values exceeding 10%. In each brain plot, one region cluster is designated as the seed cluster, plotted in black. The FC trajectory between the seed cluster and a cluster from a different functional network, referred to as cluster B, is displayed in the color assigned to cluster B. Light cyan and light coral dots in the FC plots represent FC values between a region in the seed cluster and another region in cluster B for individual male and female subjects, respectively. Dotted lines represent 95% confidence intervals for population-mean FC trajectories of males, whereas dashed lines represent those of females.

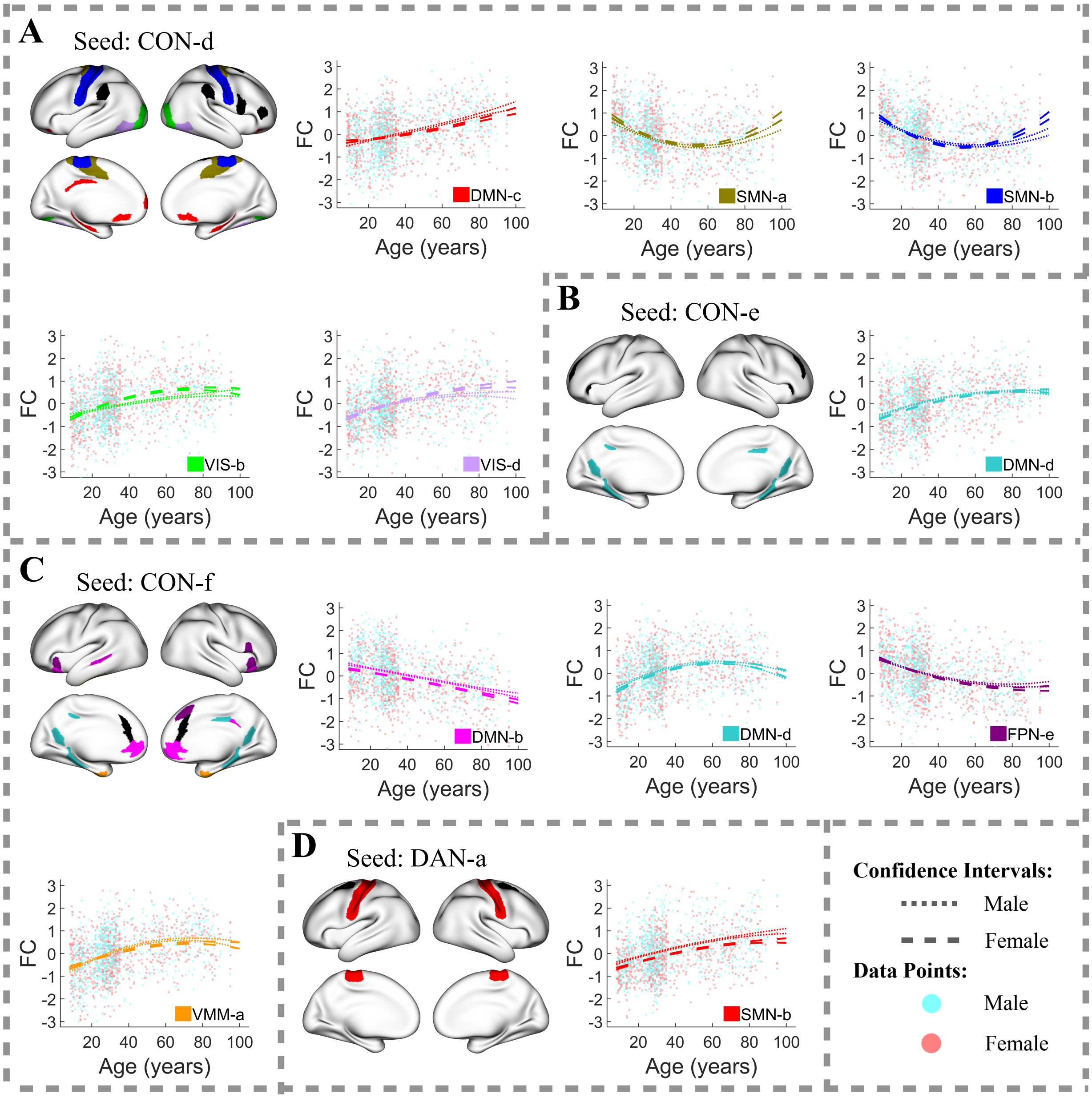

Figure S11: Between-network FC trajectories of the CON and DAN with R-squared values exceeding 10%. In each brain plot, one region cluster is designated as the seed cluster, plotted in black. The FC trajectory between the seed cluster and a cluster from a different functional network, referred to as cluster B, is displayed in the color assigned to cluster B. Light cyan and light coral dots in the FC plots represent FC values between a region in the seed cluster and another region in cluster B for individual male and female subjects, respectively. Dotted lines represent 95% confidence intervals for population-mean FC trajectories of males, whereas dashed lines represent those of females.

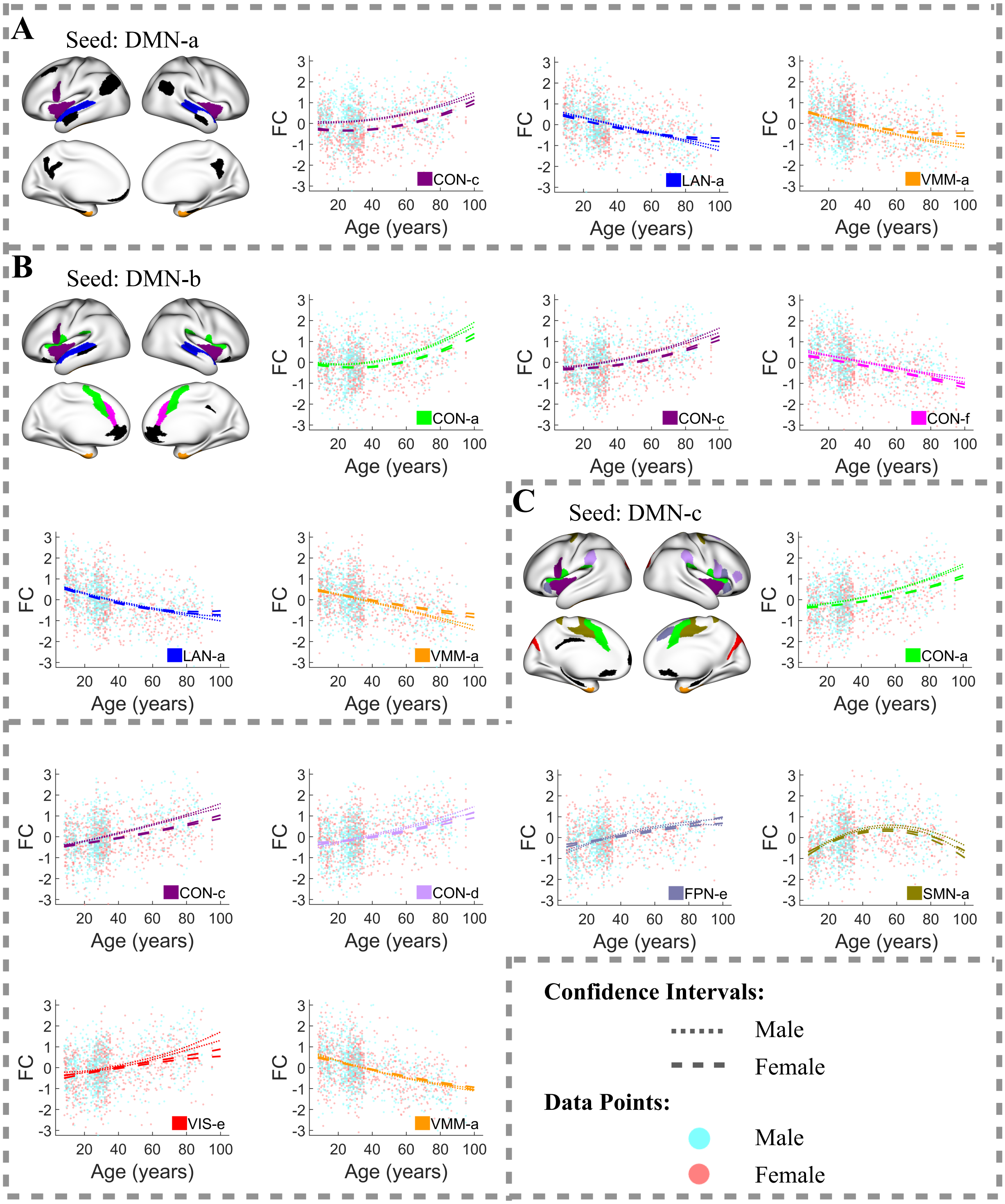

Figure S12: Between-network FC trajectories of the DMN with R-squared values exceeding 10%. In each brain plot, one region cluster is designated as the seed cluster, plotted in black. The FC trajectory between the seed cluster and a cluster from a different functional network, referred to as cluster B, is displayed in the color assigned to cluster B. Light cyan and light coral dots in the FC plots represent FC values between a region in the seed cluster and another region in cluster B for individual male and female subjects, respectively. Dotted lines represent 95% confidence intervals for population-mean FC trajectories of males, whereas dashed lines represent those of females.

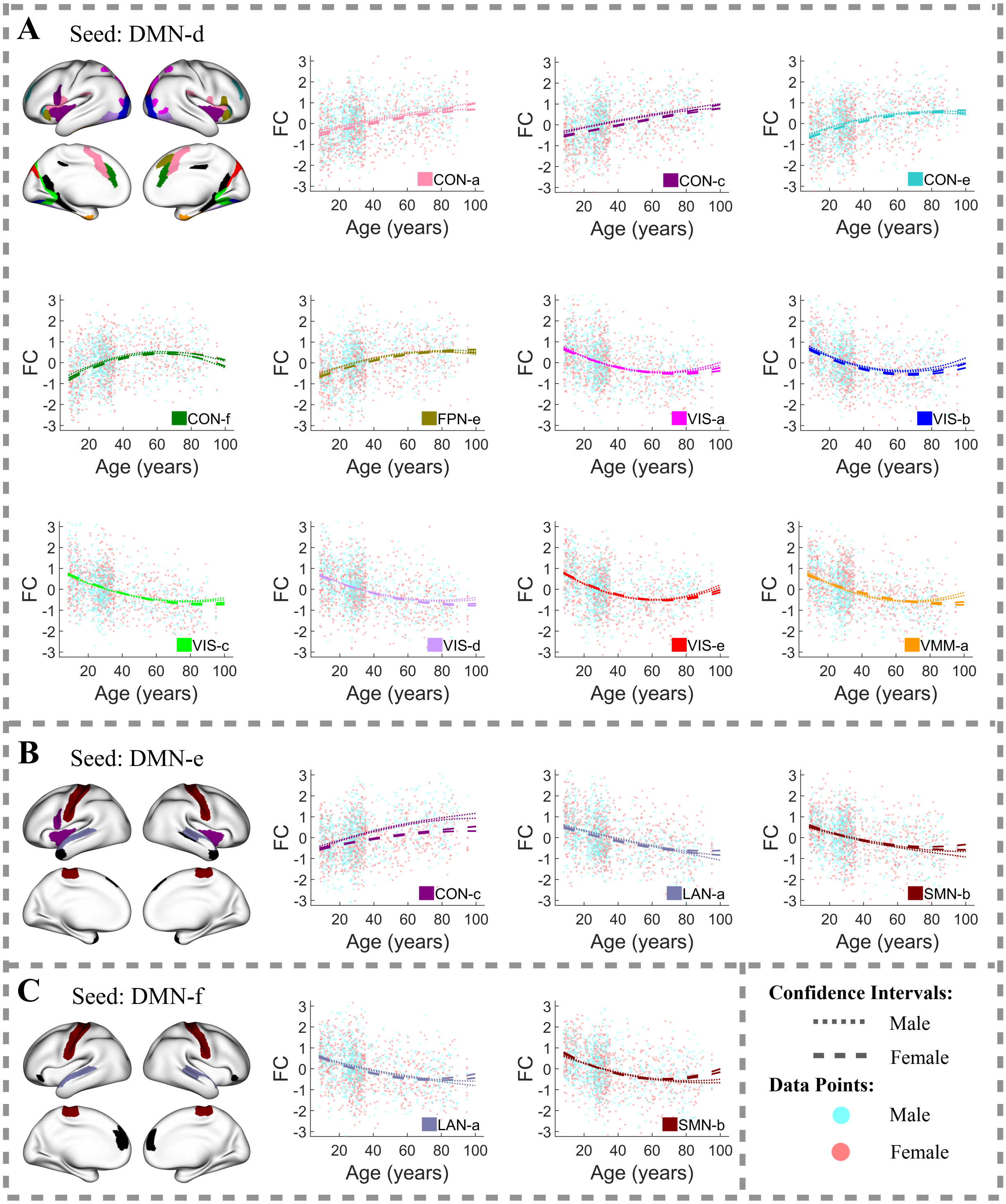

Figure S13: Between-network FC trajectories of the DMN with R-squared values exceeding 10%. In each brain plot, one region cluster is designated as the seed cluster, plotted in black. The FC trajectory between the seed cluster and a cluster from a different functional network, referred to as cluster B, is displayed in the color assigned to cluster B. Light cyan and light coral dots in the FC plots represent FC values between a region in the seed cluster and another region in cluster B for individual male and female subjects, respectively. Dotted lines represent 95% confidence intervals for population-mean FC trajectories of males, whereas dashed lines represent those of females.

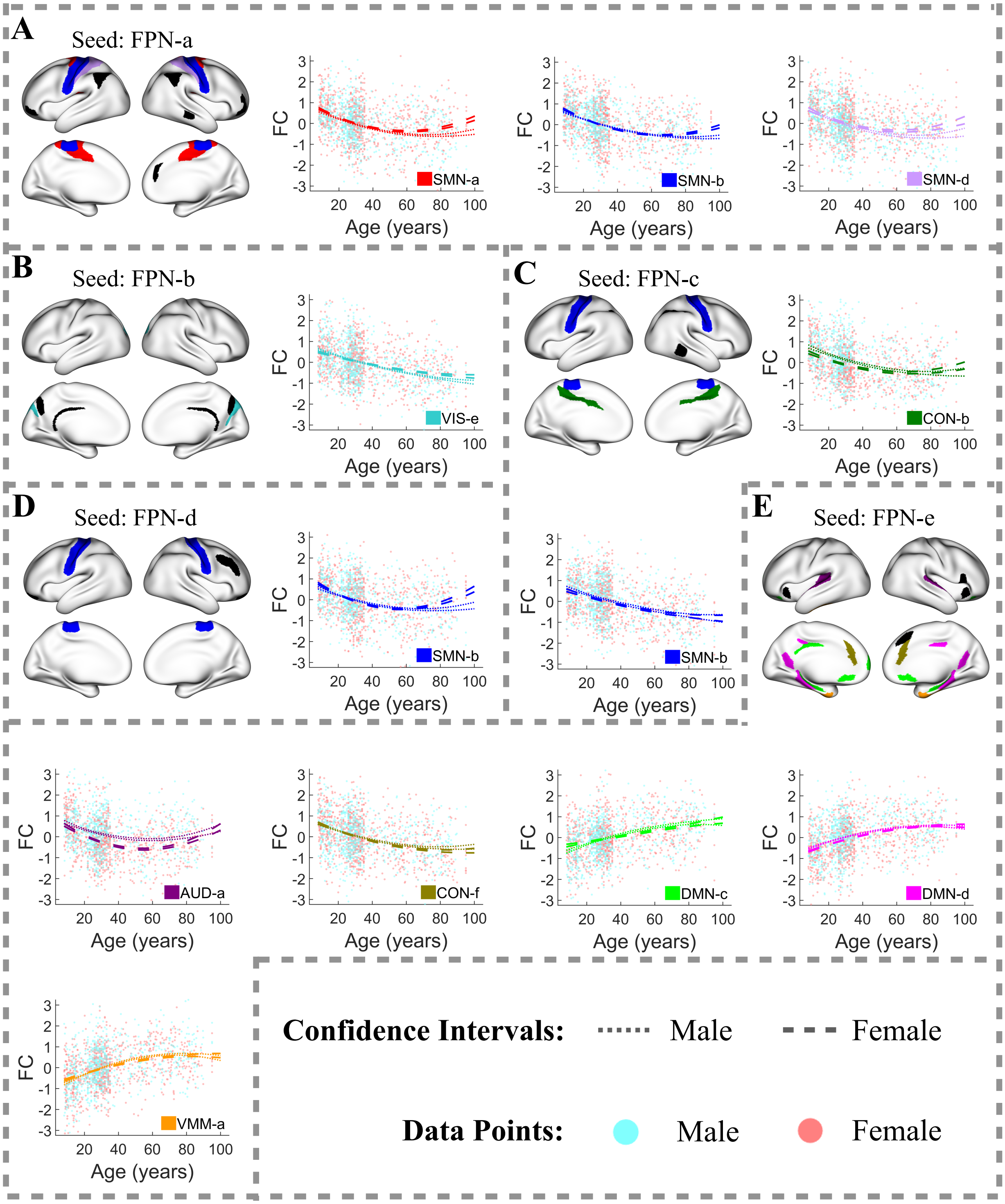

Figure S14: Between-network FC trajectories of the FPN with R-squared values exceeding 10%. In each brain plot, one region cluster is designated as the seed cluster, plotted in black. The FC trajectory between the seed cluster and a cluster from a different functional network, referred to as cluster B, is displayed in the color assigned to cluster B. Light cyan and light coral dots in the FC plots represent FC values between a region in the seed cluster and another region in cluster B for individual male and female subjects, respectively. Dotted lines represent 95% confidence intervals for population-mean FC trajectories of males, whereas dashed lines represent those of females.

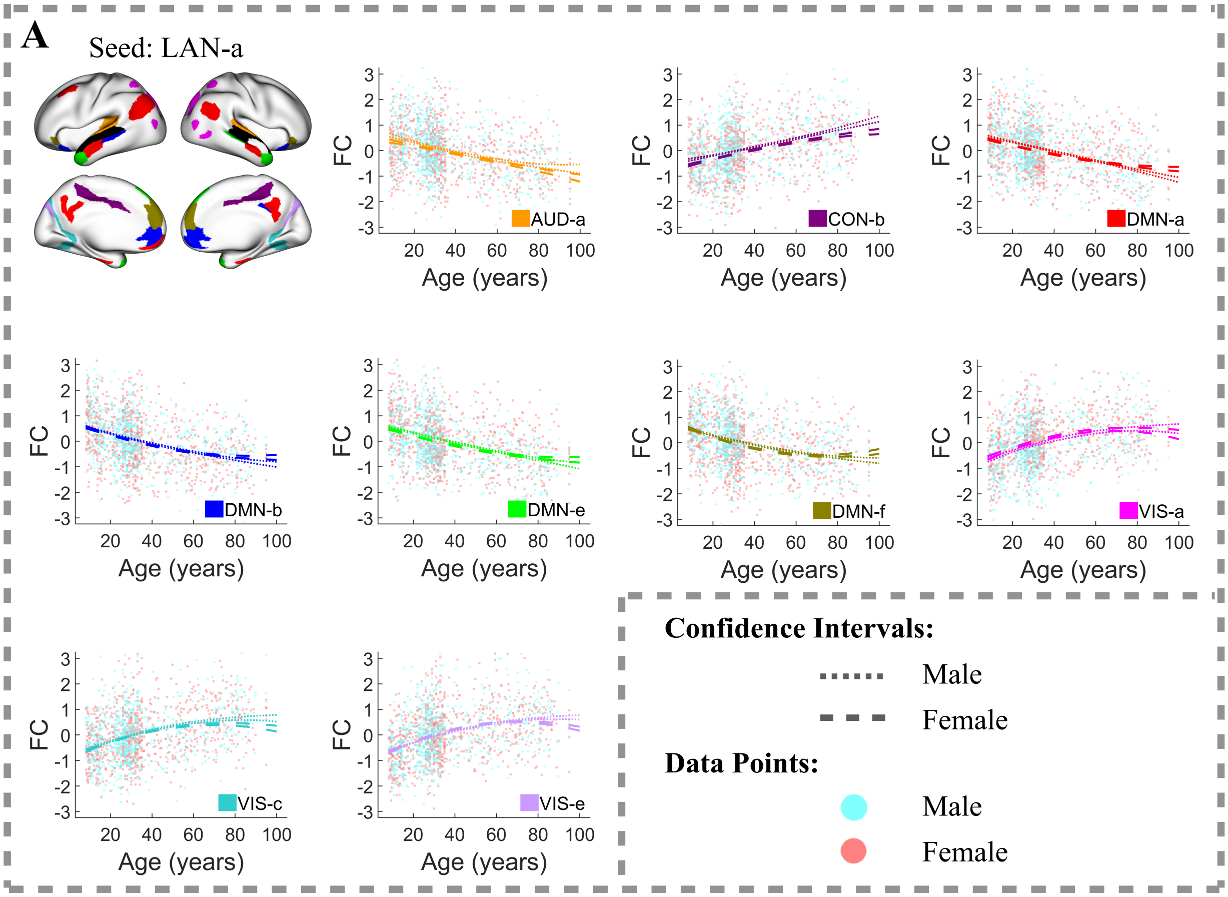

Figure S15: Between-network FC trajectories of the LAN with R-squared values exceeding 10%. In each brain plot, one region cluster is designated as the seed cluster, plotted in black. The FC trajectory between the seed cluster and a cluster from a different functional network, referred to as cluster B, is displayed in the color assigned to cluster B. Light cyan and light coral dots in the FC plots represent FC values between a region in the seed cluster and another region in cluster B for individual male and female subjects, respectively. Dotted lines represent 95% confidence intervals for population-mean FC trajectories of males, whereas dashed lines represent those of females.

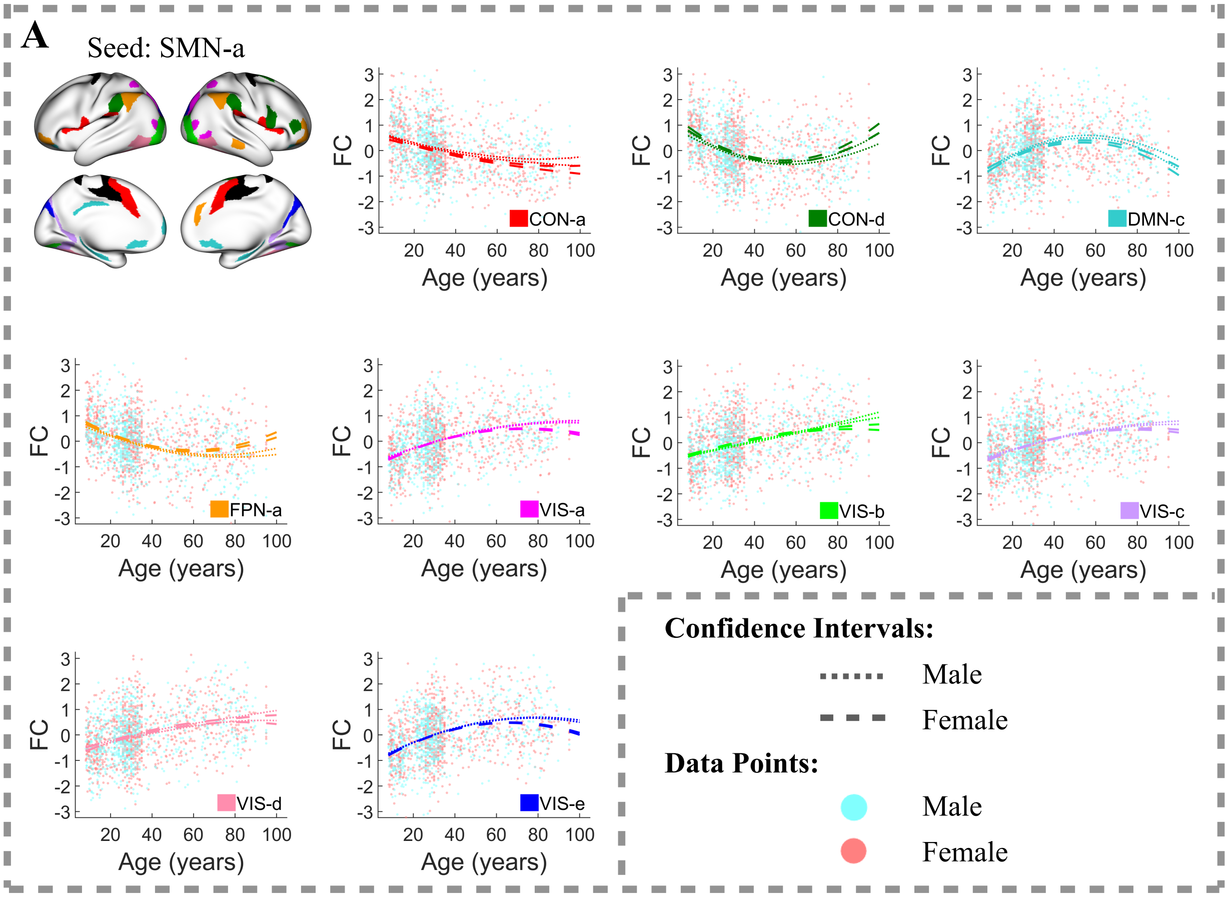

Figure S16: Between-network FC trajectories of the SMN with R-squared values exceeding 10%. In each brain plot, one region cluster is designated as the seed cluster, plotted in black. The FC trajectory between the seed cluster and a cluster from a different functional network, referred to as cluster B, is displayed in the color assigned to cluster B. Light cyan and light coral dots in the FC plots represent FC values between a region in the seed cluster and another region in cluster B for individual male and female subjects, respectively. Dotted lines represent 95% confidence intervals for population-mean FC trajectories of males, whereas dashed lines represent those of females.

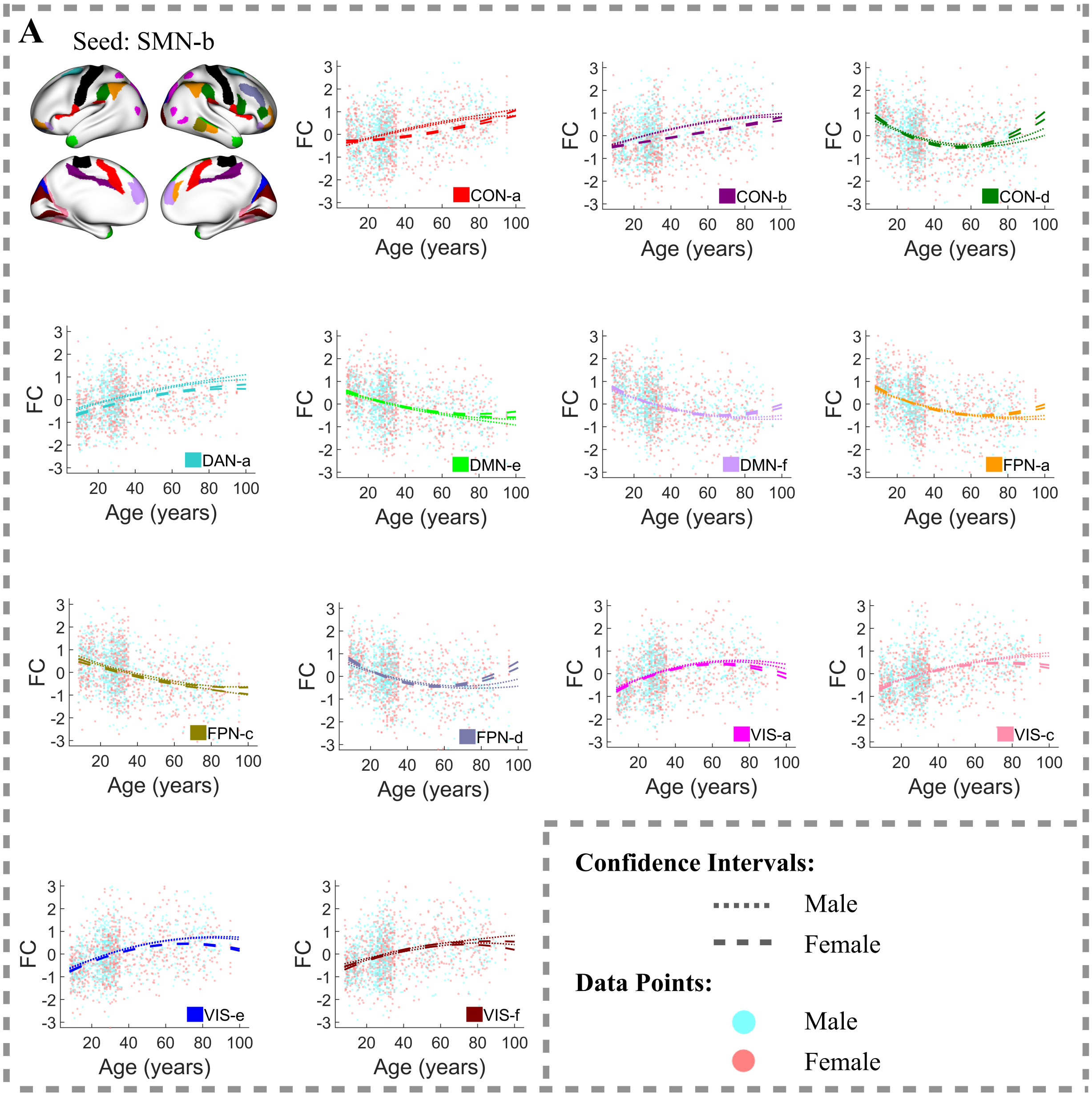

Figure S17: Between-network FC trajectories of the SMN with R-squared values exceeding 10%. In each brain plot, one region cluster is designated as the seed cluster, plotted in black. The FC trajectory between the seed cluster and a cluster from a different functional network, referred to as cluster B, is displayed in the color assigned to cluster B. Light cyan and light coral dots in the FC plots represent FC values between a region in the seed cluster and another region in cluster B for individual male and female subjects, respectively. Dotted lines represent 95% confidence intervals for population-mean FC trajectories of males, whereas dashed lines represent those of females.

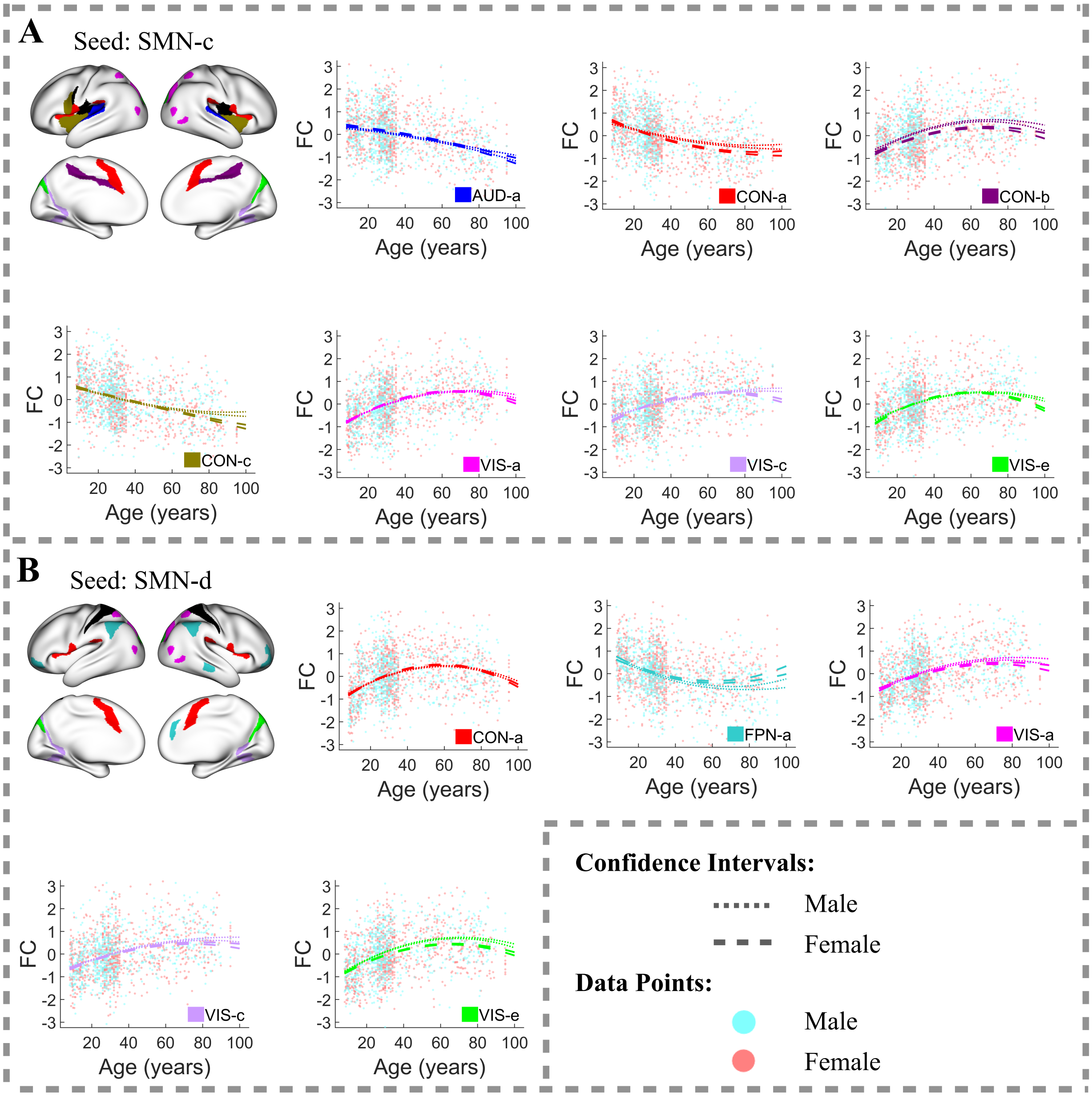

Figure S18: Between-network FC trajectories of the SMN with R-squared values exceeding 10%. In each brain plot, one region cluster is designated as the seed cluster, plotted in black. The FC trajectory between the seed cluster and a cluster from a different functional network, referred to as cluster B, is displayed in the color assigned to cluster B. Light cyan and light coral dots in the FC plots represent FC values between a region in the seed cluster and another region in cluster B for individual male and female subjects, respectively. Dotted lines represent 95% confidence intervals for population-mean FC trajectories of males, whereas dashed lines represent those of females.

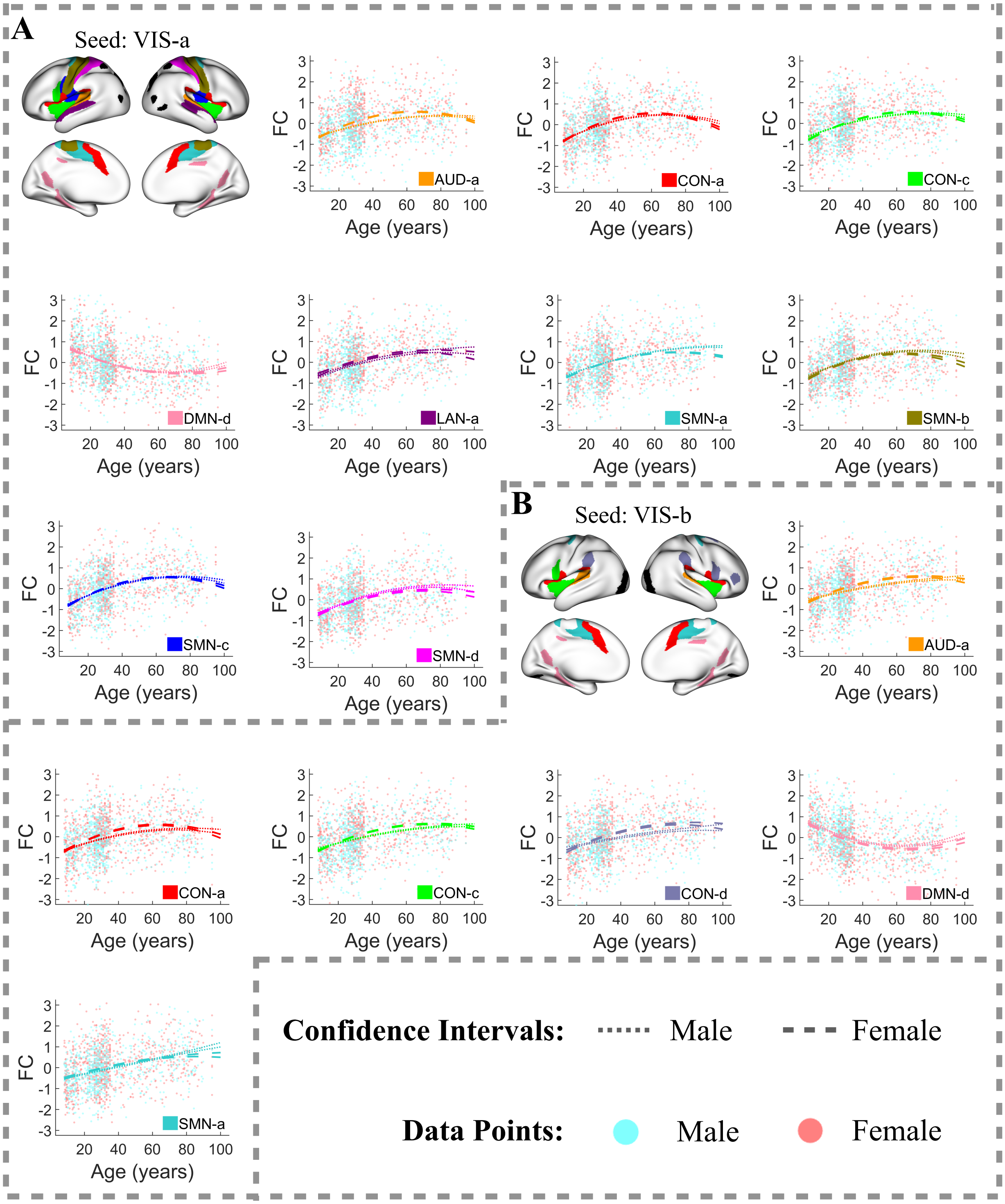

Figure S19: Between-network FC trajectories of the VIS with R-squared values exceeding 10%. In each brain plot, one region cluster is designated as the seed cluster, plotted in black. The FC trajectory between the seed cluster and a cluster from a different functional network, referred to as cluster B, is displayed in the color assigned to cluster B. Light cyan and light coral dots in the FC plots represent FC values between a region in the seed cluster and another region in cluster B for individual male and female subjects, respectively. Dotted lines represent 95% confidence intervals for population-mean FC trajectories of males, whereas dashed lines represent those of females.

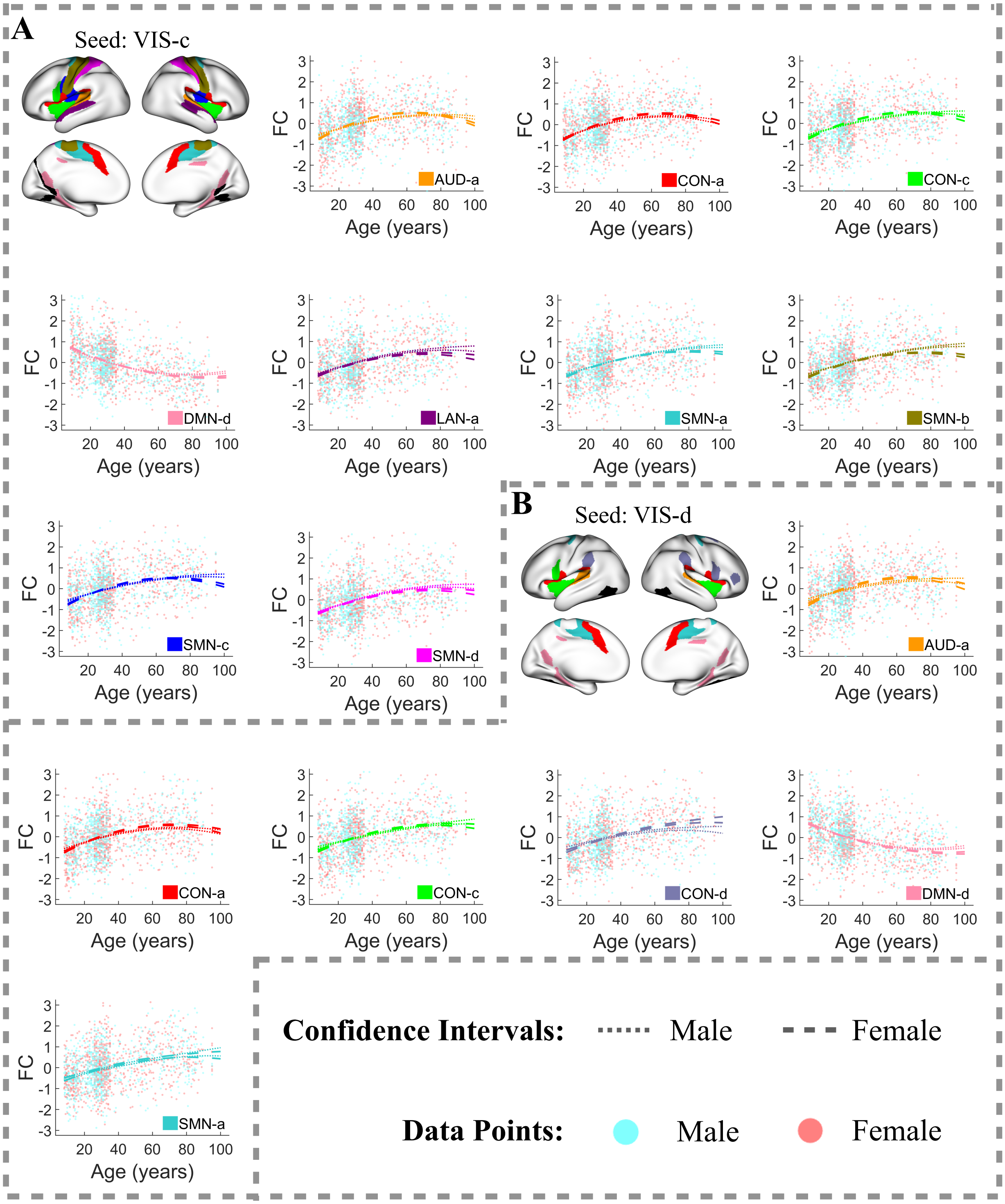

Figure S20: Between-network FC trajectories of the VIS with R-squared values exceeding 10%. In each brain plot, one region cluster is designated as the seed cluster, plotted in black. The FC trajectory between the seed cluster and a cluster from a different functional network, referred to as cluster B, is displayed in the color assigned to cluster B. Light cyan and light coral dots in the FC plots represent FC values between a region in the seed cluster and another region in cluster B for individual male and female subjects, respectively. Dotted lines represent 95% confidence intervals for population-mean FC trajectories of males, whereas dashed lines represent those of females.

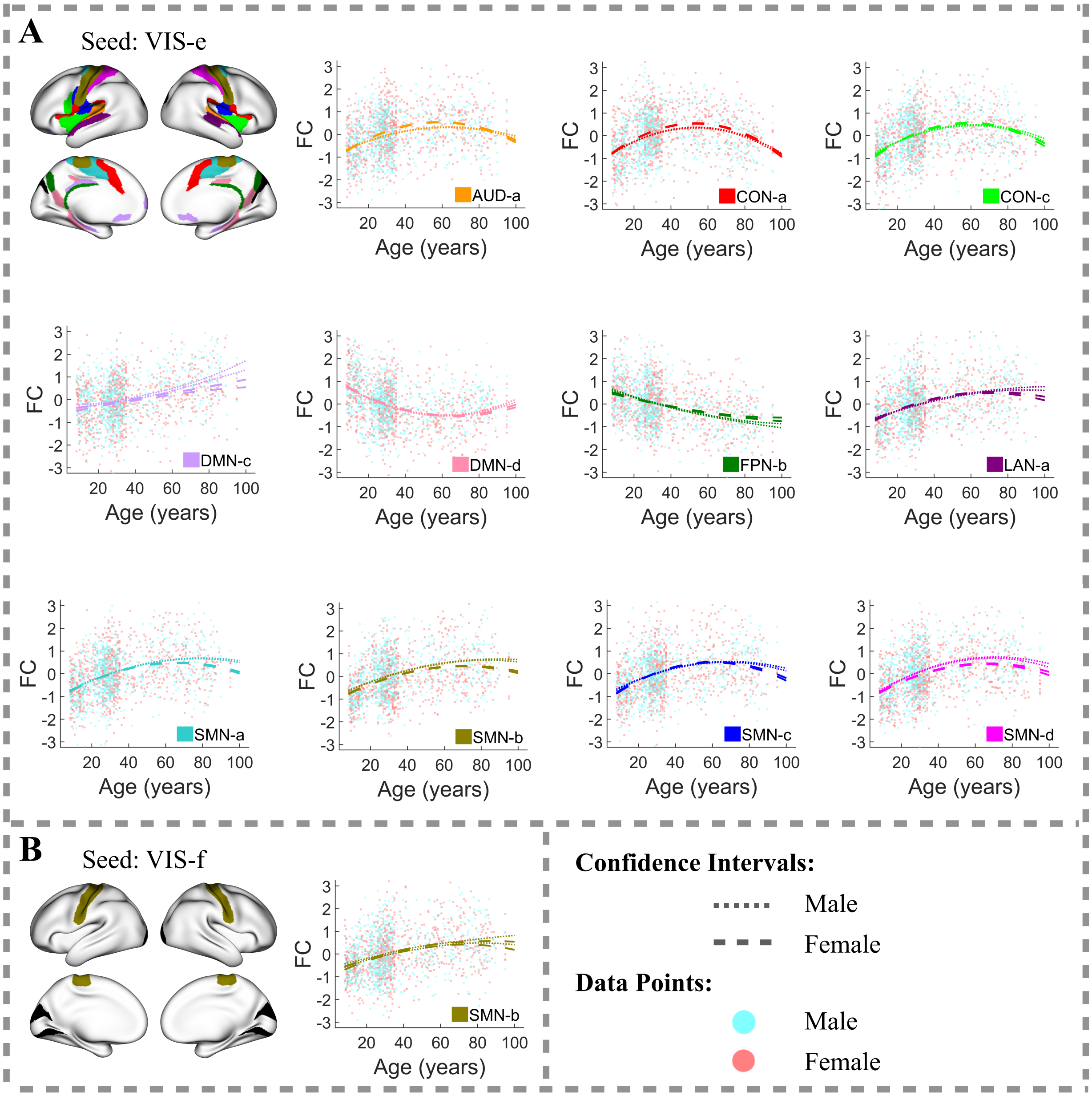

Figure S21: Between-network FC trajectories of the VIS with R-squared values exceeding 10%. In each brain plot, one region cluster is designated as the seed cluster, plotted in black. The FC trajectory between the seed cluster and a cluster from a different functional network, referred to as cluster B, is displayed in the color assigned to cluster B. Light cyan and light coral dots in the FC plots represent FC values between a region in the seed cluster and another region in cluster B for individual male and female subjects, respectively. Dotted lines represent 95% confidence intervals for population-mean FC trajectories of males, whereas dashed lines represent those of females.

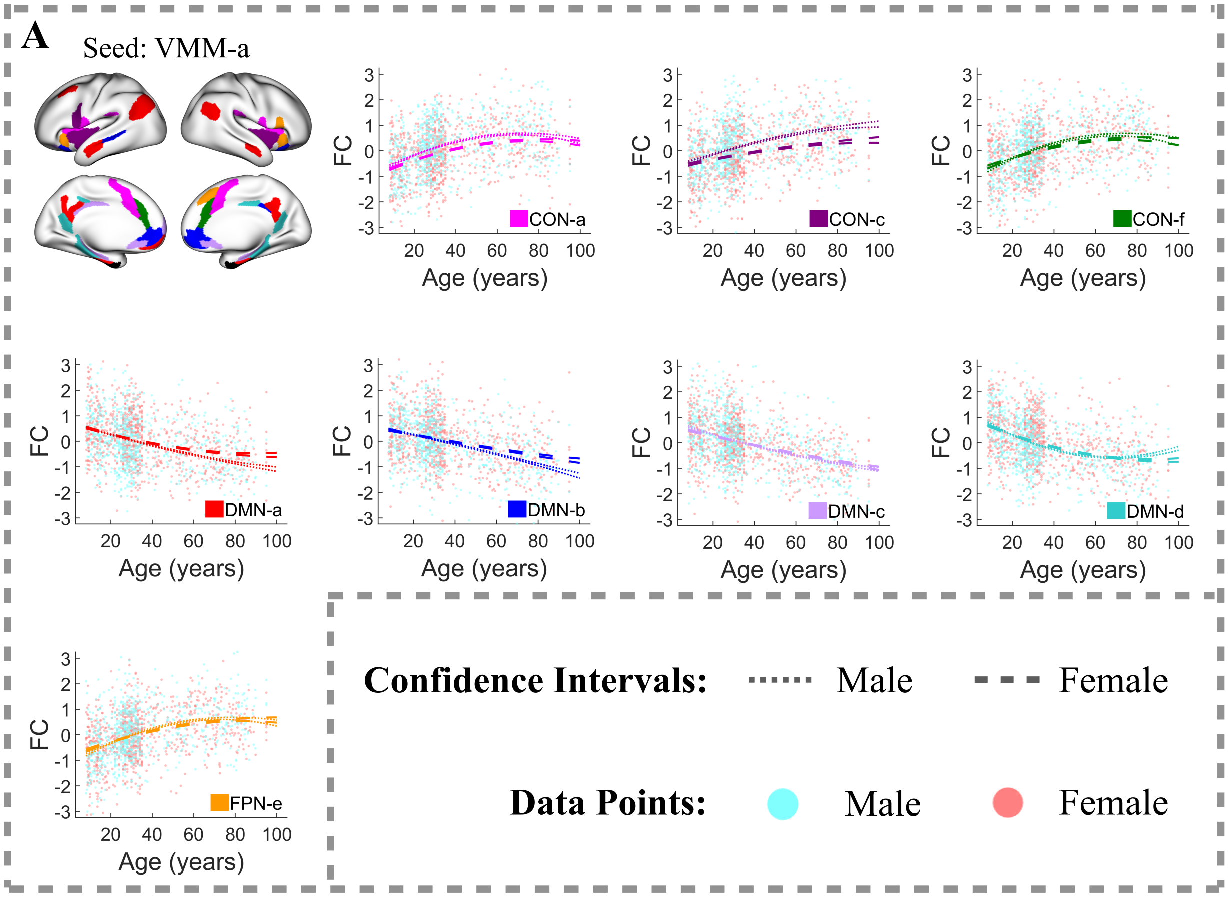

Figure S22: Between-network FC trajectories of the VMM with R-squared values exceeding 10%. In each brain plot, one region cluster is designated as the seed cluster, plotted in black. The FC trajectory between the seed cluster and a cluster from a different functional network, referred to as cluster B, is displayed in the color assigned to cluster B. Light cyan and light coral dots in the FC plots represent FC values between a region in the seed cluster and another region in cluster B for individual male and female subjects, respectively. Dotted lines represent 95% confidence intervals for population-mean FC trajectories of males, whereas dashed lines represent those of females.

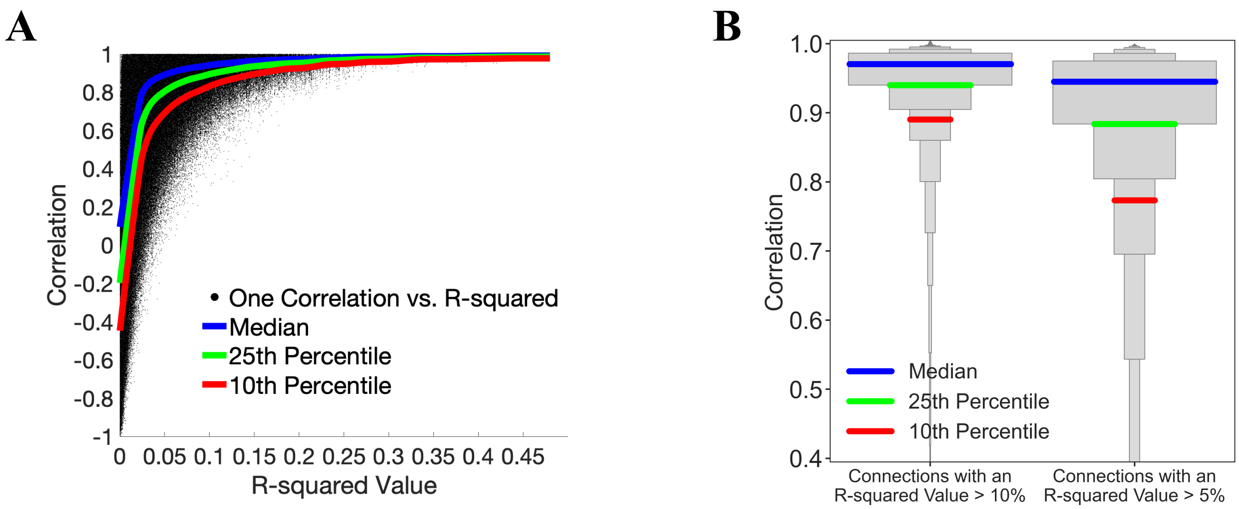

Figure S23: Correlations between FC trajectories estimated in 50 random pairs of non-overlapping subsets of the data. A. Each black dot represents the correlation between estimated FC trajectories derived from independent regression analyses of randomly divided half datasets, plotted against the R-squared value from the same analysis applied to all subjects' data for one connection. The blue, green, and red lines indicate the median, 25th, and 10th percentiles of these correlations, respectively, across various R-squared values. B. Boxplots of correlations between FC trajectories estimated by clustering-enabled regression analysis for connections selected with 10% and 5% R-squared thresholds in 50 pairs of randomly divided half datasets.

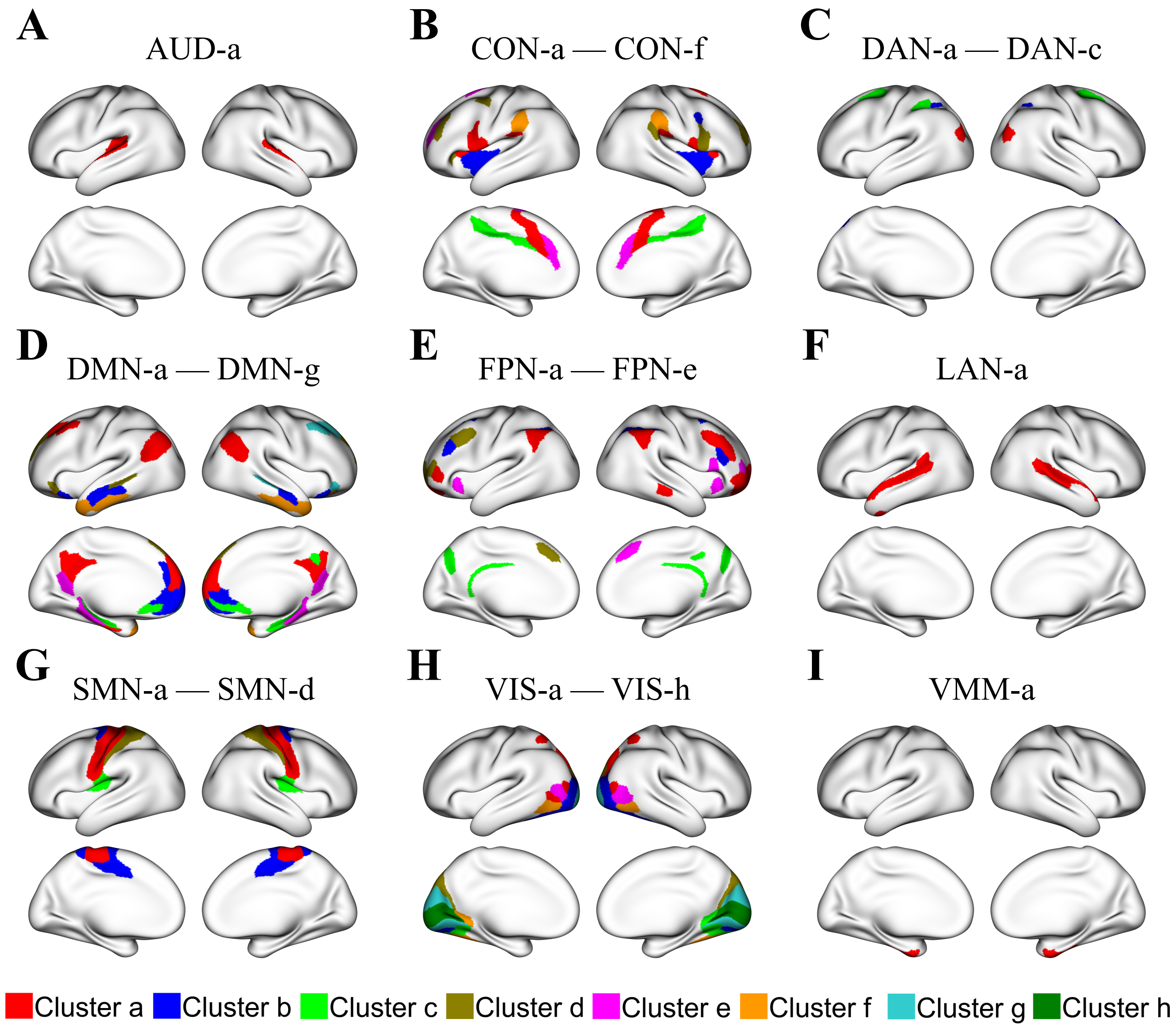

Figure S24: Identified region clusters based on connections with R-squared values exceeding 5%. Each cluster contains at least four regions or 500 grayordinates from the same functional network. Within a single functional network, region clusters are plotted in different colors and labeled (e.g., a, b, etc.) in a descending order based on their region count, from the largest to the smallest. The functional networks include the auditory (AUD), cingulo-opercular (CON), dorsal attention (DAN), default mode (DMN), frontoparietal (FPN), language (LAN), somatomotor (SMN), visual (VIS), and ventral multimodal (VMM) networks.

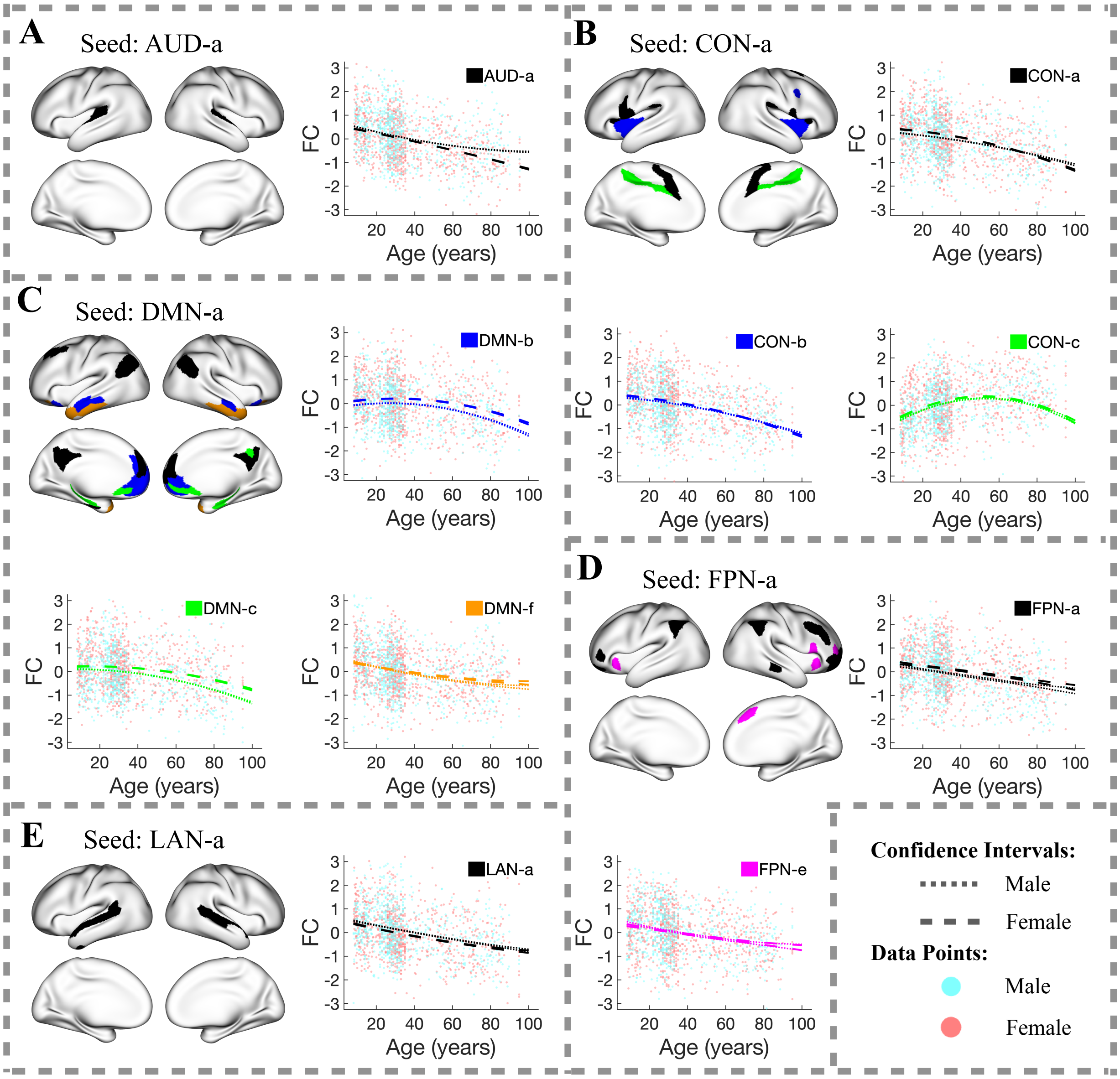

Figure S25: Within-Network FC trajectories of the AUD-a, CON-a, DMN-a, FPN-a, and LAN-a with R-squared values exceeding 5%. In each brain plot, one region cluster is designated as the seed cluster, plotted in black. The FC trajectory between the seed cluster and a cluster from the same functional network, say cluster B, is plotted in the same color assigned to cluster B. Light cyan and light coral dots in the FC plots represent FC values between a region in the seed cluster and another region in cluster B for individual male and female subjects, respectively. Dotted lines represent 95% confidence intervals for population-mean FC trajectories of males, whereas dashed lines represent those of females.

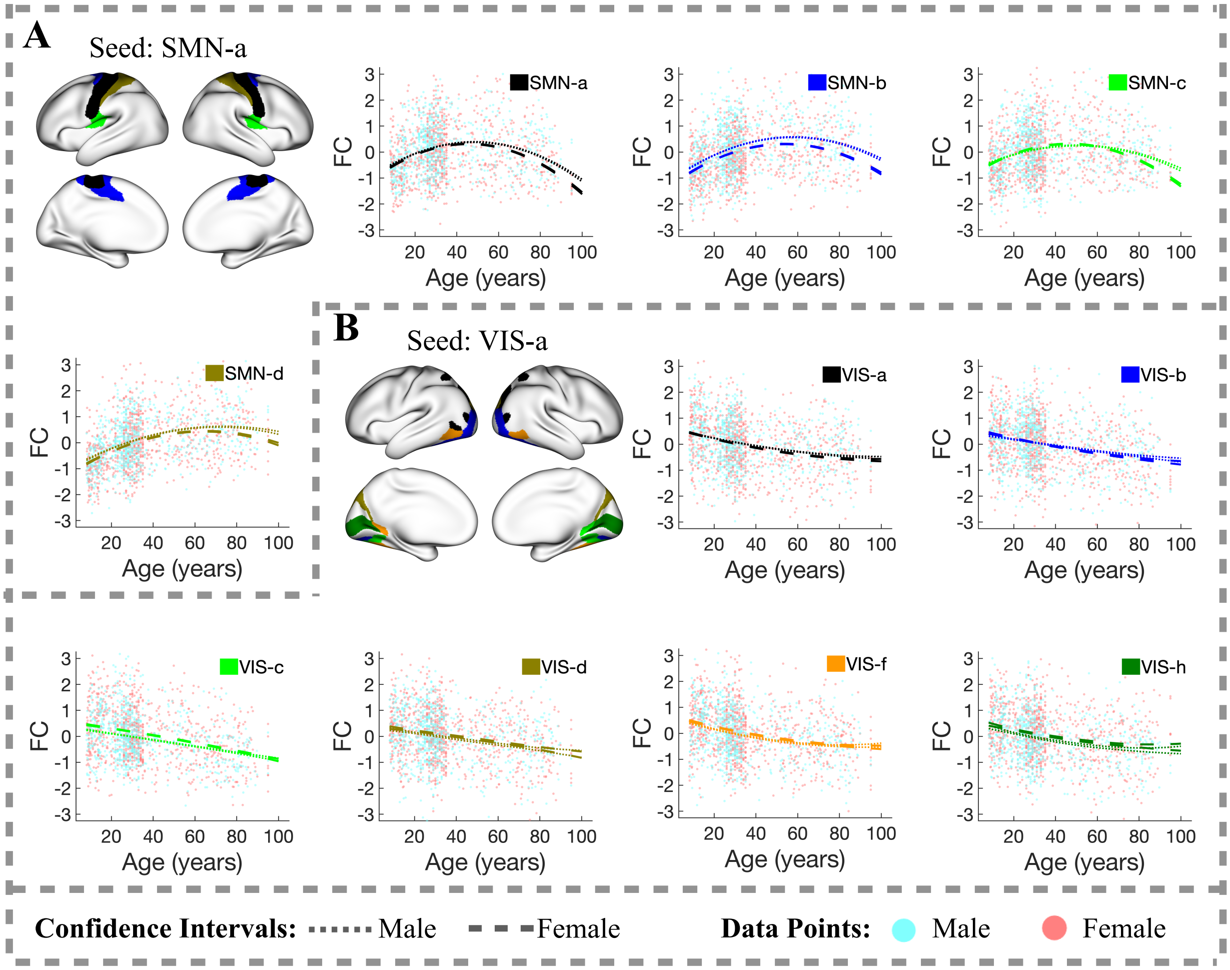

Figure S26: Within-Network FC trajectories of the SMN-a and VIS-a with R-squared values exceeding 5%. In each brain plot, one region cluster is designated as the seed cluster, plotted in black. The trajectory of the FC between the seed cluster and another cluster from the same functional network, say cluster B, is plotted in the same color assigned to cluster B. Light cyan and light coral dots in the FC plots represent FC values between a region in the seed cluster and another region in cluster B for individual male and female subjects, respectively. Dotted lines represent 95% confidence intervals for population-mean FC trajectories of males, whereas dashed lines represent those of females.

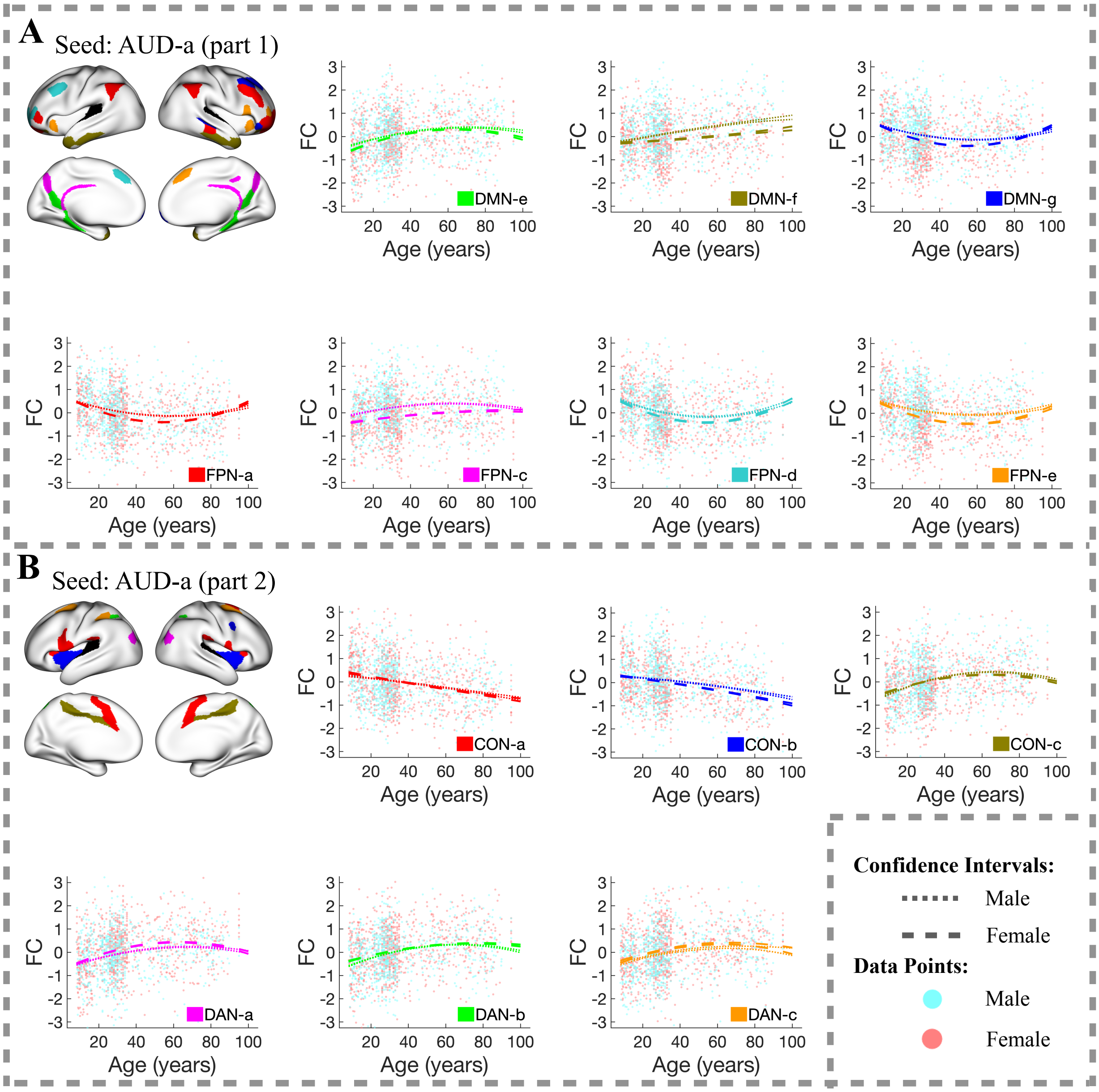

Figure S27: Between-network FC trajectories of the AUD-a with R-squared values exceeding 5%. In each brain plot, the AUD-a is designated as the seed cluster, plotted in black. The FC trajectory between the seed cluster and another cluster from a different functional network, say cluster B, is plotted in the same color assigned to cluster B. Light cyan and light coral dots in the FC plots represent FC values between a region in the AUD-a and another region in cluster B for individual male and female subjects, respectively. Dotted lines represent 95% confidence intervals for population-mean FC trajectories of males, whereas dashed lines represent those of females.

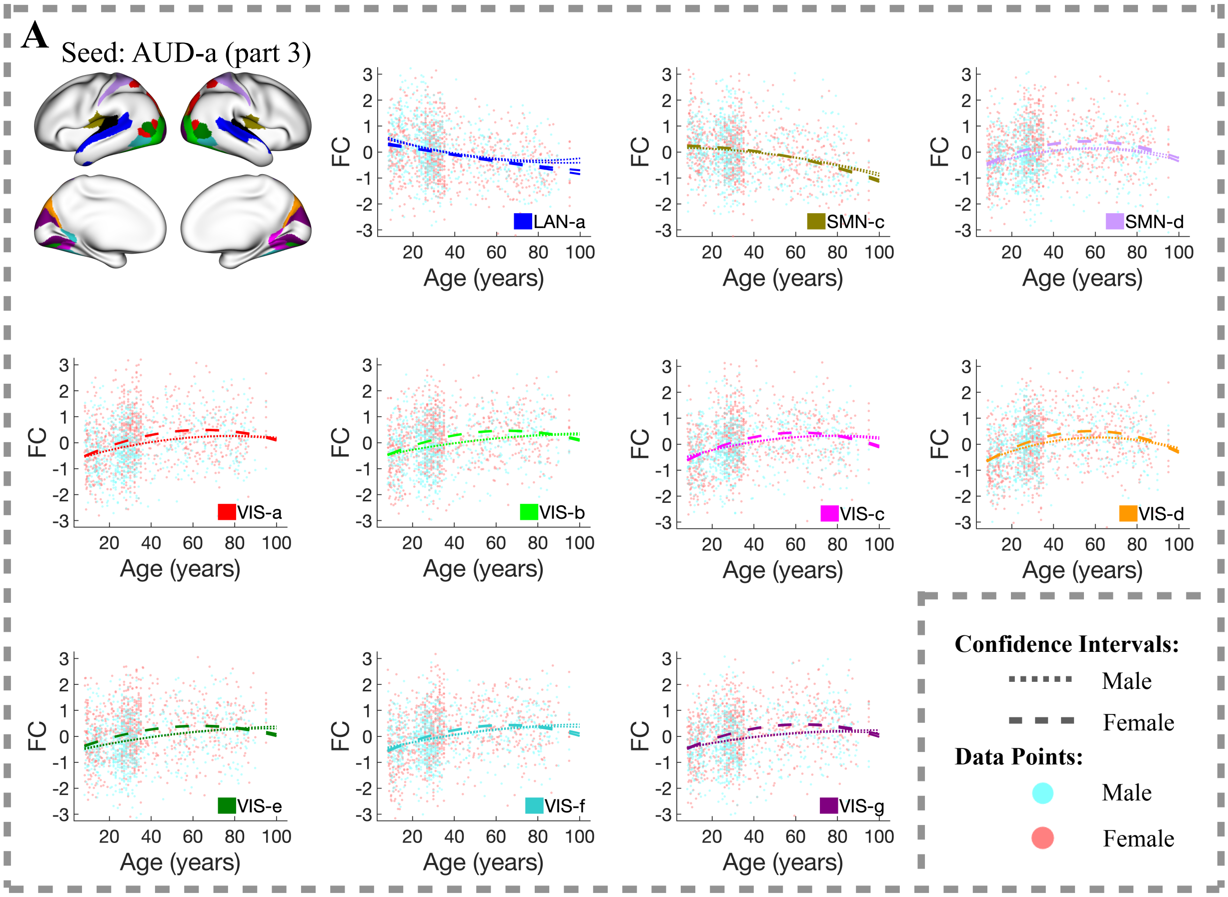

Figure S28: Between-network FC trajectories of the AUD-a with R-squared values exceeding 5%. In each brain plot, the AUD-a is designated as the seed cluster, plotted in black. The FC trajectory between the seed cluster and another cluster from a different functional network, say cluster B, is plotted in the same color assigned to cluster B. Light cyan and light coral dots in the FC plots represent FC values between a region in the AUD-a and another region in cluster B for individual male and female subjects, respectively. Dotted lines represent 95% confidence intervals for population-mean FC trajectories of males, whereas dashed lines represent those of females.

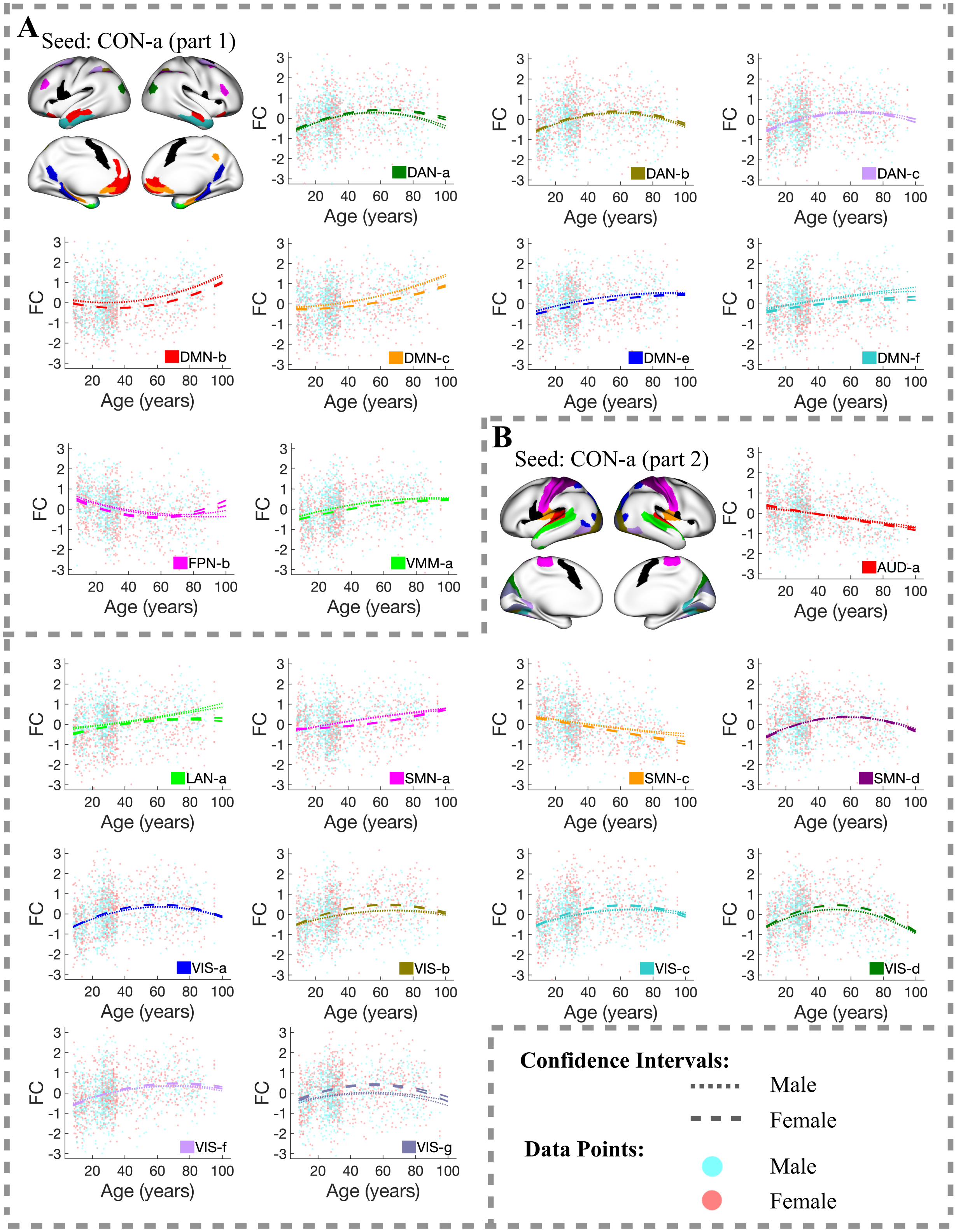

Figure S29: Between-network FC trajectories of the CON-a with R-squared values exceeding 5%. In each brain plot, the CON-a is designated as the seed cluster, plotted in black. The FC trajectory between the seed cluster and another cluster from a different functional network, say cluster B, is plotted in the same color assigned to cluster B. Light cyan and light coral dots in the FC plots represent FC values between a region in the CON-a and another region in cluster B for individual male and female subjects, respectively. Dotted lines represent 95% confidence intervals for population-mean FC trajectories of males, whereas dashed lines represent those of females.

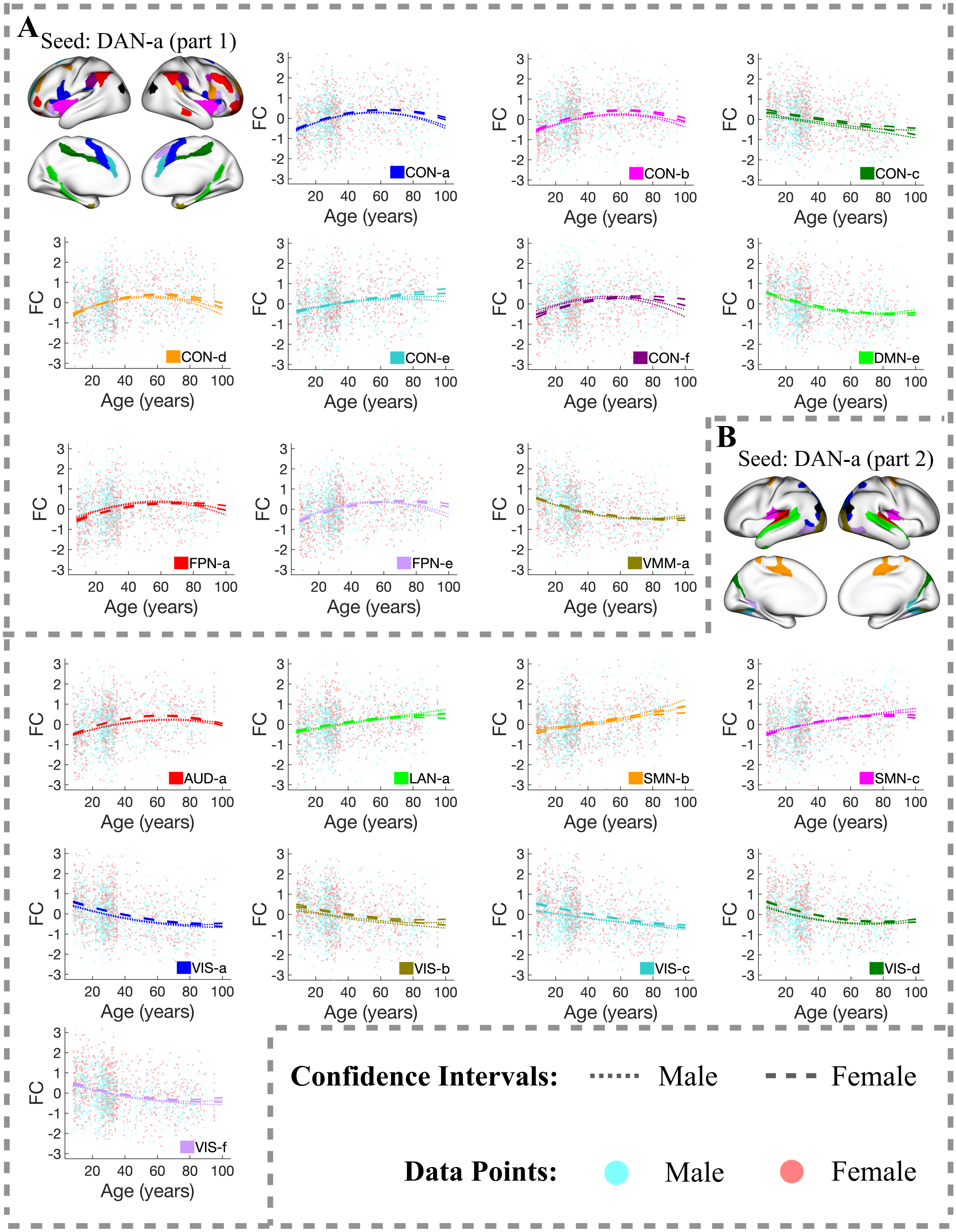

Figure S30: Between-network FC trajectories of the DAN-a with R-squared values exceeding 5%. In each brain plot, the DAN-a is designated as the seed cluster, plotted in black. The FC trajectory between the seed cluster and another cluster from a different functional network, say cluster B, is plotted in the same color assigned to cluster B. Light cyan and light coral dots in the FC plots represent FC values between a region in the DAN-a and another region in cluster B for individual male and female subjects, respectively. Dotted lines represent 95% confidence intervals for population-mean FC trajectories of males, whereas dashed lines represent those of females.

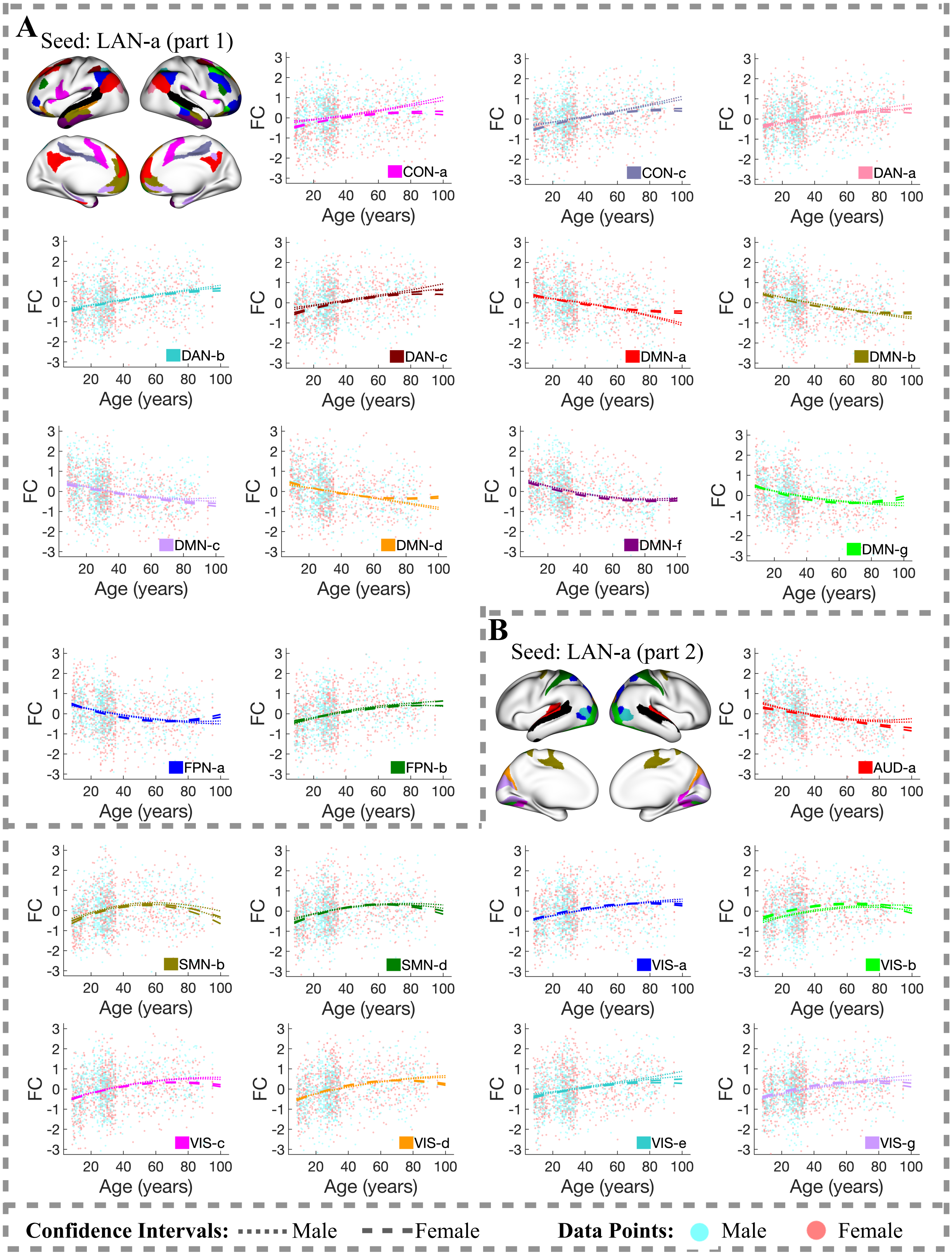

Figure S31: Between-network FC trajectories of the LAN-a with R-squared values exceeding 5%. In each brain plot, the LAN-a is designated as the seed cluster, plotted in black. The FC trajectory between the seed cluster and another cluster from a different functional network, say cluster B, is plotted in the same color assigned to cluster B. Light cyan and light coral dots in the FC plots represent FC values between a region in the LAN-a and another region in cluster B for individual male and female subjects, respectively. Dotted lines represent 95% confidence intervals for population-mean FC trajectories of males, whereas dashed lines represent those of females.

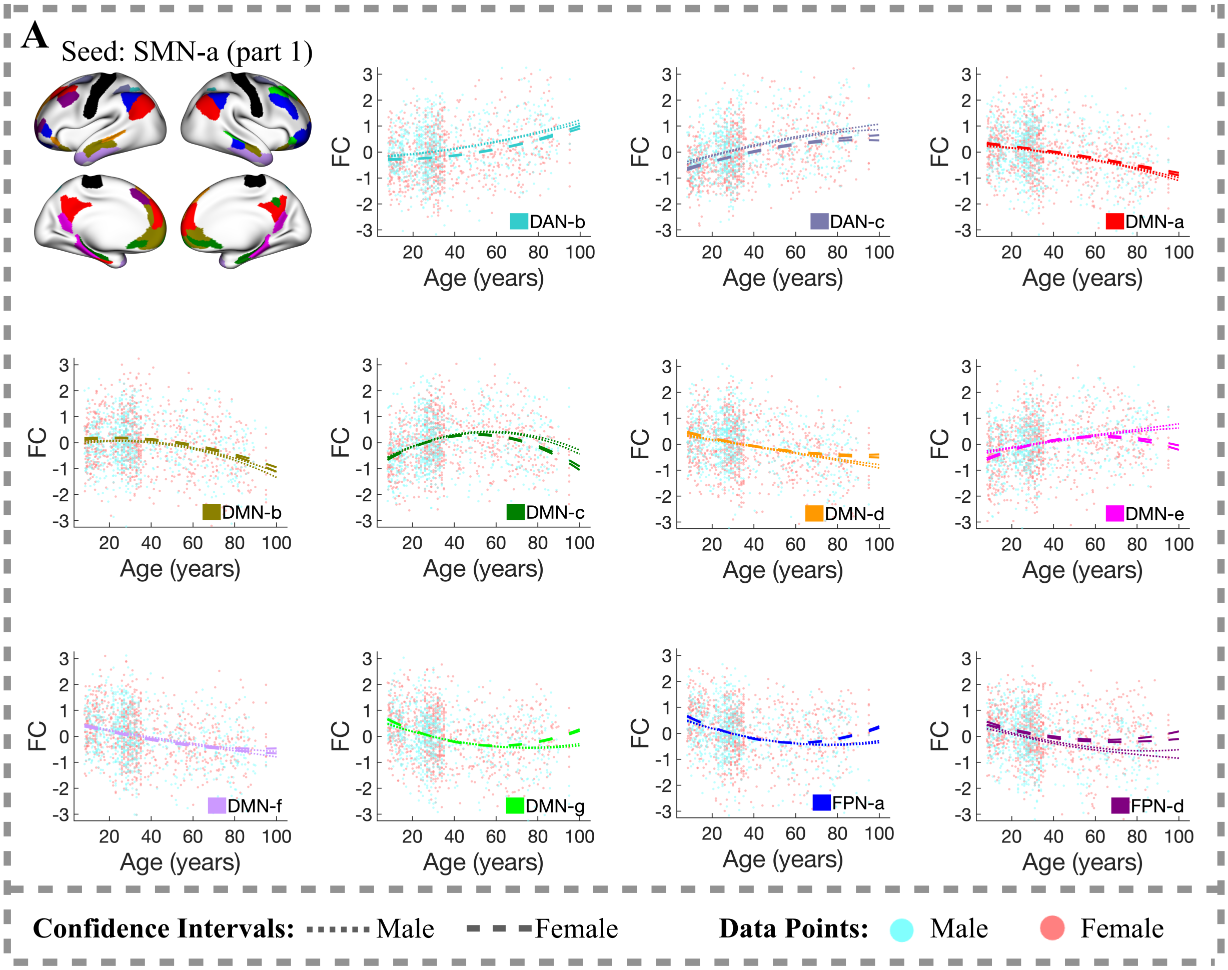

Figure S32: Between-network FC trajectories of the SMN-a with R-squared values exceeding 5%. In each brain plot, the SMN-a is designated as the seed cluster, plotted in black. The FC trajectory between the seed cluster and another cluster from a different functional network, say cluster B, is plotted in the same color assigned to cluster B. Light cyan and light coral dots in the FC plots represent FC values between a region in the SMN-a and another region in cluster B for individual male and female subjects, respectively. Dotted lines represent 95% confidence intervals for population-mean FC trajectories of males, whereas dashed lines represent those of females.

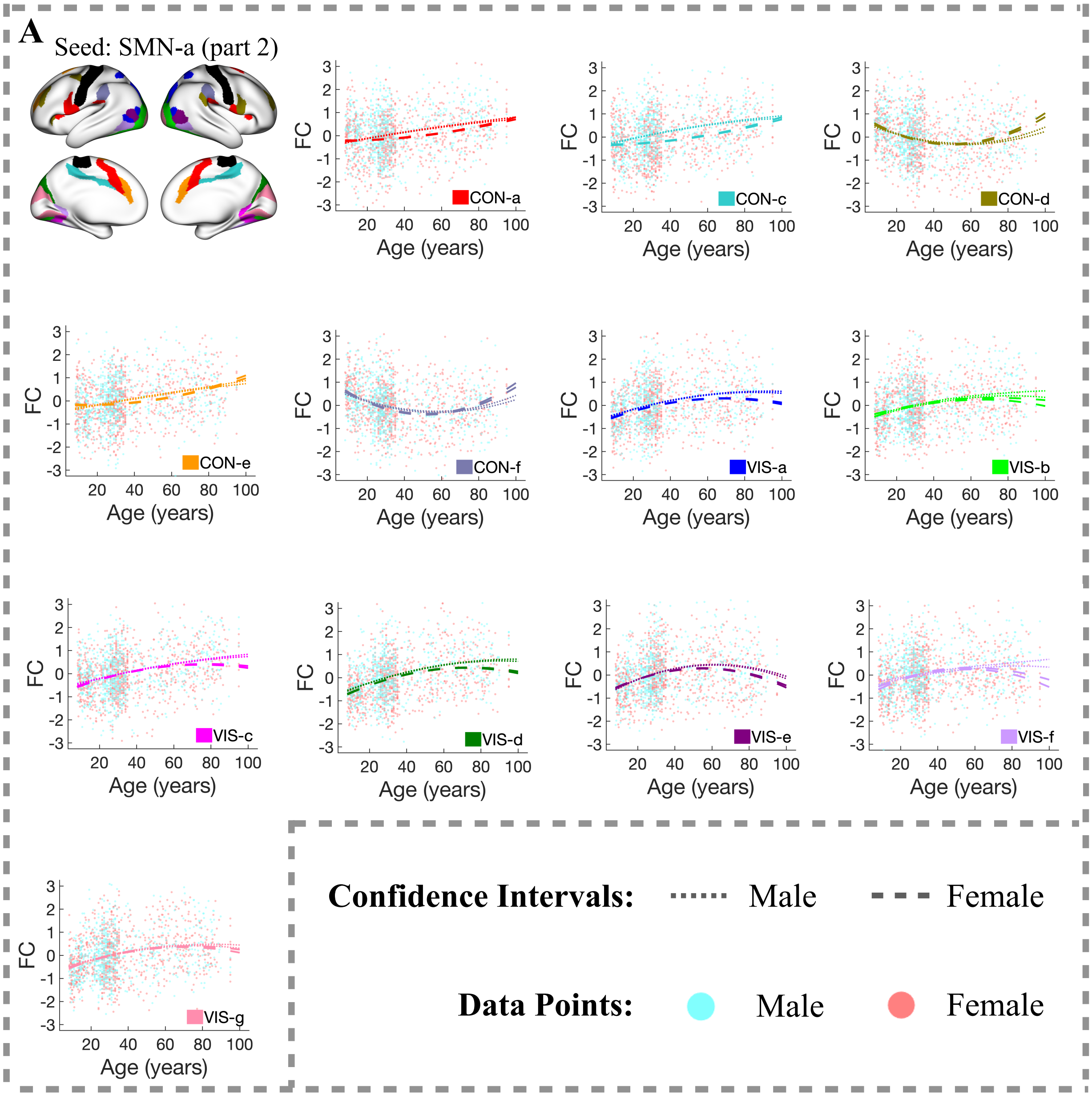

Figure S33: Between-network FC trajectories of the SMN-a with R-squared values exceeding 5%. In each brain plot, the SMN-a is designated as the seed cluster, plotted in black. The FC trajectory between the seed cluster and another cluster from a different functional network, say cluster B, is plotted in the same color assigned to cluster B. Light cyan and light coral dots in the FC plots represent FC values between a region in the SMN-a and another region in cluster B for individual male and female subjects, respectively. Dotted lines represent 95% confidence intervals for population-mean FC trajectories of males, whereas dashed lines represent those of females.

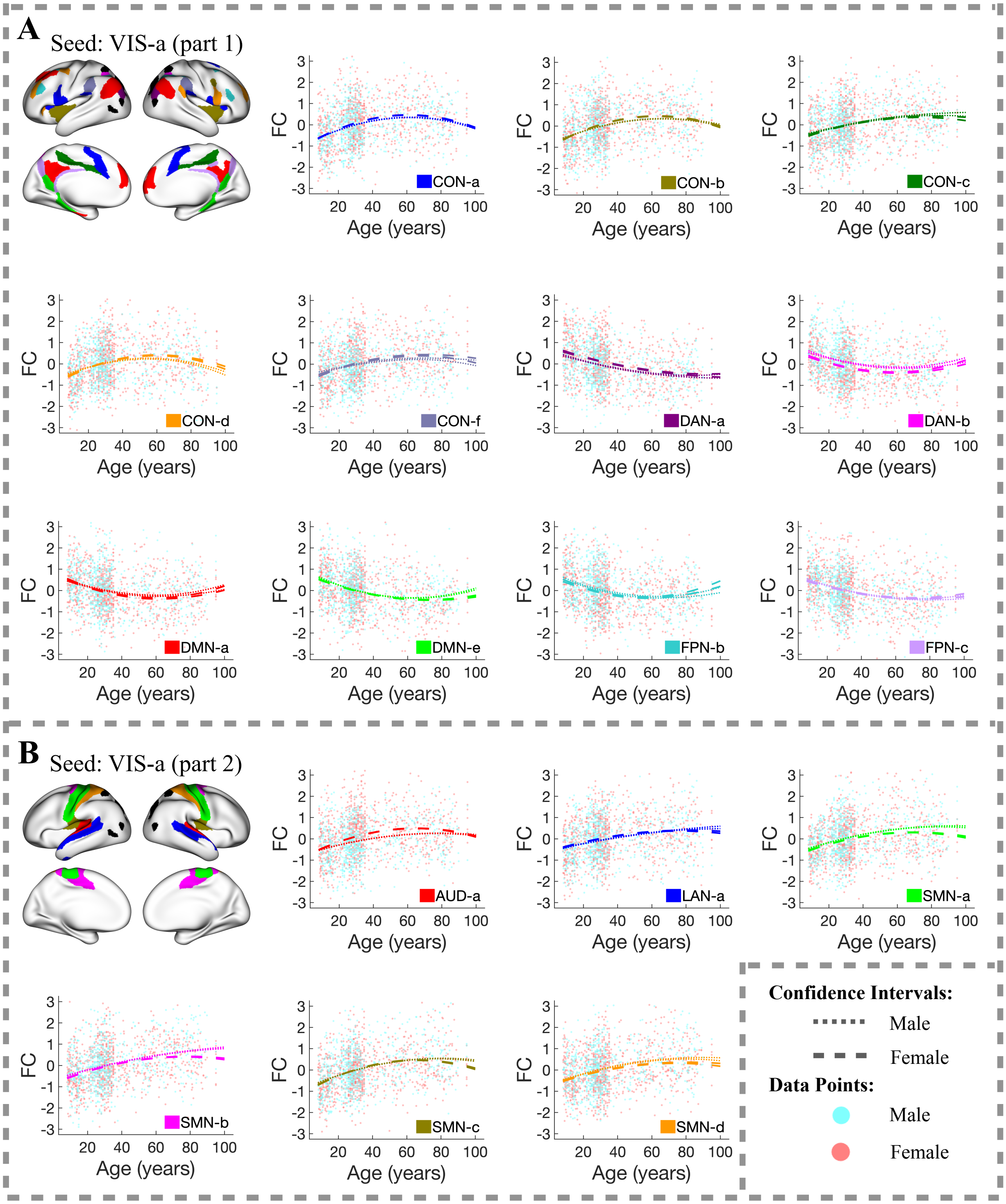

Figure S34: Between-network FC trajectories of the VIS-a with R-squared values exceeding 5%. In each brain plot, the VIS-a is designated as the seed cluster, plotted in black. The FC trajectory between the seed cluster and another cluster from a different functional network, say cluster B, is plotted in the same color assigned to cluster B. Light cyan and light coral dots in the FC plots represent FC values between a region in the VIS-a and another region in cluster B for individual male and female subjects, respectively. Dotted lines represent 95% confidence intervals for population-mean FC trajectories of males, whereas dashed lines represent those of females.

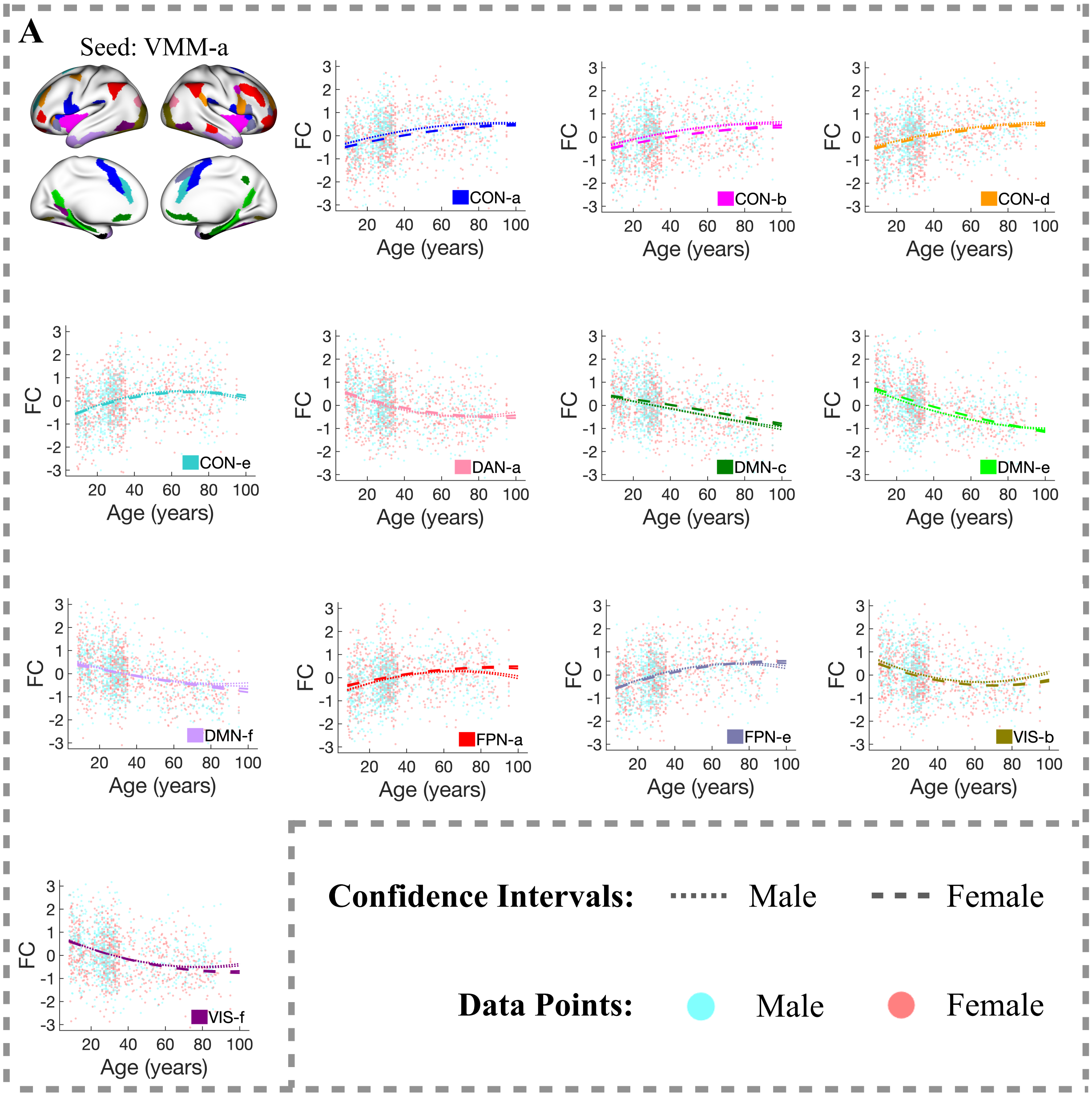

Figure S35: Between-network FC trajectories of the VMM-a with R-squared values exceeding 5%. In each brain plot, the VMM-a is designated as the seed cluster, plotted in black. The FC trajectory between the seed cluster and another cluster from a different functional network, say cluster B, is plotted in the same color assigned to cluster B. Light cyan and light coral dots in the FC plots represent FC values between a region in the VMM-a and another region in cluster B for individual male and female subjects, respectively. Dotted lines represent 95% confidence intervals for population-mean FC trajectories of males, whereas dashed lines represent those of females.

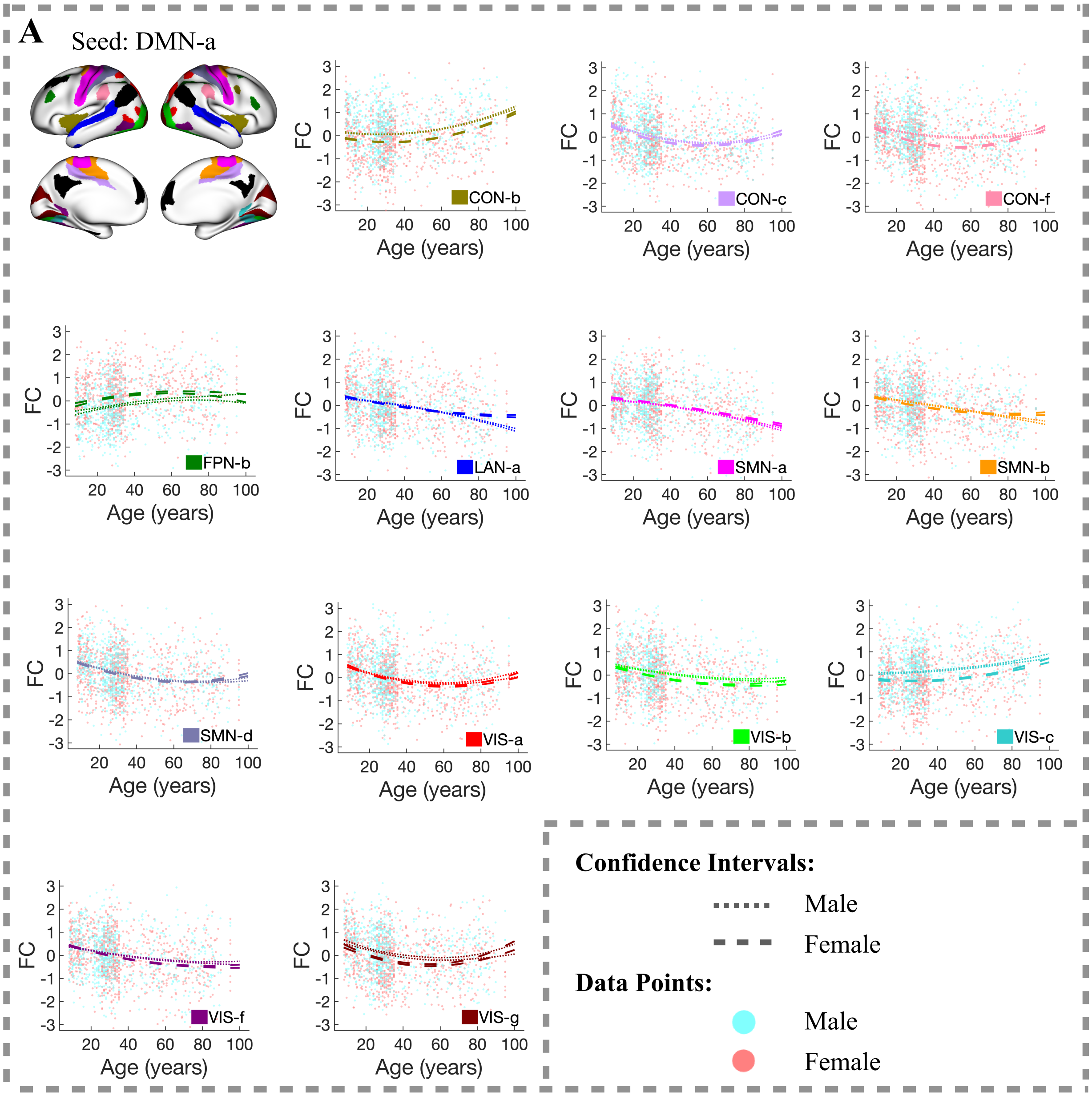

Figure S36: Between-network FC trajectories of the DMN-a with R-squared values exceeding 5%. In each brain plot, the DMN-a is designated as the seed cluster, plotted in black. The FC trajectory between the seed cluster and another cluster from a different functional network, say cluster B, is plotted in the same color assigned to cluster B. Light cyan and light coral dots in the FC plots represent FC values between a region in the seed cluster and another region in cluster B for individual male and female subjects, respectively. Dotted lines represent 95% confidence intervals for population-mean FC trajectories of males, whereas dashed lines represent those of females.

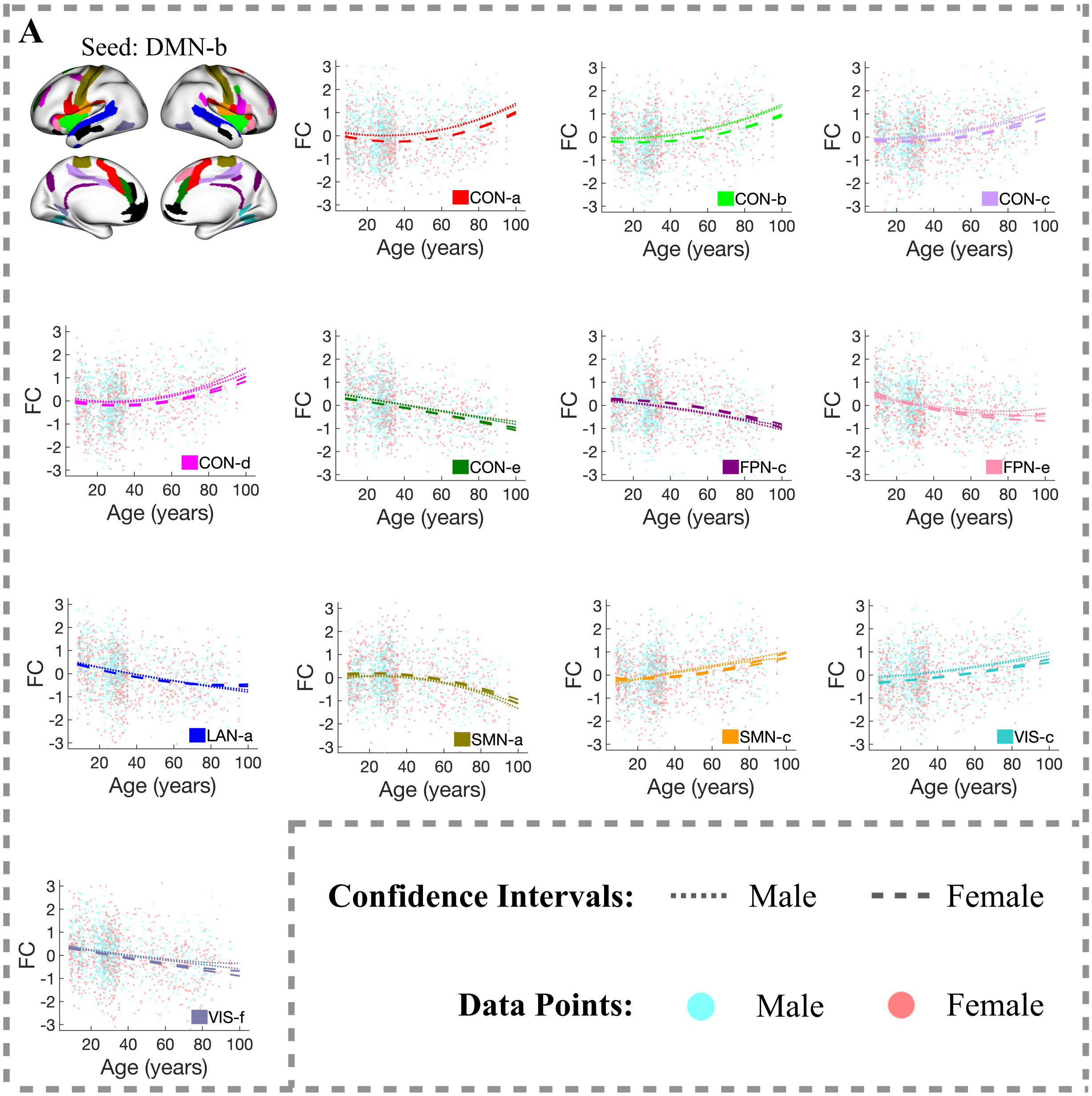

Figure S37: Between-network FC trajectories of the DMN-b with R-squared values exceeding 5%. In each brain plot, the DMN-b is designated as the seed cluster, plotted in black. The FC trajectory between the seed cluster and another cluster from a different functional network, say cluster B, is plotted in the same color assigned to cluster B. Light cyan and light coral dots in the FC plots represent FC values between a region in the seed cluster and another region in cluster B for individual male and female subjects, respectively. Dotted lines represent 95% confidence intervals for population-mean FC trajectories of males, whereas dashed lines represent those of females.

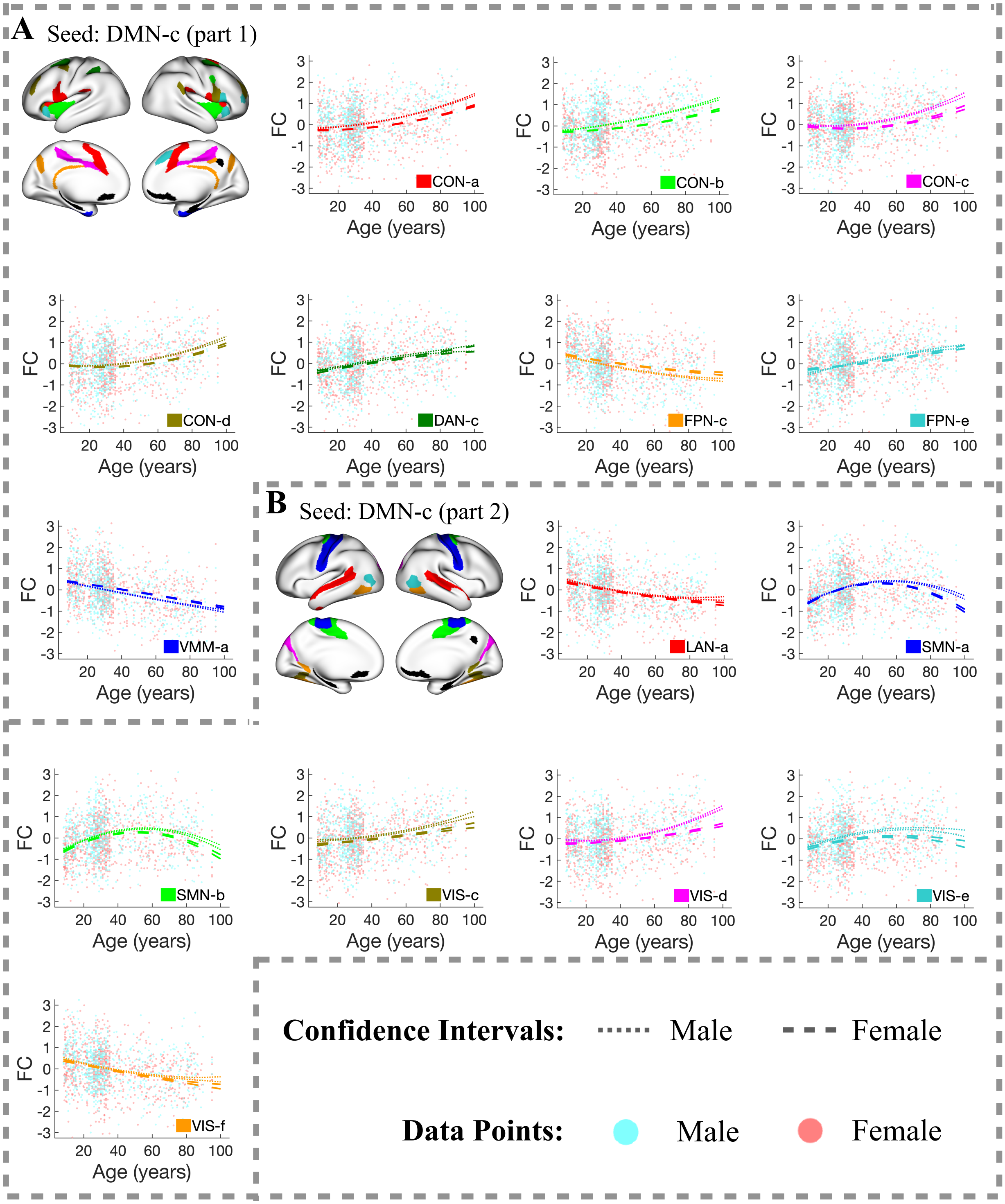

Figure S38: Between-network FC trajectories of the DMN-c with R-squared values exceeding 5%. In each brain plot, the DMN-c is designated as the seed cluster, plotted in black. The FC trajectory between the seed cluster and another cluster from a different functional network, say cluster B, is plotted in the same color assigned to cluster B. Light cyan and light coral dots in the FC plots represent FC values between a region in the seed cluster and another region in cluster B for individual male and female subjects, respectively. Dotted lines represent 95% confidence intervals for population-mean FC trajectories of males, whereas dashed lines represent those of females.

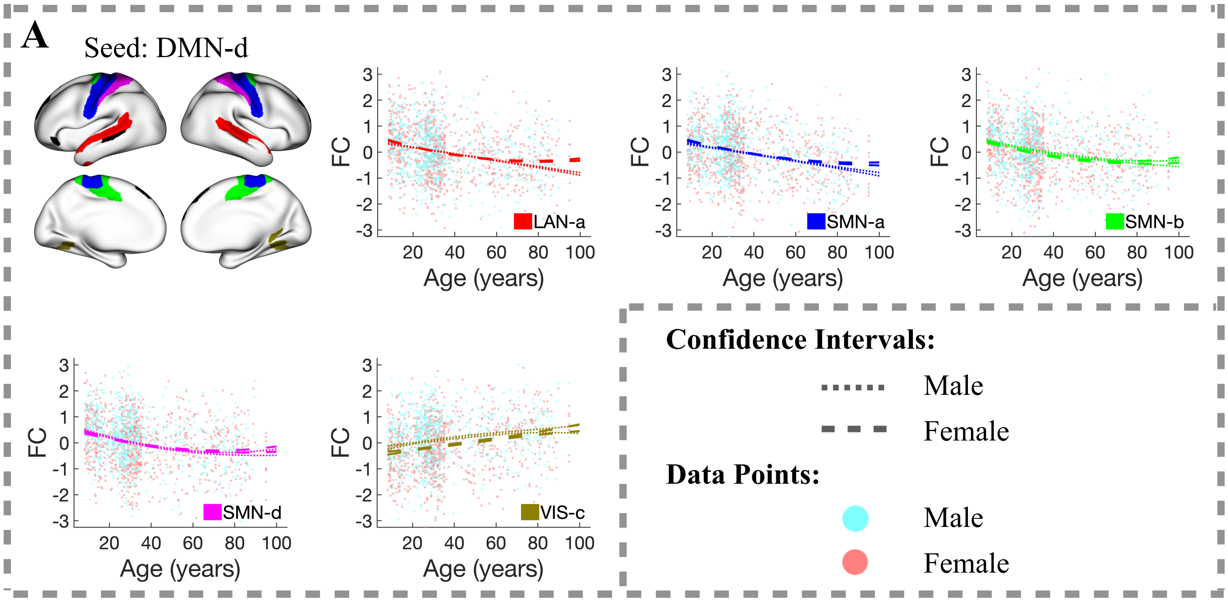

Figure S39: Between-network FC trajectories of the DMN-d with R-squared values exceeding 5%. In each brain plot, the DMN-d is designated as the seed cluster, plotted in black. The FC trajectory between the seed cluster and another cluster from a different functional network, say cluster B, is plotted in the same color assigned to cluster B. Light cyan and light coral dots in the FC plots represent FC values between a region in the seed cluster and another region in cluster B for individual male and female subjects, respectively. Dotted lines represent 95% confidence intervals for population-mean FC trajectories of males, whereas dashed lines represent those of females.

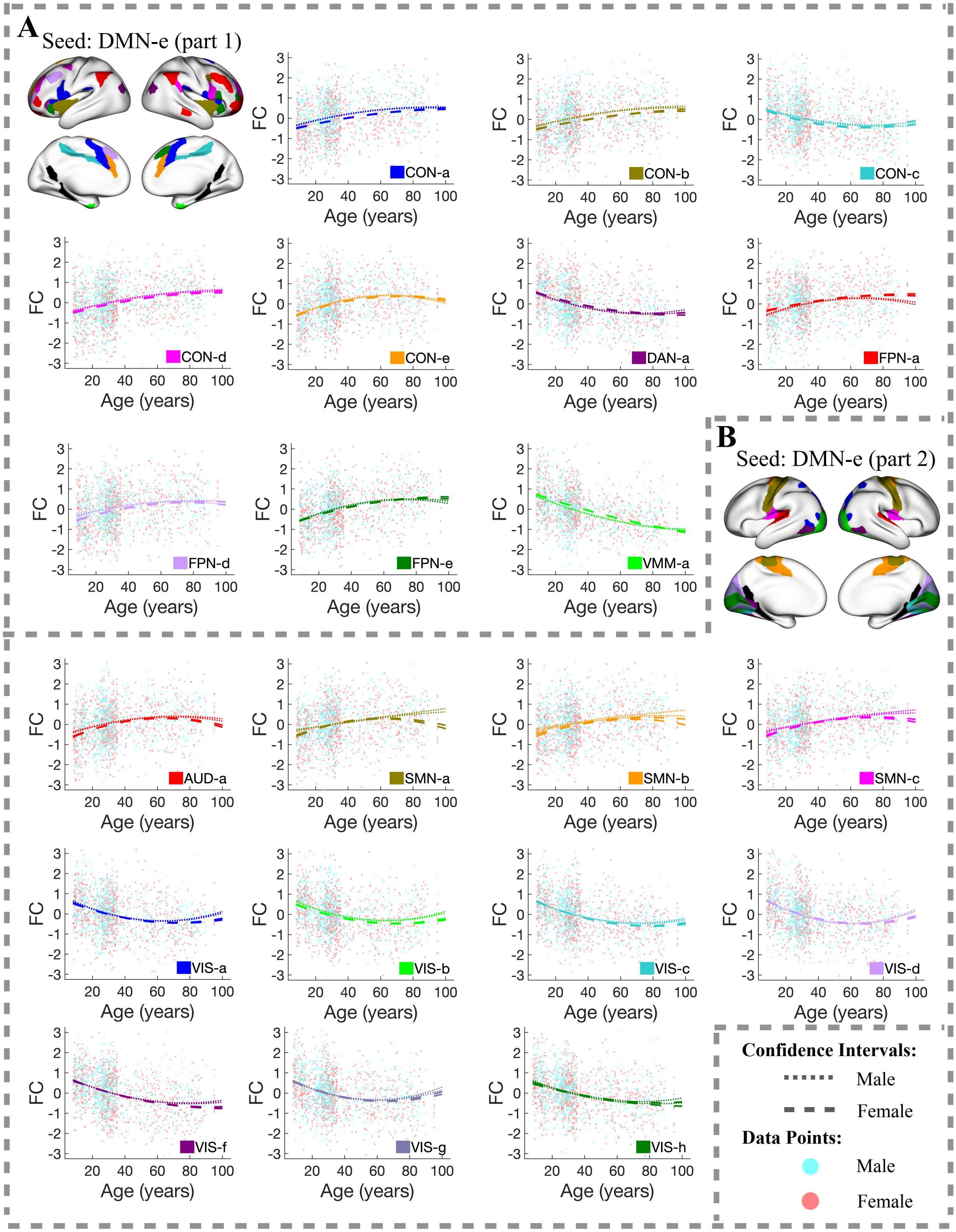

Figure S40: Between-network FC trajectories of the DMN-e with R-squared values exceeding 5%. In each brain plot, the DMN-e is designated as the seed cluster, plotted in black. The FC trajectory between the seed cluster and another cluster from a different functional network, say cluster B, is plotted in the same color assigned to cluster B. Light cyan and light coral dots in the FC plots represent FC values between a region in the seed cluster and another region in cluster B for individual male and female subjects, respectively. Dotted lines represent 95% confidence intervals for population-mean FC trajectories of males, whereas dashed lines represent those of females.

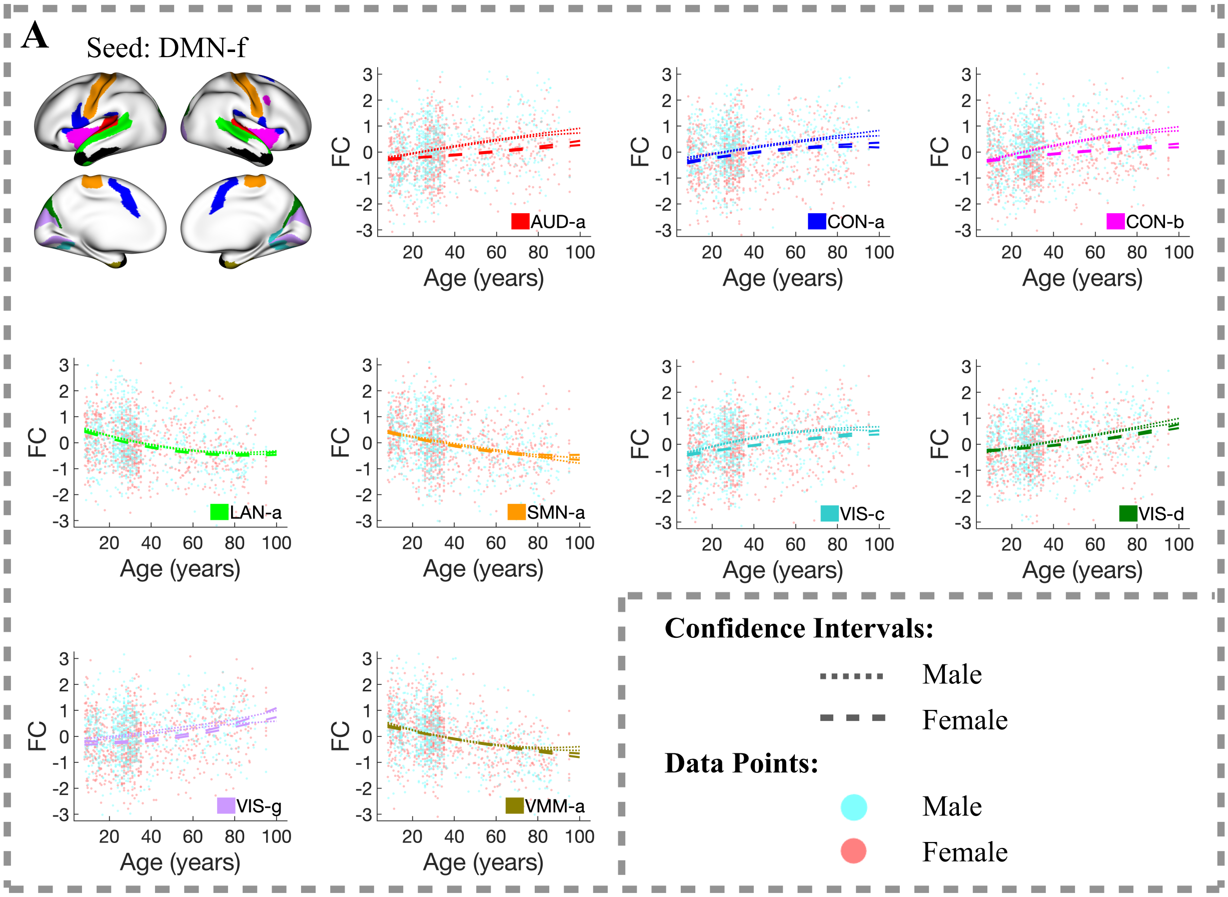

Figure S41: Between-network FC trajectories of the DMN-f with R-squared values exceeding 5%. In each brain plot, the DMN-f is designated as the seed cluster, plotted in black. The FC trajectory between the seed cluster and another cluster from a different functional network, say cluster B, is plotted in the same color assigned to cluster B. Light cyan and light coral dots in the FC plots represent FC values between a region in the seed cluster and another region in cluster B for individual male and female subjects, respectively. Dotted lines represent 95% confidence intervals for population-mean FC trajectories of males, whereas dashed lines represent those of females.

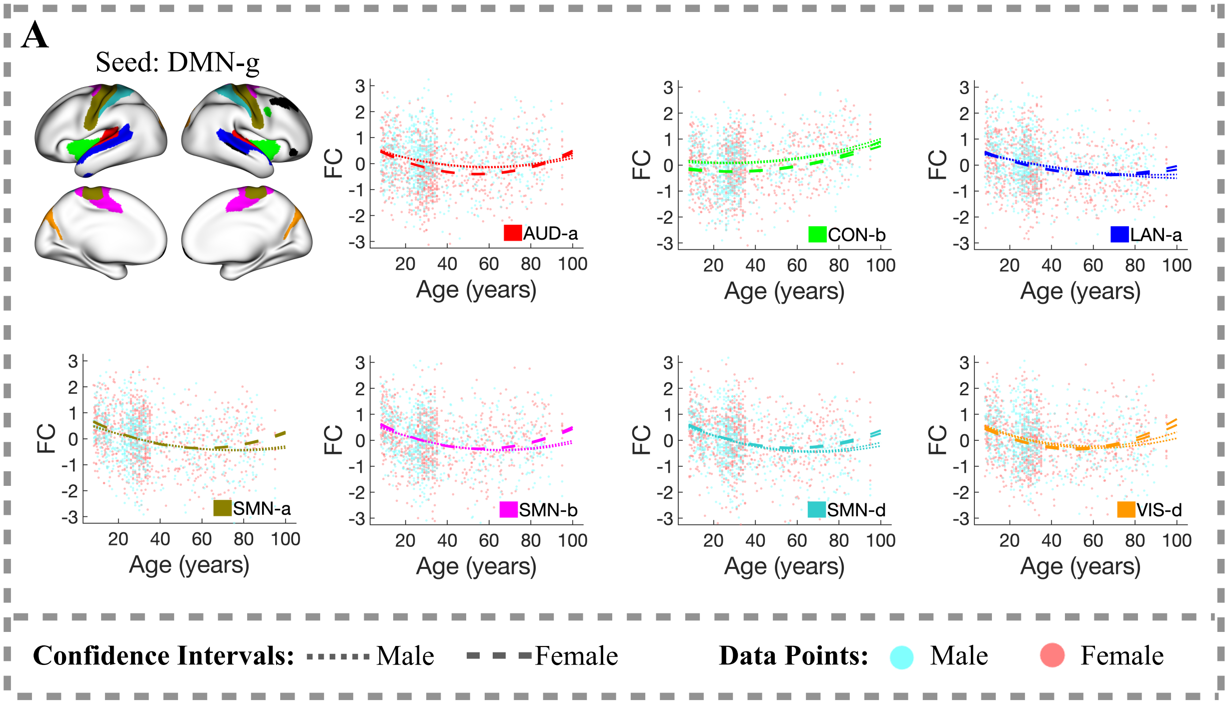

Figure S42: Between-network FC trajectories of the DMN-g with R-squared values exceeding 5%. In each brain plot, the DMN-g is designated as the seed cluster, plotted in black. The FC trajectory between the seed cluster and another cluster from a different functional network, say cluster B, is plotted in the same color assigned to cluster B. Light cyan and light coral dots in the FC plots represent FC values between a region in the seed cluster and another region in cluster B for individual male and female subjects, respectively. Dotted lines represent 95% confidence intervals for population-mean FC trajectories of males, whereas dashed lines represent those of females.

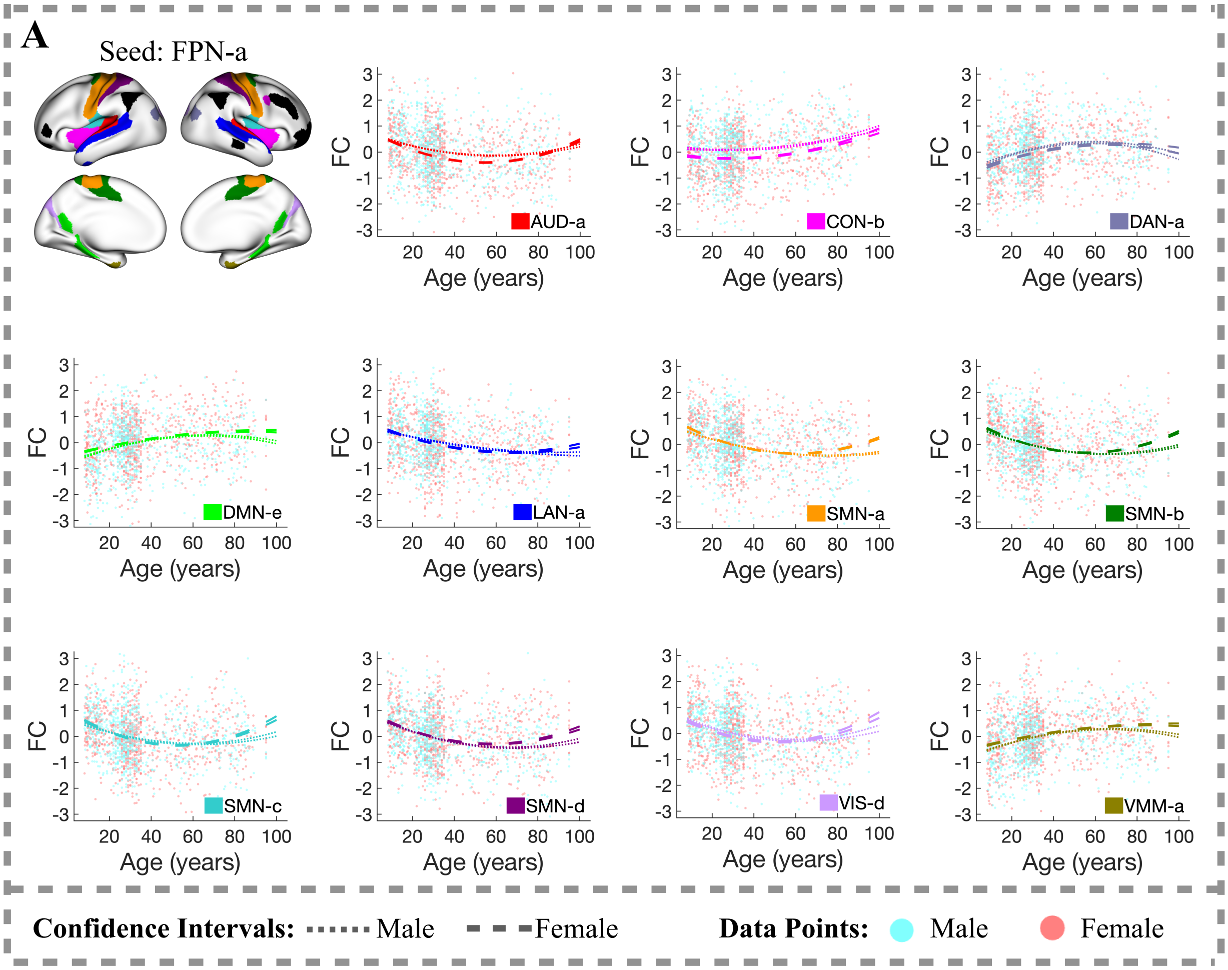

Figure S43: Between-network FC trajectories of the FPN-a with R-squared values exceeding 5%. In each brain plot, the FPN-a is designated as the seed cluster, plotted in black. The FC trajectory between the seed cluster and another cluster from a different functional network, say cluster B, is plotted in the same color assigned to cluster B. Light cyan and light coral dots in the FC plots represent FC values between a region in the seed cluster and another region in cluster B for individual male and female subjects, respectively. Dotted lines represent 95% confidence intervals for population-mean FC trajectories of males, whereas dashed lines represent those of females.

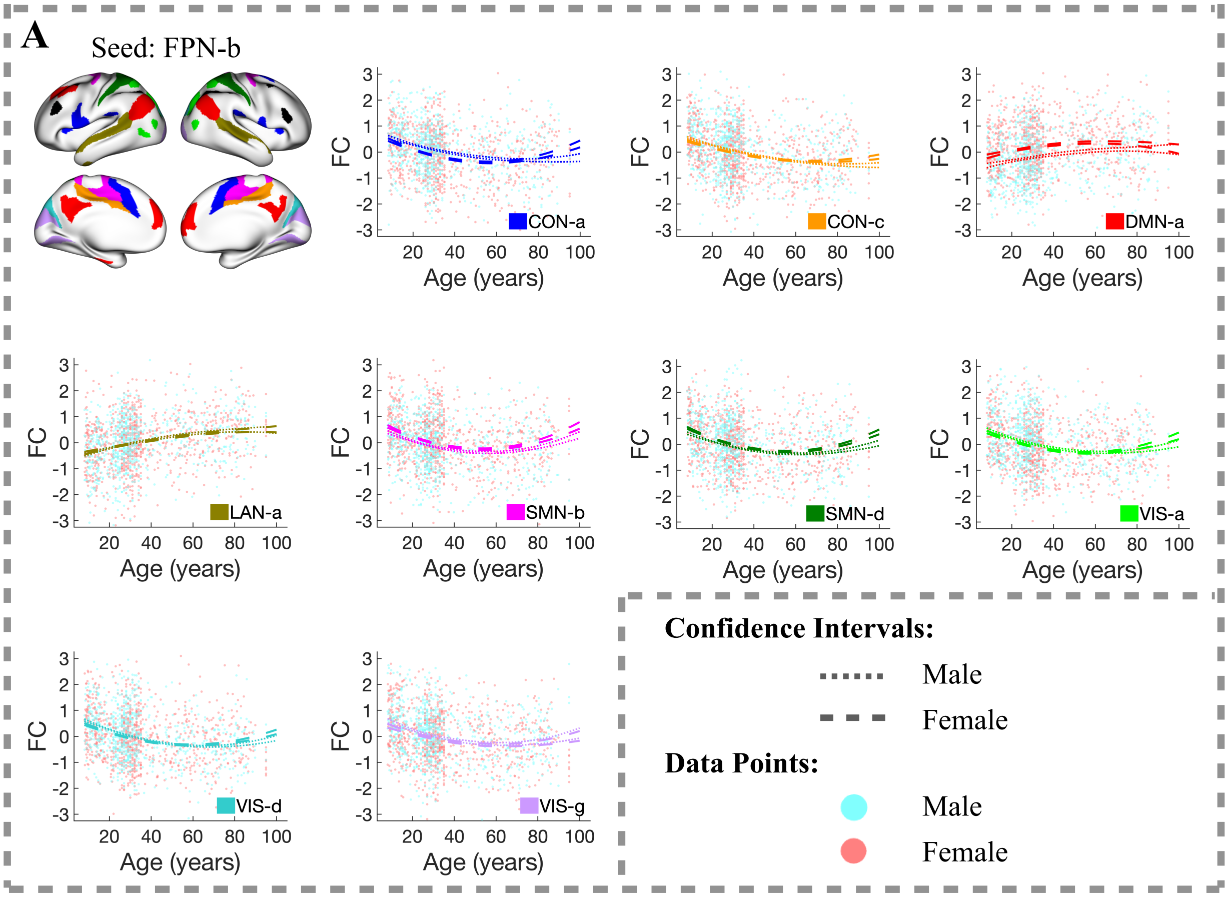

Figure S44: Between-network FC trajectories of the FPN-b with R-squared values exceeding 5%. In each brain plot, the FPN-b is designated as the seed cluster, plotted in black. The FC trajectory between the seed cluster and another cluster from a different functional network, say cluster B, is plotted in the same color assigned to cluster B. Light cyan and light coral dots in the FC plots represent FC values between a region in the seed cluster and another region in cluster B for individual male and female subjects, respectively. Dotted lines represent 95% confidence intervals for population-mean FC trajectories of males, whereas dashed lines represent those of females.

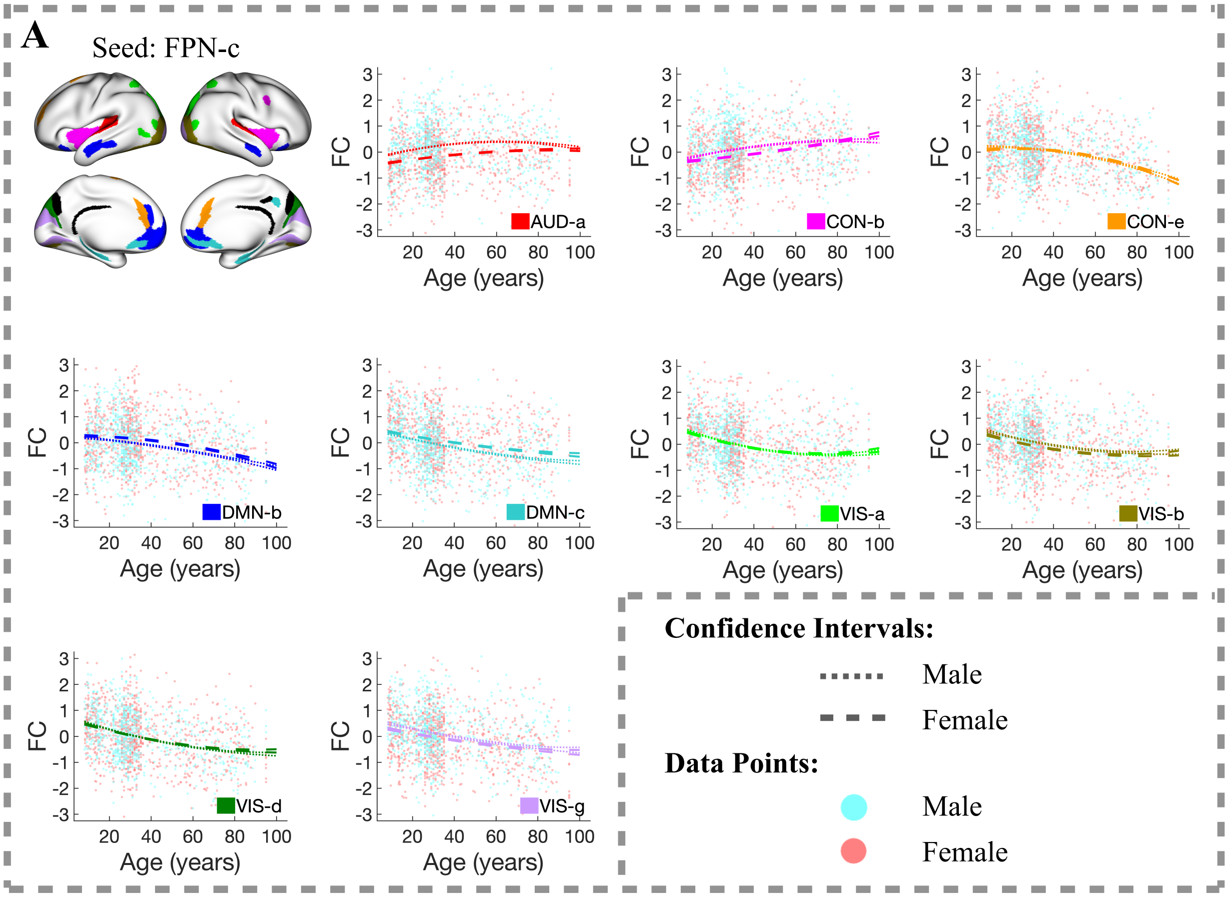

Figure S45: Between-network FC trajectories of the FPN-c with R-squared values exceeding 5%. In each brain plot, the FPN-c is designated as the seed cluster, plotted in black. The FC trajectory between the seed cluster and another cluster from a different functional network, say cluster B, is plotted in the same color assigned to cluster B. Light cyan and light coral dots in the FC plots represent FC values between a region in the seed cluster and another region in cluster B for individual male and female subjects, respectively. Dotted lines represent 95% confidence intervals for population-mean FC trajectories of males, whereas dashed lines represent those of females.

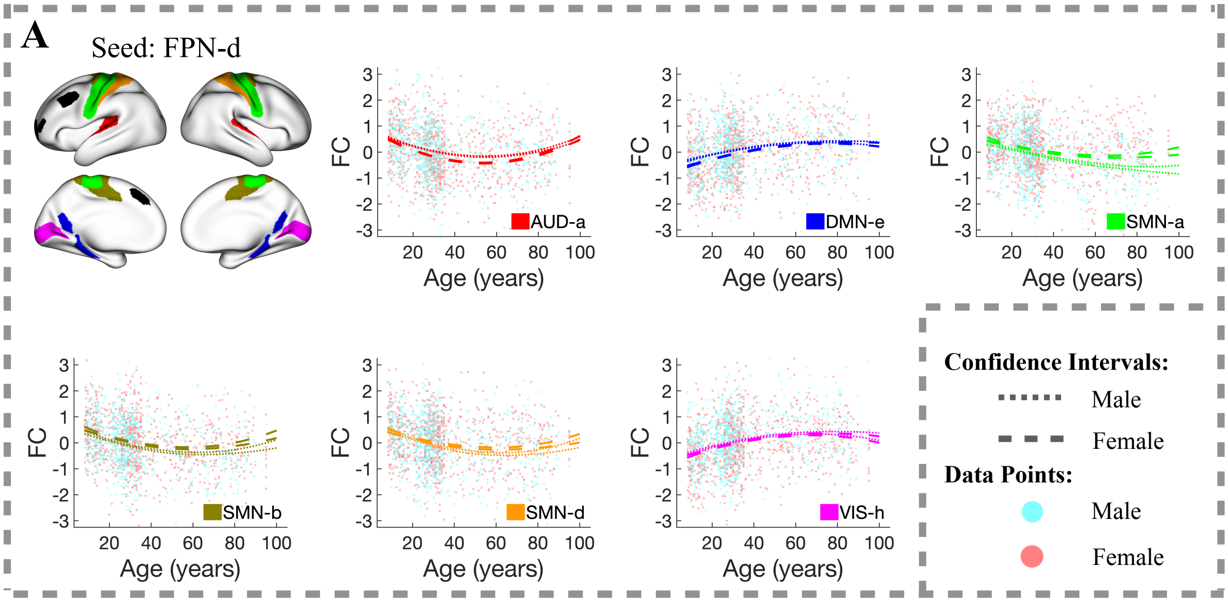

Figure S46: Between-network FC trajectories of the FPN-d with R-squared values exceeding 5%. In each brain plot, the FPN-d is designated as the seed cluster, plotted in black. The FC trajectory between the seed cluster and another cluster from a different functional network, say cluster B, is plotted in the same color assigned to cluster B. Light cyan and light coral dots in the FC plots represent FC values between a region in the seed cluster and another region in cluster B for individual male and female subjects, respectively. Dotted lines represent 95% confidence intervals for population-mean FC trajectories of males, whereas dashed lines represent those of females.

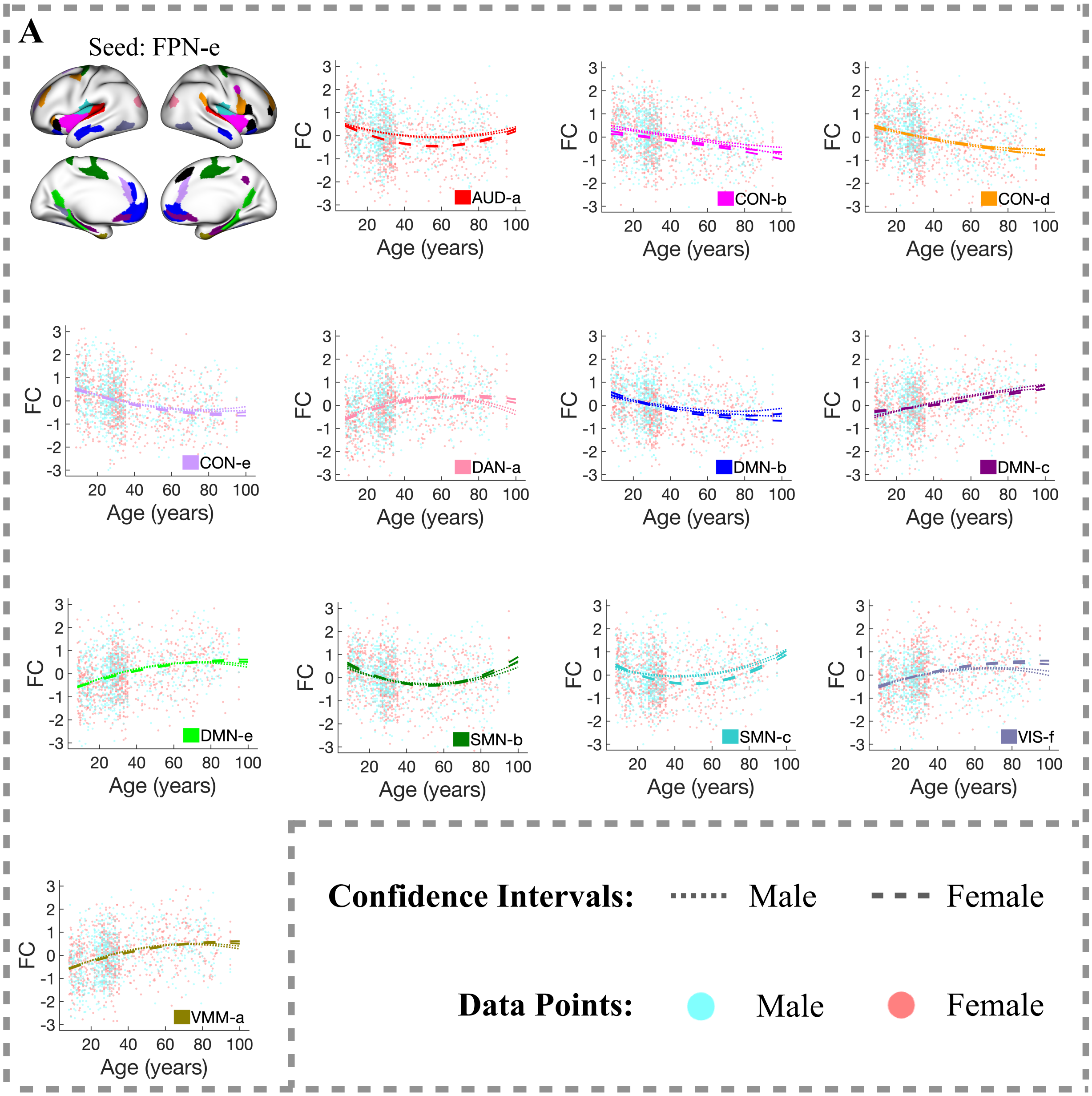

Figure S47: Between-network FC trajectories of the FPN-e with R-squared values exceeding 5%. In each brain plot, the FPN-e is designated as the seed cluster, plotted in black. The FC trajectory between the seed cluster and another cluster from a different functional network, say cluster B, is plotted in the same color assigned to cluster B. Light cyan and light coral dots in the FC plots represent FC values between a region in the seed cluster and another region in cluster B for individual male and female subjects, respectively. Dotted lines represent 95% confidence intervals for population-mean FC trajectories of males, whereas dashed lines represent those of females.

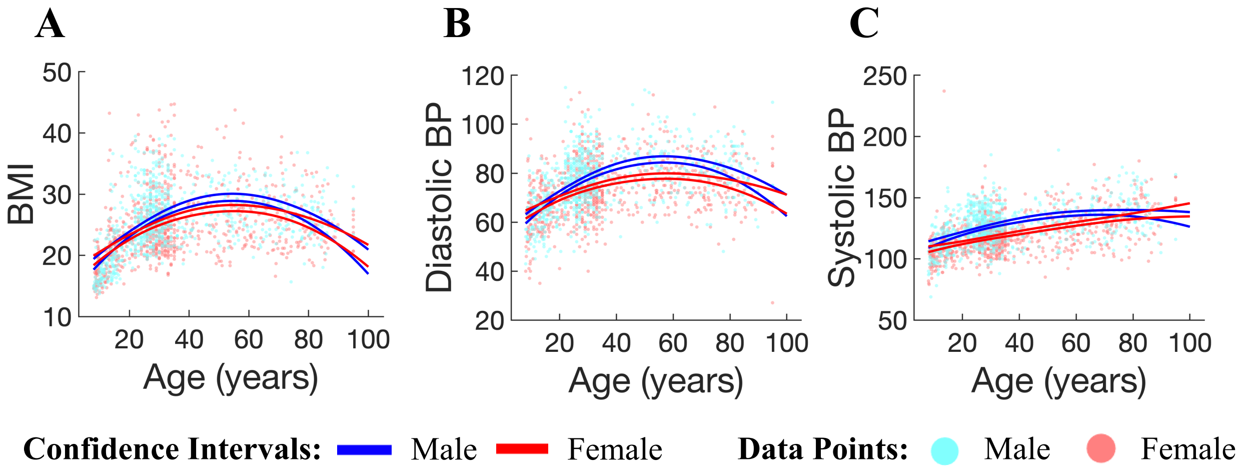

Figure S48: Plots of BMI, diastolic BP, and systolic BP versus age and sex. Light cyan and light coral dots represent the three measurements of male and female subjects, respectively. Blue and red lines represent 95% confidence intervals for the population-mean trajectories of BMI, diastolic BP, and systolic BP for males and females, respectively.

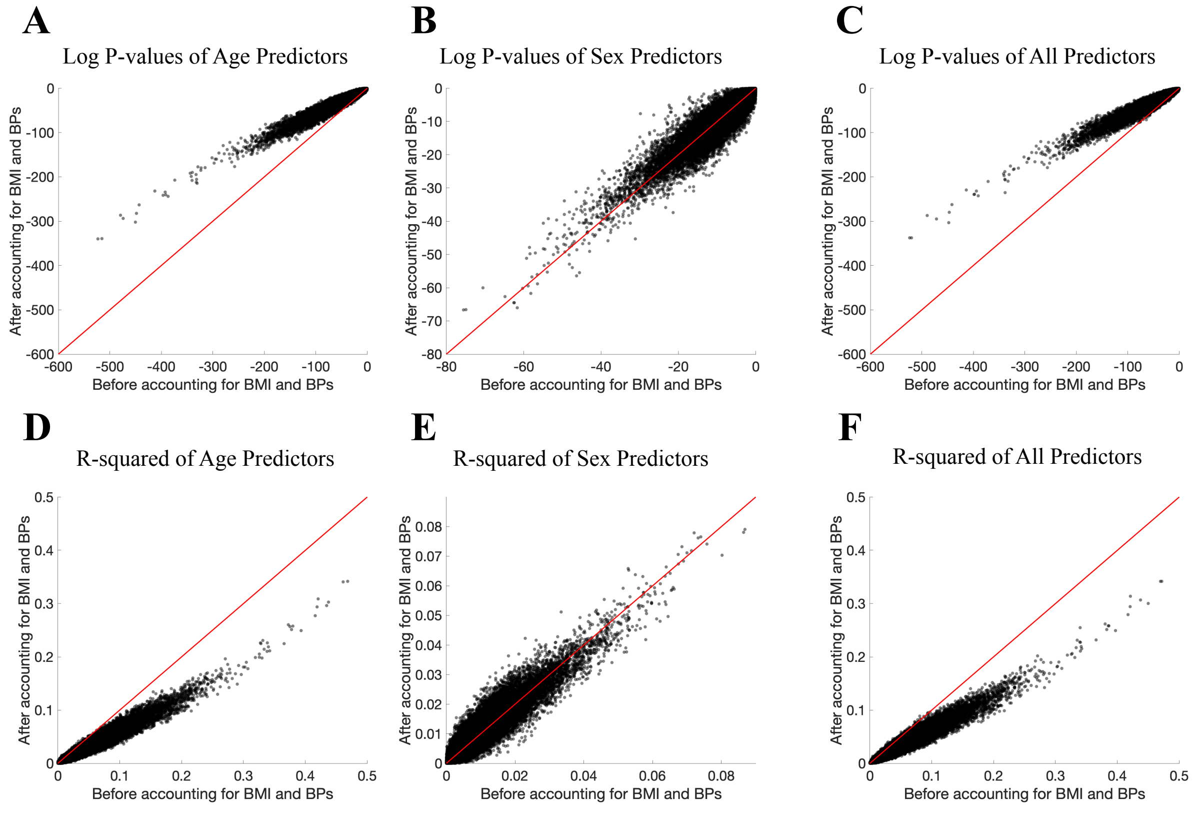

Figure S49: Comparison of the significance of age-related, sex-related, and all predictors before and after adjusting for BMI, diastolic BP, and systolic BP. A-C: Scatter plots comparing log p-values of age-related predictors, sex-related predictors, and all predictors from independent regression analyses of FC without and with the inclusion of the three cardiac health factors across all connections. D-F: Scatter plots comparing R-squared values of age-related predictors, sex-related predictors, and all predictors from independent regression analyses of FC without and with the inclusion of the three cardiac health factors across all connections.

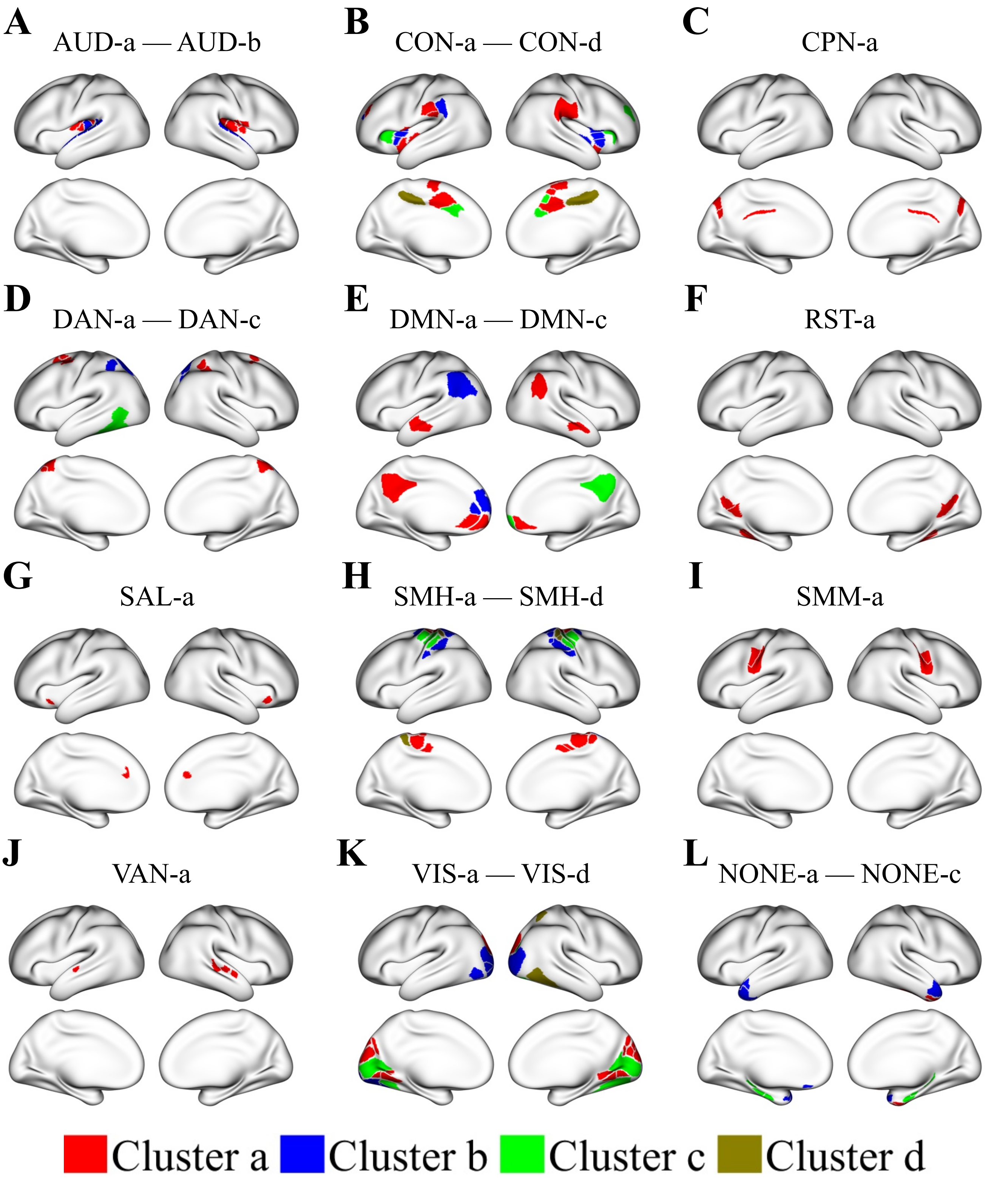

Figure S50: Region clusters identified using the Gordon parcellation. Each region cluster consists of at least four regions or 500 grayordinates from the same functional network. Within a single functional network, region clusters are plotted in different colors and labeled (e.g., a, b, etc.) in a descending order based on their region counts, from the largest to the smallest. The functional networks include the auditory (AUD), cingulo-opercular (CON), cingulo-parietal (CPN), dorsal attention (DAN), default mode (DMN), retrosplenial/temporal (RST), salience (SAL), somatomotor hand (SMH), somatomotor mouth (SMM), ventral attention (VAN) and visual (VIS) networks. Regions without a clear functional identity are labeled as NONE.

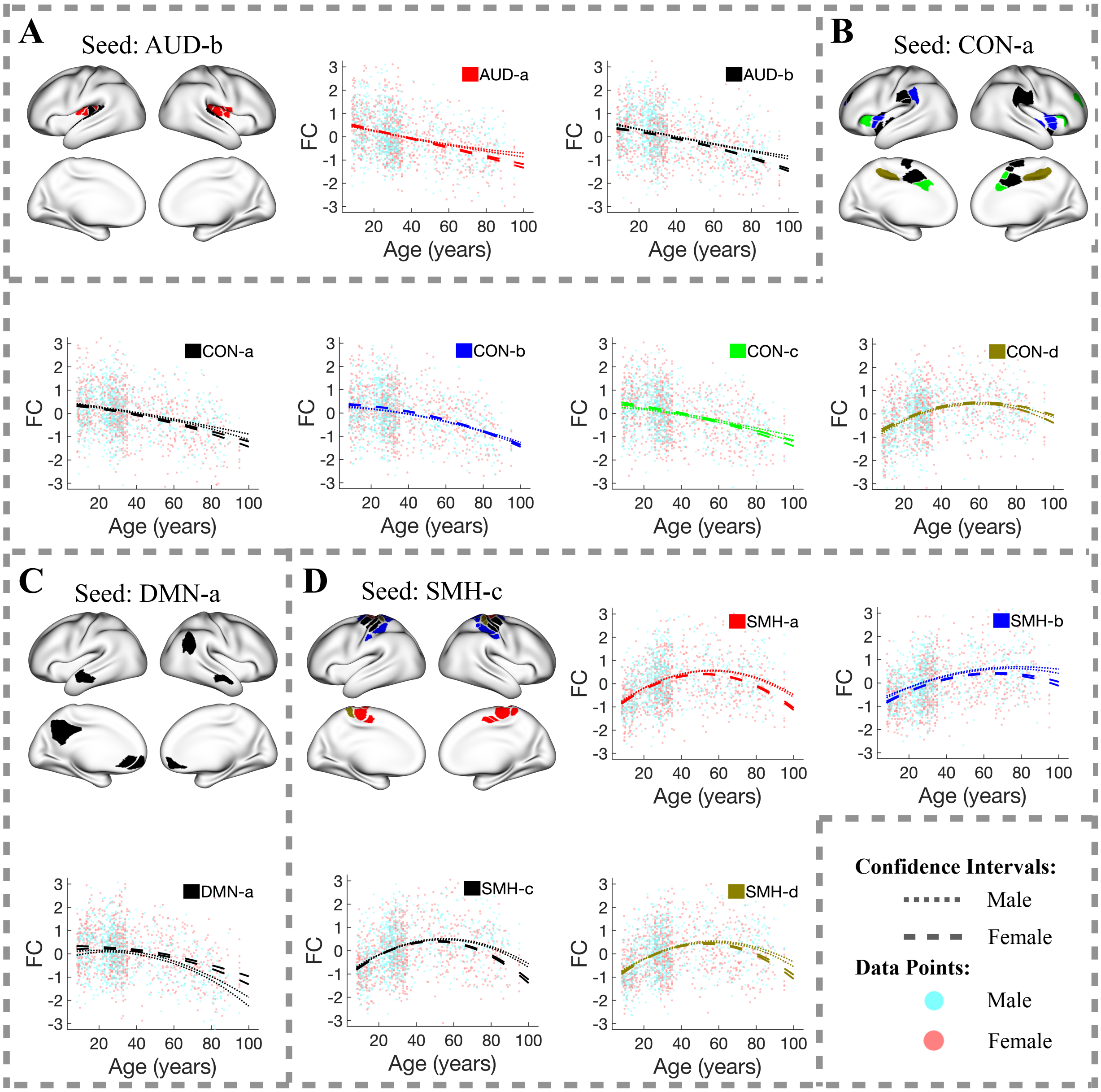

Figure S51: Within-network FC trajectories with R-squared values exceeding 10% using the Gordon parcellation. In each brain plot, one region cluster is designated as the seed cluster, plotted in black. The FC trajectory between the seed cluster and a cluster from the same functional network, referred to as cluster B, is displayed in the color assigned to cluster B. Light cyan and light coral dots in the FC plots represent FC values between a region in the seed cluster and another region in cluster B for individual male and female subjects, respectively. Dotted lines represent 95% confidence intervals for population-mean FC trajectories of males, whereas dashed lines represent those of females.

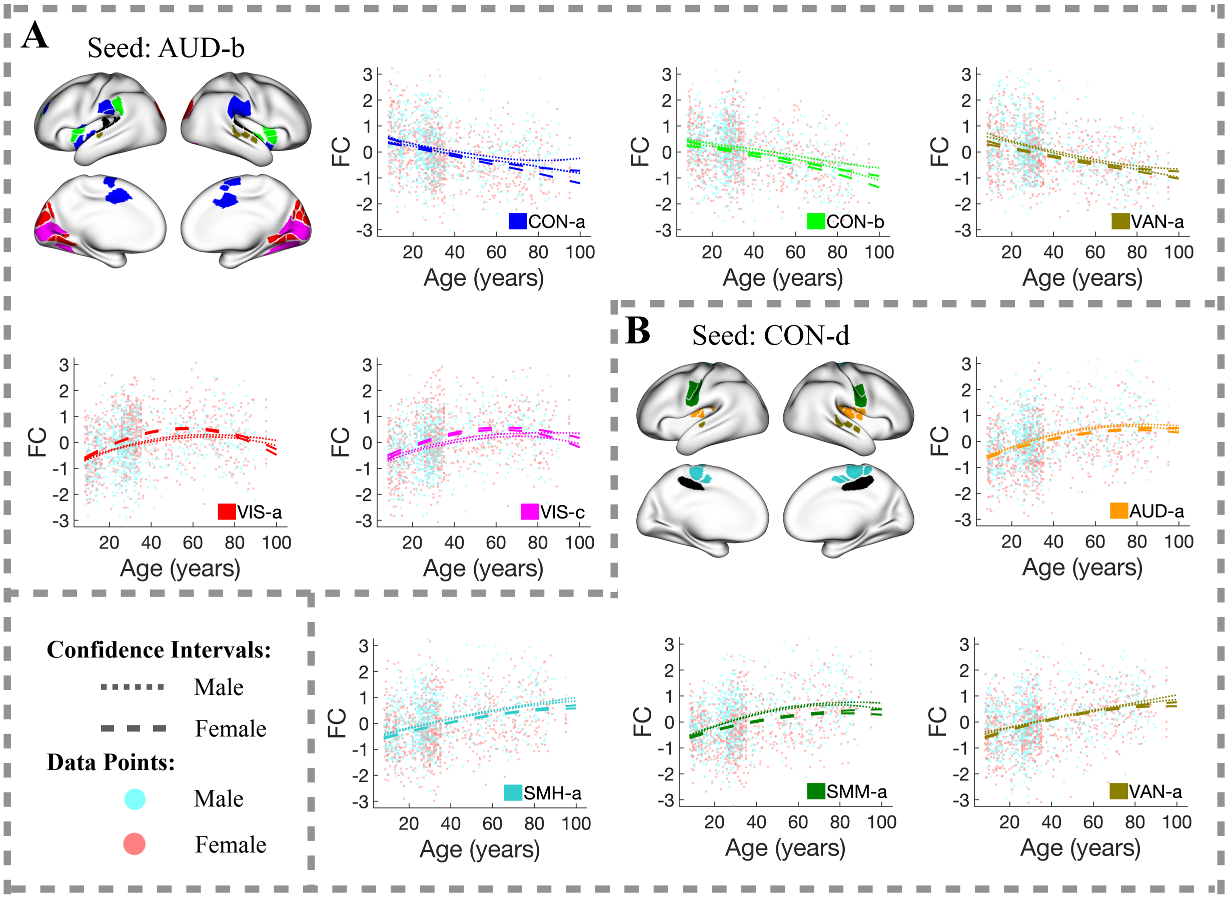

Figure S52: Between-network FC trajectories with R-squared values exceeding 10% using the Gordon parcellation. In each brain plot, one region cluster is designated as the seed cluster, plotted in black. The FC trajectory between the seed cluster and a cluster from the same functional network, referred to as cluster B, is displayed in the color assigned to cluster B. Light cyan and light coral dots in the FC plots represent FC values between a region in the seed cluster and another region in cluster B for individual male and female subjects, respectively. Dotted lines represent 95% confidence intervals for population-mean FC trajectories of males, whereas dashed lines represent those of females.

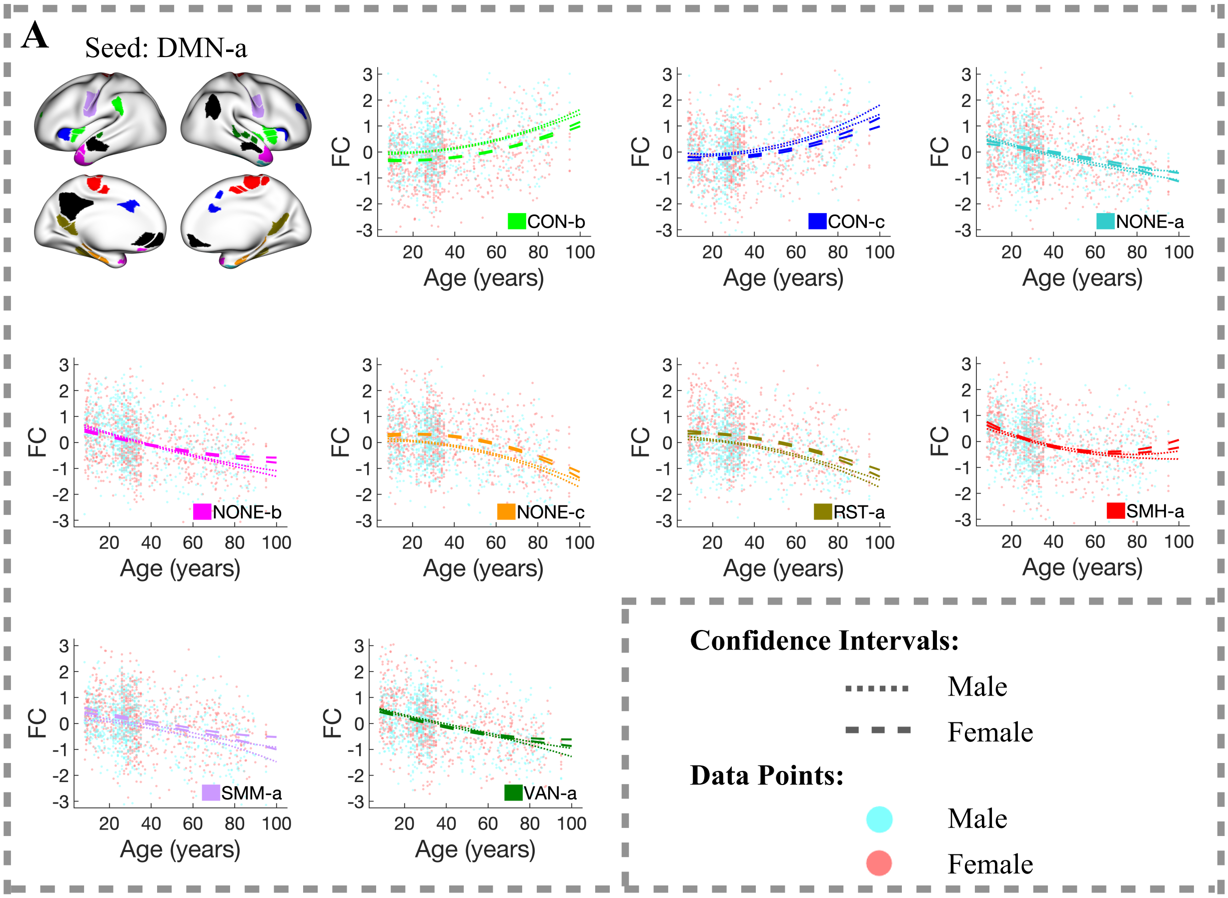

Figure S53: Between-network FC trajectories with R-squared values exceeding 10% using the Gordon parcellation. In each brain plot, one region cluster is designated as the seed cluster, plotted in black. The FC trajectory between the seed cluster and a cluster from the same functional network, referred to as cluster B, is displayed in the color assigned to cluster B. Light cyan and light coral dots in the FC plots represent FC values between a region in the seed cluster and another region in cluster B for individual male and female subjects, respectively. Dotted lines represent 95% confidence intervals for population-mean FC trajectories of males, whereas dashed lines represent those of females.
